# Heteroleptic [Cr^III^N_6_] Chromophores as Partners for Lanthanide-Based Light Conversion in d-f Molecular Complexes

**DOI:** 10.3390/molecules31122016

**Published:** 2026-06-09

**Authors:** Julien Chong, Inès Taarit, Laure Guénée, Arnulf Rosspeintner, Claude Piguet

**Affiliations:** 1 Department of Inorganic and Analytical Chemistry, University of Geneva, 1211 Geneva, Switzerland; julien.chong@unige.ch (J.C.), ines.taarit@unige.ch (I.T.); 2 Laboratory of X-Ray Crystallography, University of Geneva, 1211 Geneva, Switzerland; laure.guenee@unige.ch; 3 Department of Physical Chemistry, University of Geneva, 1211 Geneva, Switzerland; arnulf.rosspeintner@unige.ch

**Keywords:** chromium, lanthanide, heterometallic, coordination chemistry, light conversion

## Abstract

The connection of a dianionic 2,2’-biimidazolate (biim^2−^) bridging unit to *cis*-[Cr(N^∩^N)_2_]^3+^ (N^∩^N is a chelating didentate ligand) or *cis*-[Cr(N^∩^N^∩^N^∩^N)]^3+^ building blocks (N^∩^N^∩^N^∩^N is a chelating tetradentate ligand) produces heteroleptic pseudo-octahedral [CrN_6_]^+^ chromophores. Their reduced cationic charge is compatible with the subsequent complexation of trivalent lanthanides (Ln^3+^) to give d-f {[(N^∩^N)_2_Cr(biim)]*_n_*Ln}^(3+*n*)+^ (*n* = 1–4), {[(N^∩^N)_2_Cr(biim)]Ln(Tp)_2_}^2+^ and {[(N^∩^N^∩^N^∩^N)Cr(biim)]Ln(Tp)_2_}^2+^ adducts (Tp is tri(1*H*-pyrazol-1-yl)-λ^4^-borate). Moving from polyaromatic N^∩^N (1,10 phenanthroline) to saturated N^∩^N^∩^N^∩^N polyamine (cyclam) receptors controls the photophysical properties and leads to tunable light conversion in the target heterometallic complexes when Eu(III) is exploited as the activator for downshifting and Er(III) as the activator for upconversion.

## 1. Introduction

**Figure 1:**
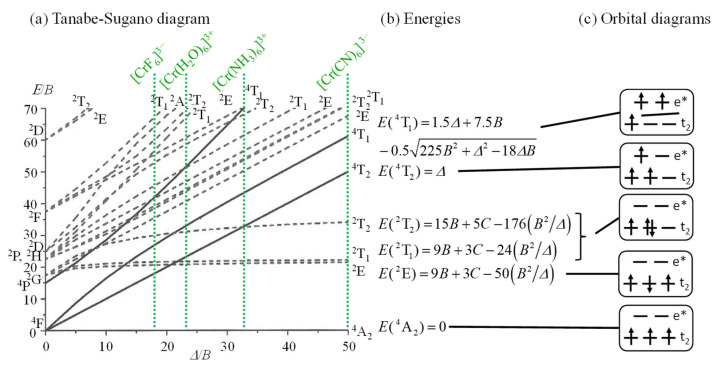
(**a**) Tanabe–Sugano diagram for the d^3^ electron configuration in an octahedral crystal field computed with *C*/*B* = 4.7 and showing spin-quartet (full traces) and spin-doublet (dashed traces) states [5]. (**b**) Energies of the atomic terms derived from the diagonal elements of the Tanabe–Sugano matrices limited to second-order corrections [4,6] and (**c**) associated strong-field orbital illustrations.

The well-known Tanabe–Sugano diagram established for pseudo-octahedral [MX_6_] scaffolds possessing an open-shell d^3^ electronic configuration [1,2,3] applies to [Cr^III^X_6_] chromophores [4,5,6] (Figure 1), and justifies the challenging efforts undertaken in the last two decades for combining them with open-shell 4f-block trivalent cations. Firstly, the ground-state atomic term Cr(^4^A_2_) is magnetically active and behaves as a ‘pure’ multiple spin center (*S* = 3/2) with (i) negligible magnetic anisotropy, (ii) modulable zero-field splitting, and (iii) long electronic relaxation time [7]. When Cr(III) centers are integrated into polynuclear assemblies exhibiting intermetallic ferromagnetic exchange coupling, the magnetic moment of Cr(III) can be exploited for the design of slow-relaxing single molecular magnets with accessible blocking temperatures [8,9,10]. Some specific arrangements of ferromagnetically coupled d-f chromium–lanthanide wheels and butterflies gave access to rare magneto-structural maps [11,12,13,14,15,16]. Alternatively, antiferromagnetic Cr(III)-Gd(III) coupling is highly desired for the preparation of isotropic ultra-dense assemblies inducing large magneto-caloric cooling effects [17,18,19]. The concomitant long electronic relaxation time makes the basis for (i) electron paramagnetic resonance imaging (EPRI) [20], the counterpart of (nuclear) magnetic resonance imaging (MRI), and (ii) molecular quantum bits with long coherence times [21]. Finally, the ground-state d^3^ electronic configuration is associated with the largest ligand-field reorganization energies accompanying ligand exchange processes in octahedral complexes [22,23]. [Cr^III^X_6_] units are therefore kinetically inert, a rare situation for 3d metal complexes, but which is highly desirable for the rational design of heterometallic d-f assemblies with no statistical scrambling in solution [24,25,26]. 

For pseudo-octahedral Cr(III) complexes, there are two types of excited states. The first category corresponds to quartet excited states (*S* = 3/2) resulting from the promotion of one or more electrons from the ground t_2_ orbitals into the anti-bonding e^*^ orbitals with no spin-flip (full traces in Figure 1a). Upon electromagnetic excitation, the latter transitions obey the spin rule, but their absorption cross-sections are still limited by the parity (Laporte) rule (d↔d transitions are magnetically allowed but electric-dipole transitions are forbidden). Interestingly, the resulting distorted quartet excited states, when their lifetimes are long enough, can be exploited to induce photochemical transformations [27,28]. The second category refers to doublet excited states (*S* = ½), where one electron undergoes a spin-switch upon light excitation (dashed traces in Figure 1a). The three lowest-energy spin-flip transitions with *E*/*B* < 30, i.e., Cr(^2^E,^2^T_1_,^2^T_2_←^4^A_2_) in Figure 1a, defy the spin rule and do not involve ligand-field promotion (Figure 1c). They therefore provide more limited potential for photochemistry [29,30], but are highly sought for inducing long-lived near-infrared emission [31] and tunable photophysical properties controlled by the *Δ*/*B* ratio imposed by the surrounding donor atoms [29,30,31,32,33,34,35,36,37]. 

**Figure 2:**
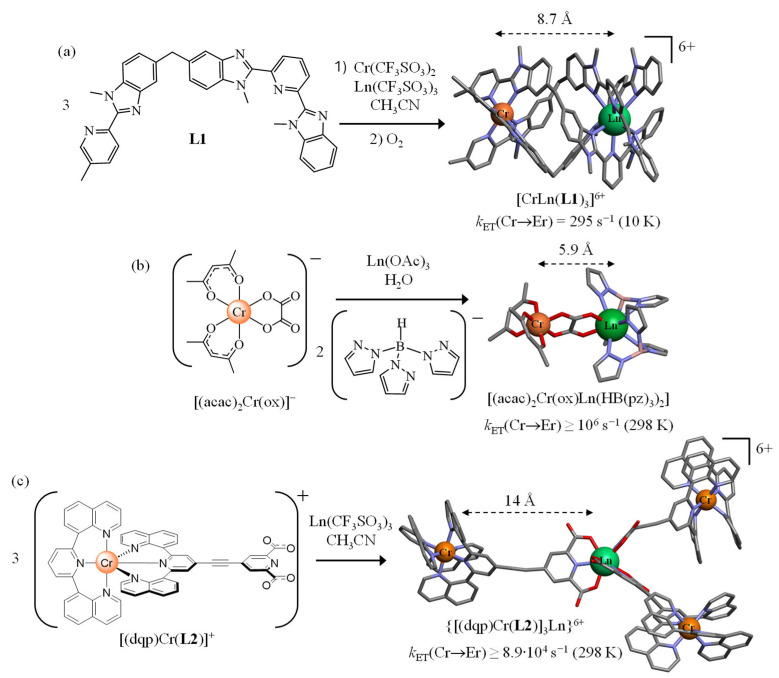
Synthesis of molecular structures of the three heterometallic LnCr systems (Ln = Er) in which first-order intermetallic energy transfer *k*_ET_(Cr↔Er) rate constants were quantified in isolated molecular complexes. (**a**) A self-assembled [CrLn(**L1**)_3_]^6+^ helicate [59], (**b**) a [(acac)_2_Cr(ox)Ln(HB(pz)_3_)_2_] dyad [57,62] and (**c**) a {[(dqp)Cr(**L2**)]_3_Ln}^6+^ tetranuclear adduct [63].

Combined with the wealth of narrow spectroscopic levels characterizing the trivalent lanthanides, Ln^3+^, possessing [Xe]4f*^n^* (*n* = 1–13) electronic configurations [38], d-f Cr(III)/Ln(III) mixtures are regularly doped into ionic solids and nanoparticles for preparing advanced optical materials in which intermetallic sensitizer-to-activator energy transfers modulate luminescence with the goal of inducing light downshifting [39,40,41,42,43,44,45,46,47] or light upconversion [48,49]. Moving toward strict stoichiometric control with satisfying reproducibility requires that the two different metals possess well-defined coordination environments mastered by specific ligands, a target not accessible for doped ionic solids. Moreover, chromium-doped ionic solids are usually restricted to weak-crystal-field [CrO_6_] chromophores with 21 < *Δ*/*B* < 30 as illustrated in ruby (*Δ*/*B* = 24 [50]), whereas a pertinent exploitation of spectrochemical [51] and nephelauxetic [37,52] series covers the 18 < *Δ*/*B* < 50 range in designed coordination complexes ([CrO_6_], [CrN_6_], and [CrC_6_] chromophores or a combination of them; Figure 1a). A first step toward the design of stoichiometric Cr(III)/Ln(III) sensitizer–activator pairs exploits ionic solids made of ion-paired complexes in [Cr(en)_3_][Eu(dipic)_3_] (en = ethane-1,2-diamine, dipic = pyridine-2,6-dicarboxylic acid), the latter exhibiting light downshifting [53], and in [Cr(ddpd)_2_][Yb(dipic)_3_] (ddpd = *N*,*N*’-dimethyl-*N*,*N*’-dipyridine-2-ylpyridine-2,6-diamine), which is active for light upconversion [54]. Closer to molecular systems, the coordination polymer {[(CN)_4_Cr(*μ*-CN)_2_Yb(H_2_O)_2_(dmf)_4_]·H_2_O} _∞_ possesses Cr(*μ*-CN)_2_Yb units, in which the two metals are connected by cyano bridges (Cr···Yb = 5.58 Å), thus ensuring efficient Cr→Yb energy transfers (*k*_ET_ ≥ 10^8^ s^−1^) and subsequent light downshifting [55]. Preparing molecular heterometallic Cr-4f complexes in solutions remains extremely rare [56], and this is despite the attractive large intermetallic communications operating via energy transfers over long distances [57]. We are aware of only three systems in which molecular Cr↔Ln energy transfers were quantified in isolated complexes. The first approach exploits a thermodynamic self-assembly process to assemble labile Cr^2+^ and Ln^3+^ cations with multidentate segmental ligands to give the triple-helical [Cr^II^Ln(**L1**)_3_]^5+^ complex, which is oxidized to give inert [Cr^III^Ln(**L1**)_3_]^6+^ helicates (Figure 2a) [56,58]. The Cr···Ln distance of 8.7 Å, combined with the lack of short bridging ligands, limits the intermetallic energy transfer rate constants to *k*_ET_(Cr→Er) = 10^2^–10^3^ s^−1^. The resulting long Cr(III)-based lifetime was then exploited for inducing unprecedented multi-sensitizer-based energy transfer upconversion (ETU) in the related trinuclear CrErCr helicates [56,58,59]. 

A second method relies on the complex-as-ligand strategy, for which a kinetically inert [Cr^III^X_6_] unit, a common characteristic of pseudo-octahedral d^3^ chromophores [22,23], integrates a bridging ligand designed for the complexation of a trivalent lanthanide metal. This approach was applied for the preparation of [(acac)_2_Cr(ox)Ln(HB(pz)_3_)_2_] (X = O, Figure 2b) [57,60] and of {[(dqp)Cr(**L2**)]_3_Ln}^6+^ (X = N, Figure 2c) adducts [61]. The presence of electron-rich bridging ligands connecting the two metals in the latter adducts boosts the energy transfer processes via the Dexter mechanism [62,63]. The associated rate constant for a Cr···Er distance as long as 14 Å reaches *k*_ET_(Cr→Er) ≈ 10^5^ s^−1^ (Figure 2c).

**Figure 3:**
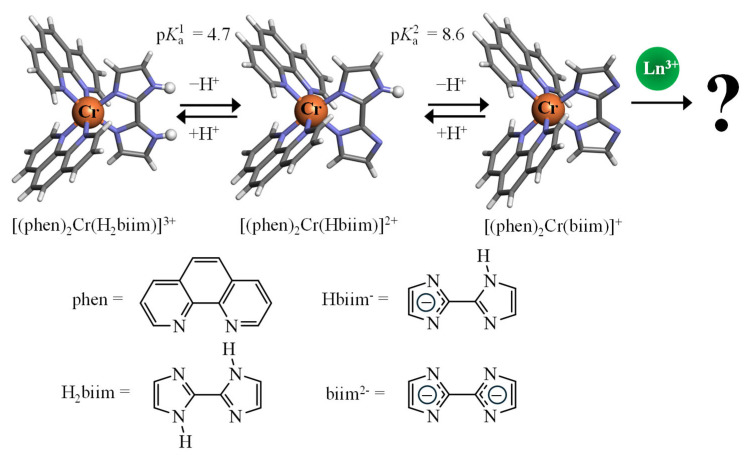
Successive deprotonations of the heteroleptic [(phen)_2_Cr(H_2_biim)]^3+^ complex in water showing the accessible acidic protons as white spheres. The molecular structures correspond to those observed by X-ray diffraction in the solid state [64].

Aiming at maximizing intramolecular *k*_ET_(Cr→Ln) rate constants for rising Visible to NIR downshifting with earth abundant metals, we prepared the heterometallic complex-as-ligand [(phen)_2_Cr(H_2_biim)]^3+^, for which the 2,2′-biimidazolate bridge found in its doubly deprotonated [(phen)_2_Cr(biim)]^+^ form is famous for efficiently connecting additional d-block metals (Figure 3) [64]. However, and to the best of the authors’ knowledge, no connection between Cr(III) and Ln(III) in d-f dyads exploiting this promising electron-rich bridge has been reported. The binding of [(phen)_2_Cr(biim)]^+^, or derivatives of it, working as complex-as-ligand with trivalent lanthanides is therefore explored in this contribution (Figure 3, right).

## 2. Results and Discussions

### 2.1. Reaction of [(phen)_2_Cr(biim)]^+^ Complex-as-Ligand with M(O_3_SCF_3_)_n_ (M = La, Lu, Zn)

**Figure 4:**
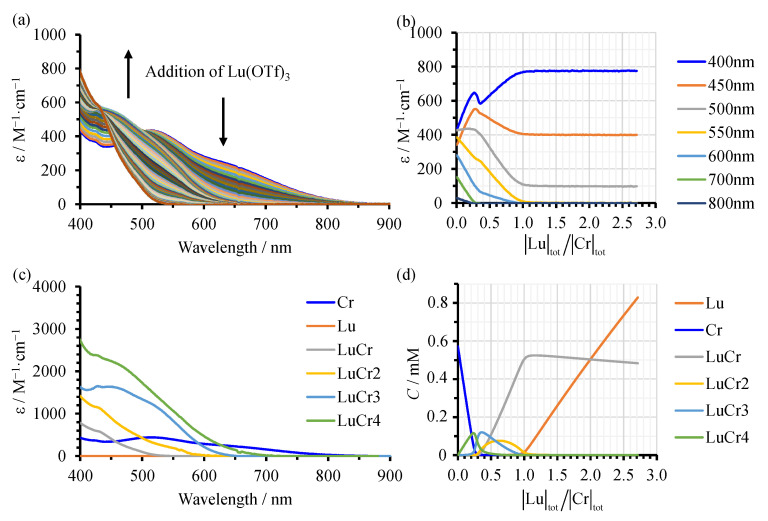
Evolutions of (**a**) absorption spectra (normalized for the total concentration of Cr) and (**b**) related absorbance at selected wavelengths after each addition of Lu(OTf)_3_ to [(phen)_2_Cr(biim)](OTf) (6·10^−4^ M in CH_3_CN). (**c**) Extracted absorption spectra for the absorbing species (Cr and LuCr*_n_*) and (**d**) evolution of concentrations of each species along the titration.

The complex [(phen)_2_Cr(biim)]^+^ possesses biim^2−^→phen ligand-to-ligand charge transfer (LLCT) bands in the visible part of the electromagnetic spectrum, which are shifted toward higher energy upon coordination of the biimidazolate unit to an electron-deficient guest such as H^+^ [64]. The complexation of related metallic cations M*^z^*^+^, instead of H^+^, to the biimidazolate bridge of [(phen)_2_Cr(biim)]^+^ can thus be investigated using spectrophotometric titrations. The stepwise additions of the desired lanthanide triflate Ln(O_3_SCF_3_)_3_ (Ln = La, Lu, O_3_SCF_3_^−^ = OTf^−^ in the rest of this work) to a solution of the complex [(phen)_2_Cr(biim)](OTf) (6 × 10^−4^ M in CH_3_CN) affect the absorption spectra of the solution, with a global blue shift in LLCT transitions covering the visible domain (Figure 4, Figures S1 and S2). Ln = La and Lu have been selected because they are (i) diamagnetic and compatible with parallel NMR titrations and (ii) correspond to the extreme ionic radii along the lanthanide series. Interestingly, the absorbances of the solution at 700 nm and 800 nm rapidly decrease and reach zero for 0.25 equivalents of added Ln^3+^ (Figure 4b and Figure S1a,b), in agreement with the formation of {Ln[(biim)Cr(phen)_2_]_4_}^7+^ as the ultimate complex. A model, where up to four chromium complexes can bind to each Ln^3+^, is proposed according to Equations (1)–(4) (Cr denotes [(phen)_2_Cr(biim)]^+^ and charges are omitted for clarity):

(1) $$\begin{array}{c} \mathrm{Ln}+\mathrm{Cr}\;⇆\;\mathrm{Ln}\mathrm{Cr} \end{array}$$

(2) $$\begin{array}{c} \mathrm{Ln}+2\;\mathrm{Cr}\;⇆\;\mathrm{Ln}{Cr}_{2} \end{array}$$

(3) $$\begin{array}{c} \mathrm{Ln}+3\;\mathrm{Cr}\;⇆\;\mathrm{Ln}{Cr}_{3} \end{array}$$

(4) $$\begin{array}{c} \mathrm{Ln}+4\;\mathrm{Cr}\;⇆\;\mathrm{Ln}{Cr}_{4} \end{array}$$

Each complexation reaction is associated with its association constant $\beta_{1,n}^{Ln,Cr}$:

(5) $$\begin{array}{c} \beta_{1,n}^{Ln,Cr}=\frac{\left[{LnCr}_{n}\right]}{{\left[Cr\right]}^{n}\times \left[Ln\right]}\; \end{array}$$

The spectrophotometric data (Figure 4a,b and Figure S1a,b) could be fitted to equilibria (1)–(4) using Evolving Factor Analysis [65,66] followed by non-linear least-squares techniques as implemented in the software ReactLab™ [67,68], which ultimately provided (i) the absorption spectra of the five individual absorbing species (Cr, LuCr*_n_*, Figure 4c and Figure S1c), (ii) their association constants $\beta_{1,n}^{Ln,Cr}$ (Table 1) and (iii) pertinent speciations in solution (Figure 4d and Figure S1d).

As expected, the addition of Ln^3+^ shifts the LLCT bands to higher energy since it acts as an electro-attractor group on the biimidazolate ligand. The higher the Ln/Cr ratio, the larger is the blue shift according to LnCr > LnCr_2_ > LnCr_3_ > LnCr_4_ > Cr because the electro-attractive character of Ln^3+^ is reduced upon successive binding to several anionic biimidazolate binding units. One, however, notices that the absorption spectra extracted for LaCr_4_, LaCr_3_ and LaCr_2_ (Figure S2c) are slightly more red-shifted compared to the corresponding ones for LuCr_4_, LuCr_3_ and LuCr_2_ species (Figure 4c) because La^3+^ is a weaker electron attractor than Lu^3+^ due to its larger ionic radius (Table 1, entry 7). For comparison purposes, similar titrations using Zn(OTf)_2_, which is also diamagnetic but less charged and smaller than La^3+^ and Lu^3+^, showed that a maximum of three [(phen)_2_Cr(biim)]^+^ complex-as-ligands can be coordinated to a single Zn^2+^ center (Table 1, column 3 and Figure S2).

The interpretation of the association constants $\beta_{1,n}^{M,Cr}$ (M = Lu, La and Zn) relies on the site-binding model where $\beta_{1,n}^{M,Cr}=K_{stat}\times K_{chem}$ [69]. *K*_stat_ accounts for the purely statistical contribution produced by the change in rotational entropies [70]. It is obtained using the symmetry number method (see Supplementary File S1). *K*_chem_ refers to the chemical processes involved in the association process, which can be split into the intermolecular affinity ${\Delta G}_{M,Cr}=-RTln\left(f_{M,Cr}\right)$ between the metal (M) and the complex-as-ligand (Cr), and the intramolecular interaction between the two complex-as-ligands ${\Delta E}_{Cr,Cr}=-RTln\left(u_{Cr,Cr}\right)$ bound to the same metallic center (La^3+^, Lu^3+^ or Zn^2+^). Assuming that Δ*E*_Cr,Cr_ is constant for a given metal, no matter the geometric arrangement of the ligands in the selected MCr*_n_* complex, one can write:

(6) $$\begin{array}{c} \beta_{1,n}^{M,Cr}=K_{stat}\times {\left.{\left(f\right.}_{M,Cr}\right)}^{n}\times ({\left.u_{Cr,Cr}\right)}^{\frac{\left(n^{2}-n\right)}{2}} \end{array}$$

The exponents *n* and (*n*^2^ − *n*)/2 in Equation (6) represent the number of metal–ligand interactions and ligand–ligand interactions in the MCr*_n_* complex, respectively. Transformed into free energy, Equation (6) becomes:

(7) $$-RT\left[ln\left(\beta_{1,n}^{M,Cr}\right)-ln\left(K_{stat}\right)\right]=n{\Delta G}_{M,Cr}+\frac{\left(n^{2}-n\right)}{2}\left({\Delta E}_{Cr,Cr}\right)$$

which can be solved for Δ*G*_M,Cr_ and Δ*E*_Cr,Cr_ with M = Zn (Equation (8)) or M = Ln (Equation (9)) using matrix formulations and multilinear least-squares methods (Table 1, entries 5,6):

(8) −RTlnβ1,1Zn,Crlnβ1,2Zn,Crlnβ1,3Zn,Cr−ln 24ln120ln64=ΔGZn,Cr123+ΔECr,Cr013

(9) −RTlnβ1,1Ln,Crlnβ1,2Ln,Crlnβ1,3Ln,Crlnβ1,4Ln,Cr−ln 32ln288ln702ln224=ΔGLn,Cr1234+ΔECr,Cr0136

For all metals, Δ*G*_M,Cr_ is negative and represents the driving force of the intermolecular complexation reaction, while Δ*E*_Cr,Cr_ is positive, which points to stepwise anti-cooperative connections of cationic [(phen)_2_Cr(biim)]^+^ complex-as-ligands to the central M*^z^*^+^ centers. In fact, Δ*E*_Cr-Cr_ increases when the ionic radius of the metal decreases, but these values remain one order of magnitude smaller than the absolute value of Δ*G*_M,Cr_ and are comparable with thermal energy at room temperature.

**Figure 5:**
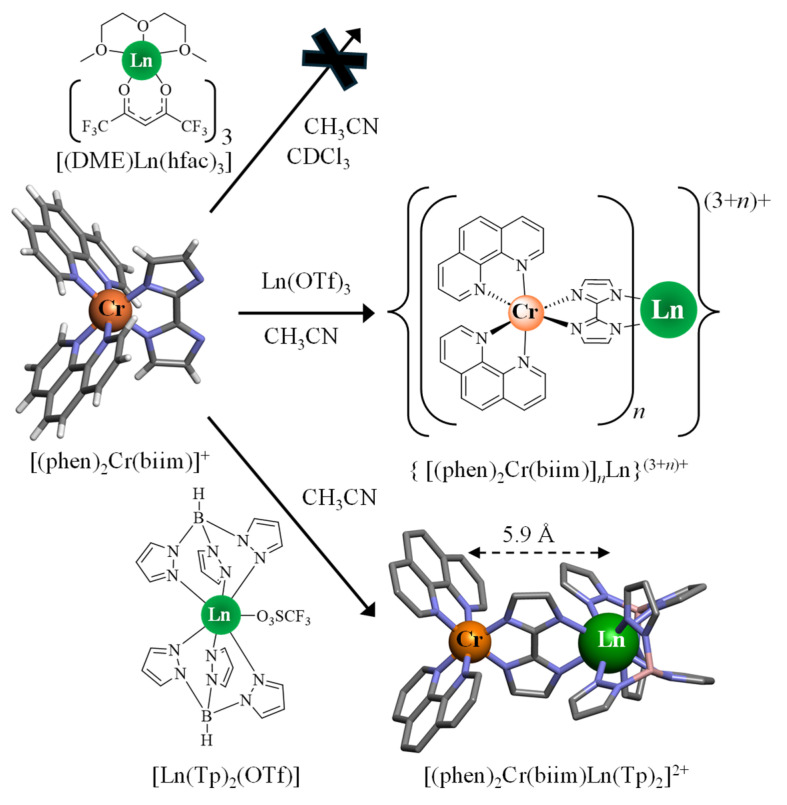
Complex-as-ligand strategies using [(phen)_2_Cr(biim)]^+^ for coordinating [(DME)Ln(hfac)_3_] (top), Ln(OTf)_3_ (center) and [Ln(Tp)_2_(OTf)] (bottom) lanthanide adducts. The molecular structures of [(phen)_2_Cr(biim)]^+^ (CCDC-2355650) [64] and [(phen)_2_Cr(biim)Ln(Tp)_2_]^2+^ (this work) are those observed by X-ray diffraction in the solid state. Color code: C = gray, N =blue, H = white, and B = pink.

Apart from the spectrophotometric titrations, the MCr*_n_* complexes lack any additional characterizations. ESI-MS studies demonstrate that the cationic {Ln[(biim)Cr(phen)_2_]*_n_*}^(3+*n*)+^ aggregates do not survive in (or cannot be transferred into) the gas-phase. In solution, the slow-relaxing electron-spin paramagnetic Cr(III) centers induce fast nuclear-spin relaxation and severe line broadening, rendering the NMR signals of nearby nuclei undetectable; this is in line with the experimental ^1^H-NMR spectrum of [Cr(phen)_2_(biim)]^+^ being featureless [64]. Furthermore, the association constants deduced from spectrophotometric titrations show that intricate mixtures of different MCr*_n_* species are present in solution at millimolar concentrations, and this is regardless of the M:Cr stoichiometry (Figures S3–S5). This drastically complicates selective crystallization processes, and attempts to obtain single crystals of these assemblies suitable for X-ray diffraction (XRD) were unsuccessful (Figure 5, center). With this in mind, our efforts were redirected toward the preparation of Ln-Cr dyads, where both Ln and Cr centers are coordinatively saturated (Figure 5 top and bottom).

### 2.2. Reaction of [(phen)_2_Cr(biim)]^+^ Complex-as-Ligand with Ln(Tp)_2_(OTf) (Ln = Eu, Y)

To isolate and characterize 1:1 [LnCr] dyads, we planned to react [(phen)_2_Cr(biim)]^+^ guests with a lanthanide host complex bearing ancillary ligands. The starting salts [Ln(hfac)_3_dig] (hfac = 1,1,1,5,5,5-hexafluoro-pentane-2,4-dione) have been widely used in our group for this purpose and were considered as suitable precursors for the formation of {[(phen)_2_Cr(biim)]Ln(hfac)_3_}^+^ [71]. However, these [Ln(hfac)_3_] units proved to be unstable in polar solvents such as acetonitrile due to ligand scrambling [71]. This prevented clean reactions with [(phen)_2_Cr(biim)]^+^, which is only soluble in polar solvents (Figure 5, top). To overcome this limitation, we turned our attention toward the alternative lanthanide [Ln(Tp)_2_(OTf)] host (Tp = hydridotris(1*H*-pyrazol-1-yl)borate, Figure 5, bottom) [72] that *Kaizaki* and coworkers reacted with [(acac)_2_Cr(ox)]^−^ complex-as-ligand in polar solvents to give dinuclear [(acac)_2_Cr(ox)Ln(Tp)_2_] dyads (Ln = La, Nd, Eu, Gd, Tb, Dy, Ho, Er, Tm, Yb, Lu, acac^−^ = pentane-2,4-dionate, Figure 2b) [57,60,73,74]. [Ln(Tp)_2_(OTf)] (Ln = Eu, Y) were thus reacted with [(phen)_2_Cr(biim)]^+^ in acetonitrile to form the heterometallic complexes [(phen)_2_Cr(biim)Ln(Tp)_2_]^2+^, which could be characterized in solution (^1^H-NMR, HRMS and spectrophotometry) and in the solid state (XRD).

**Figure 6:**
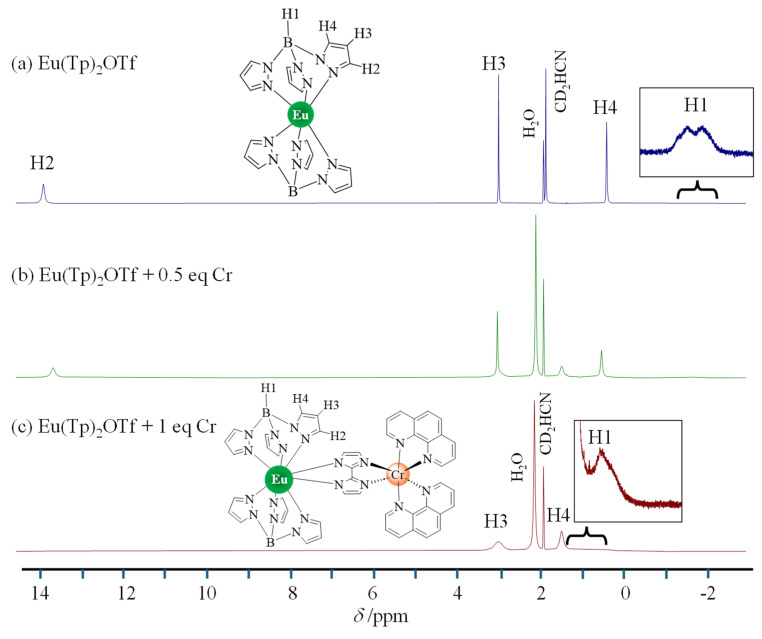
^1^H-NMR spectra recorded for (**a**) Eu(Tp)_2_(OTf), (**b**) Eu(Tp)_2_(OTf) + 0.5 eq [(phen)_2_Cr(biim)](OTf) and (**c**) Eu(Tp)_2_(OTf) + 1 eq [(phen)_2_Cr(biim)](OTf) in CD_3_CN at 293 K.

Contrary to the slow-relaxing paramagnetic LnCr*_n_* assemblies discussed above, the fast-relaxing [Eu(Tp)_2_]^+^, or even diamagnetic [Y(Tp)_2_]^+^ moiety, possesses protons for which the ^1^H-NMR signal can be easily tracked. Upon stepwise titrations with slow-relaxing paramagnetic [(phen)_2_Cr(biim)]^+^ complex-as-ligand, the observed distance-dependent increase in line broadening of the ^1^H NMR signals along the series Cr···H4 < Cr···H3 < Cr···H2 (Ln = Eu in Figure 6c and Ln = Y in Figure S6c) can be considered as solid proof of the coordination of the chromium moiety to the lanthanide cargo and the formation of the LnCr dyad in solution.

High-resolution mass spectra (HRMS) recorded for solutions of [(phen)_2_Cr(biim)]OTf/Ln(Tp)_2_OTf with various stoichiometric ratios in CH_3_CN (Ln = Eu, Y) confirm the formation of 1:1 stoichiometric dyads displaying well-resolved signals assigned to [(phen)_2_Cr(biim)Ln(Tp)_2_]^2+^ and {[(phen)_2_Cr(biim)Ln(Tp)_2_]OTf}^+^ (Figures S7–S12). The formation of the [(phen)_2_Cr(biim)Ln(Tp)_2_]^2+^ dyad is accompanied by an expected color change in the solution induced by the blue shift in the biim^2−^→phen LLCT transition (Figures S13a and S14a) upon complexation, as previously discussed for the formation of {Ln[(biim)Cr(phen)_2_]*_n_*}^(3+*n*)+^ complexes (Figure 4a). We therefore similarly exploited spectrophotometric titrations to quantitively extract the association constants $\beta_{1,1}^{TpY,Cr}$ = 7.6(3) and $\beta_{1,1}^{TpEu,Cr}$ = 8.7(7) (Equation (10), Supplementary File S2):

(10) $$\begin{array}{c} {\left[{Ln(Tp)}_{2}\right]}^{+}\;+{{\left[(phen\right)}_{2}Cr(biim)]}^{+}\;⇆\;{\left[(phen\right)}_{2}\mathrm{Cr}\left(biim\right){{Ln\left(Tp\right)}_{2}]}^{2+}\;\;\; \end{array}\beta_{1,1}^{TpLn,Cr}$$

The resulting quantitative formation of the target [(phen)_2_Cr(biim)Ln(Tp)_2_]^2+^ adducts at millimolar concentrations (Figures S13d and S14d) helped in the selective crystallizations of the heteronuclear [(phen)_2_Cr(biim)Ln(Tp)_2_](OTf)_2_ dyads (Ln = Y, Eu; Figure 5, bottom), the crystal structures of which could be solved by XRD (Supplementary File S3). The lanthanide cation is surrounded by eight nitrogen atoms in the first coordination sphere, two from the biimidazolate bridge and three from each terminal Tp^−^ ligands. The closest geometry of the first coordination sphere was determined using continuous shape measurements [75,76] and elected the square antiprism as the closest polyhedron (ideal symmetry point group *D*_4d_, Figure S31 and Table S14 in Supplementary File S3). The Ln-N bond lengths decrease along Eu > Y > Er, in agreement with the standard lanthanide contraction trend [77]. The Ln-N distances are anecdotally shorter with the bound Tp^−^ ligand than with the biimidazolate bridge (Table S14), and the average Ln···Cr separation amounts to 5.86(2) Å (Ln = Eu, Y, Table S15). The trivalent chromium center adopts the well-known pseudo-octahedral coordination sphere distorted by the formation of three five-membered chelate rings, as previously discussed for [(phen)_2_Cr(H*_n_*biim)]^(*n*+1)+^ [64]. Interestingly, the Cr-N_biim_ distances of 2.041(5) Å in [(phen)_2_Cr(biim)Ln(Tp)_2_] are slightly larger than 2.024(7) Å reported for the fully protonated [(phen)_2_Cr(H_2_biim)]^4+^ complex (Table S16). This fixes the electro-attracting effect of the trivalent lanthanide [Ln(Tp)_2_]^+^ bound to the biimidazolate bridge in the dyads to be comparable, or even slightly larger, than that of two protons in the solid state.

**Figure 7:**
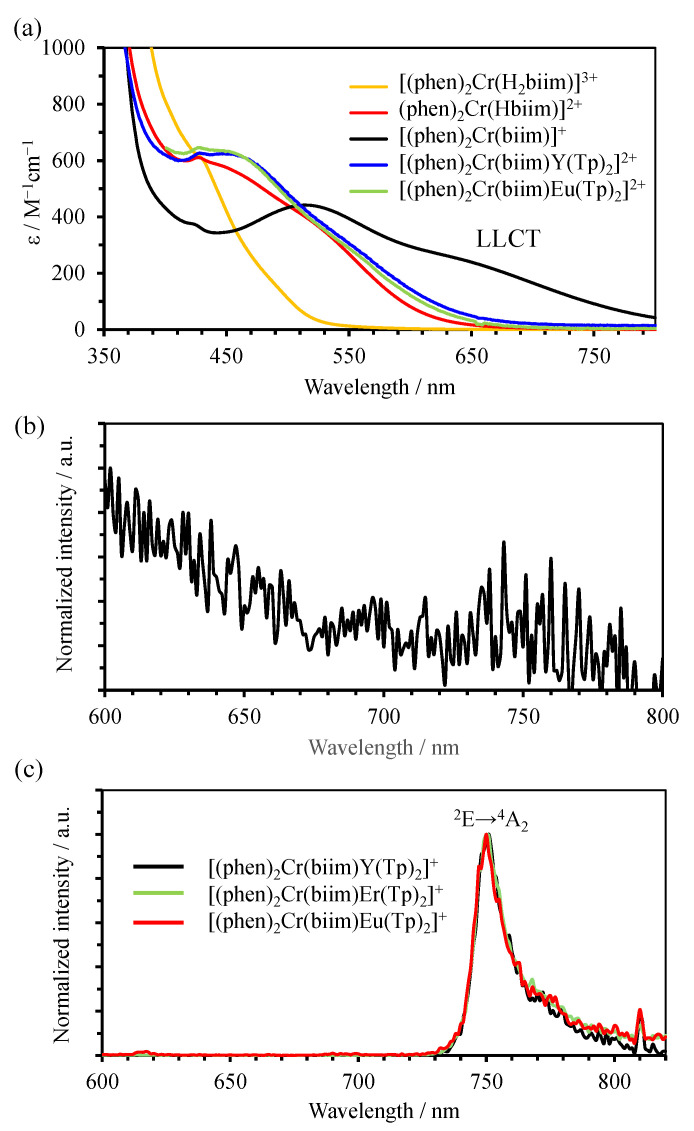
(**a**) Absorption spectra (*c* = 10^−4^ M in CH_3_CN at 293K) of [Cr(phen)_2_(H_2_biim)]^3+^ (orange), [Cr(phen)_2_(Hbiim)]^2+^ (red), [Cr(phen)_2_(biim)]^+^ (black), [(phen)_2_Cr(biim)Y(Tp)_2_]^2+^ (blue) and [(phen)_2_Cr(biim)Eu(Tp)_2_]^2+^ (green). (**b**) R-oom temperature emission spectrum of [(phen)_2_Cr(biim)Y(Tp)_2_]^2+^ (CH_3_CN, λ_exc_ = 350 nm, *c* = 10^−4^ M). (**c**) Low-temperature (77K) emission spectra of [(phen)_2_Cr(biim)Y(Tp)_2_]^2+^ (black), [(phen)_2_Cr(biim)Eu(Tp)_2_]^2+^ (red) and [(phen)_2_Cr(biim)Er(Tp)_2_]^2+^ (green) (CH_3_CN/C_2_H_5_CN 6:4, λ_exc_ = 350 nm, c = 10^−4^ M).

In solution, the absorption spectra recorded for [(phen)_2_Cr(biim)Ln(Tp)_2_]^2+^ (Ln = Y, Eu; Figure 7a) reveal that the ligand-to-ligand charge transfer (LLCT) band of the [(phen)_2_Cr(biim))]^+^ moiety undergoes a blue shift upon coordination to the [Ln(Tp)_2_]^+^ moiety comparable to the fixation of only a single proton to the biimidazolate bridge, as found in [Cr(phen)_2_(Hbiim)]^2+^ (compare the red trace with the blue and green traces in Figure 7a). Consequently, the weak spin-forbidden chromium-centered absorption bands Cr(^2^E←^4^A_2_) and Cr(^2^T_1_←^4^A_2_), expected around 720–750 nm in [(phen)_2_Cr(biim)Ln(Tp)_2_]^2+^ dyads, are masked by the more intense band foot of the LLCT transitions, and could not be identified. As a logical issue, upon excitation in the UV, the searched NIR emission bands arising from Cr(^2^E) and Cr(^2^T_1_) excited states in [(phen)_2_Cr(biim)Ln(Tp)_2_]^2+^ are completely quenched by non-radiative energy transfer toward the broad non-emissive LLCT levels at room temperature (Figure 7b). At 77 K, the non-radiative processes slow down, and the three complexes [(phen)_2_Cr(biim)Ln(Tp)_2_]^+^ (Ln = Y, Eu, Er) become emissive, showing Cr(^2^E→^4^A_2_) phosphorescence at λ_max_ = 750 nm (13333 cm^−1^, Figure 7c).

We conclude that the spin-flip excited states Cr(^2^E) and Cr(^2^T_1_) in [(phen)_2_Cr(biim)Ln(Tp)_2_]^2+^ dyads relax too fast at room temperature to act as donors that efficiently transfer their energy to the lanthanide neighbor working as the energy acceptor. This restricts their potential to be used as sensitizers at room temperature for both light downshifting and light upconversion. The required removal of the deleterious quenching by the low-energy LLCT levels was therefore considered as a priority. We thus decided to investigate similar Cr(III)-Ln(III) dyads, but replacing the phenanthrolines with the saturated cyclam ligand (1,4,8,11-tetraazacyclotetradecane) lacked accessible low-energy π* orbitals.

### 2.3. Synthesis and Characterization of [cyclam)Cr(biim)Ln(Tp)_2_](OTf) (Ln = Eu, Y, Er) Dyads

**Figure 8:**
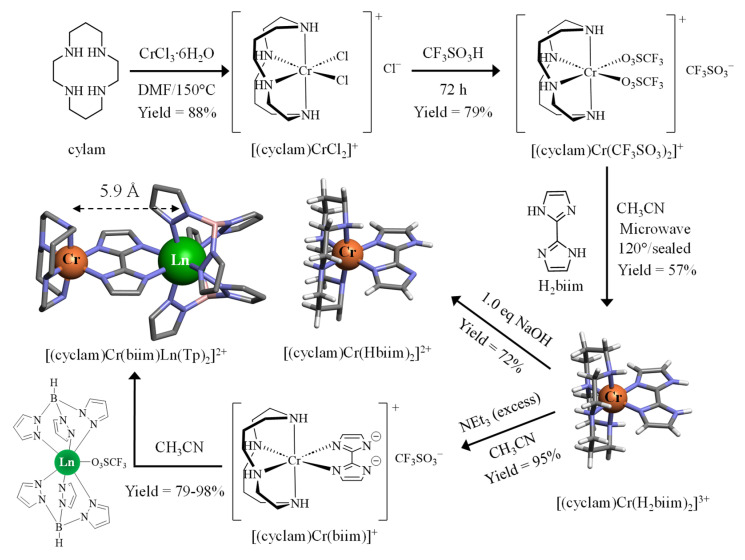
Synthesis of [(cyclam)Cr(H*_n_*biim)]^(*n*+1)+^ (*n* = 0–2) and subsequent complex-as-ligand strategy for coordinating [Ln(Tp)_2_(OTf)] lanthanide cargoes. The molecular structures of [(cyclam)Cr(H_2_biim)]^3+^, [Cr(cyclam)(Hbiim)]^2+^ and [(cyclam)Cr(biim)Ln(Tp)_2_]^2+^ are those observed by X-ray diffraction in the solid state. Color code: C = gray, N =blue, H = white, and B = pink.

The synthesis of the heteroleptic [(cyclam)Cr(H_2_biim)]^3+^ scaffold was performed using the *Kane–Maguire* strategy [24] and following a literature procedure [78]. The starting Cr(III) salt CrCl_3_·6H_2_O was reacted with 0.9 equivalent of cyclam in boiling DMF, producing *cis*-[Cr(cyclam)Cl_2_]Cl as a purple powder (Figure 8). The *cis* configuration is retained thanks to a small excess of CrCl_3_·6H_2_O, which prevents base-catalyzed isomerization to the *trans* isomer [79]. Anion exchange was then performed to convert *cis*-[(cyclam)CrCl_2_]Cl into *cis*-[(cyclam)Cr(OTf)_2_]OTf using triflic acid and releasing HCl gas as a side product. The labile triflates could finally be substituted with a didentate H_2_biim ligand to give the target [(cyclam)Cr(H_2_biim)](OTf)_3_ building block as an orange solid (Figure 8). Recrystallization by vapor diffusion of Et_2_O into a methanolic solution produced crystals suitable for XRD studies (Supplementary File S3, Tables S3 and S4 and Figures S15 and S17).

As observed with [(phen)_2_Cr(H_2_biim)]^3+^ (Figure 3) [64], the acidic protons of the biimidazole ligand in [(cyclam)Cr(H_2_biim)]^3+^ can be removed in basic media. Reaction with one equivalent of NaOH produced [Cr(cyclam)(Hbiim)](OTf)_2_, which could be isolated in good yield as an orange solid (Figure 8) and recrystallized for crystal structure determination (Supplementary File S3, Tables S5 and S6 and Figures S16 and S18). Using an excess of triethylamine in CH_3_CN causes the immediate precipitation of the doubly deprotonated [(cyclam)Cr(biim)]OTf salt as an insoluble orange solid in quantitative yield (Figure 8). Its insolubility in both water and organic solvents prevented the preparation of single crystals suitable for X-ray diffraction. Similarly, it was also impossible to determine the p*K*a’s for the protons of [Cr(cyclam)(H_2_biim)]^3+^ for solubility reasons, but one can reasonably assume values like those measured for [(phen)_2_Cr(H_2_biim)]^3+^ (p*K*a_1_ = 4.67(3), p*K*a_2_ = 8.59(11) in Figure 3) [64].

Heterogeneous mixing of the insoluble salt [Cr(cyclam)(biim)]OTf with [Ln(Tp)_2_(OTf)] in acetonitrile led to their complete dissolution and the formation of the soluble heterometallic complex [(cyclam)Cr(biim)Ln(Tp)_2_]^2+^. The complex could be isolated by precipitation with Et_2_O or by evaporation of the solution to give [(cyclam)Cr(biim)Ln(Tp)_2_](OTf)_2_ in good yield (Figure 8). Recrystallization provided crystals measurable by XRD (Supplementary File S3: Ln = Y, Figure S22 and Table S9; Ln = Eu, Figures S23 and S25 and Table S10; Ln = Er, Figures S24 and S26 and Table S11). As expected, the Cr(III) metal keeps its distorted pseudo-octahedral [CrN_6_] coordination sphere and Er(III) is coordinated by eight nitrogen atoms in [(cyclam)Cr(biim)Ln(Tp)_2_](OTf)_2_ dyads (Figure 8, *d*_Cr-Ln_ = 5.89(1) Å, Table S15) as previously discussed for [(phen)_2_Cr(biim)Ln(Tp)_2_](OTf)_2_ (Figure 5, *d*_Cr-Ln_ = 5.86(2) Å, Table S15). 

In acetonitrile solution, the ^1^H-NMR spectra of the heterometallic complexes [(cyclam)Cr(biim)Ln(Tp)_2_]^2+^ (Ln = Y and Eu) display measurable broad peaks for the protons of the Tp^−^ ligands, because they are sufficiently remote from the slow-relaxing paramagnetic Cr(III) center (Figures S32 and S33), as previously described for the [(phen)_2_Cr(biim)Ln(Tp)_2_]^2+^ dyads. The ^1^H NMR data thus supports that the Cr(III)-containing unit is coordinated to the [Ln(Tp)_2_]^+^ moiety in solution. In addition, millimolar solutions of [(cyclam)Cr(biim)Ln(Tp)_2_]^2+^ analyzed by HRMS (Ln = Y, Eu, Er; Figures S34–S36) systematically exhibit intense signals corresponding to {[(cyclam)Cr(biim)Ln(Tp)_2_]OTf}^+^ (Figures S34–S36).

### 2.4. Photophysical and Light Conversion Properties of [cyclam)Cr(biim)Ln(Tp)_2_](OTf)_2_ (Ln = Eu, Y, Er) Dyads

**Figure 9:**
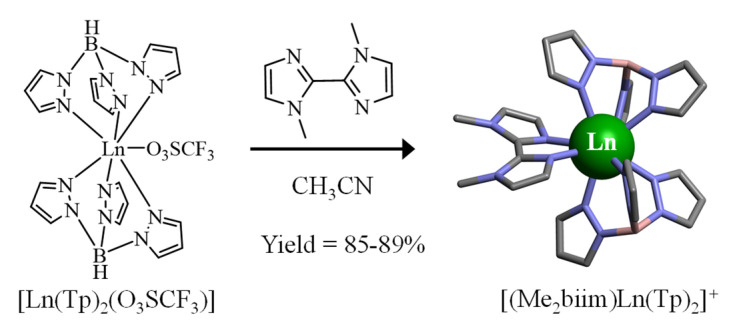
Synthesis of [(Me_2_biim)Ln(Tp)_2_]^+^ (Ln = Y, Er). The molecular structure corresponds to that observed by X-ray diffraction in the solid state for Ln = Y. Color code: C = gray, N =blue, and B = pink.

Having [(cyclam)Cr(H*_n_*biim)]^(*n*+1)+^ (*n* = 0–2) and [(cyclam)Cr(biim)Y(Tp)_2_]^2+^ at hand (Figure 8), the Cr(III)-centered photophysical properties, in the absence of any intermetallic Cr↔Ln energy transfers, are accessible. This is not the case for the [ErN_8_] site, which is only found close to Cr(III) in [(cyclam)Cr(biim)Er(Tp)_2_]^2+^. It was therefore highly desirable to access the photophysical properties of erbium complexes possessing similar coordination spheres, but in the absence of communication with the Cr(III) center. To accurately mimic the coordination environment of the Ln(III) ion, the [(cyclam)Cr(biim)]^+^ unit in [(cyclam)Cr(biim)Ln(Tp)_2_]^2+^ was replaced with the didentate 1,1′-dimethyl-1*H*,1′*H*-2,2′-biimidazole (Me_2_biim) ligand to give [(Me_2_biim)Ln(Tp)_2_]OTf (Figure 9 and Supplementary File S3: Ln = Y, Figures S27 and S29 and Table S12; Ln = Er, Figures S28 and S30 and Table S13). In acetonitrile, the ^1^H-NMR spectrum of diamagnetic [(Me_2_biim)Y(Tp)_2_]OTf demonstrated the selective formation of the heteroleptic complex in solution (Supplementary File S4, Figure S37). Additionally, the HRMS of solutions of [(Me_2_biim)Ln(Tp)_2_]OTf (Ln = Y, Figure S38 and Ln = Er, Figure S39) display peaks of [(Me_2_biim)Ln(Tp)_2_]^+^, thus confirming the formation of the target complexes in solution. The complementary [(Me_2_biim)Eu(Tp)_2_]OTf adduct based on Eu(III) was prepared in situ by stoichiometric mixing of Me_2_biim and [Eu(Tp)_2_(OTf)] in acetonitrile.

**Figure 10:**
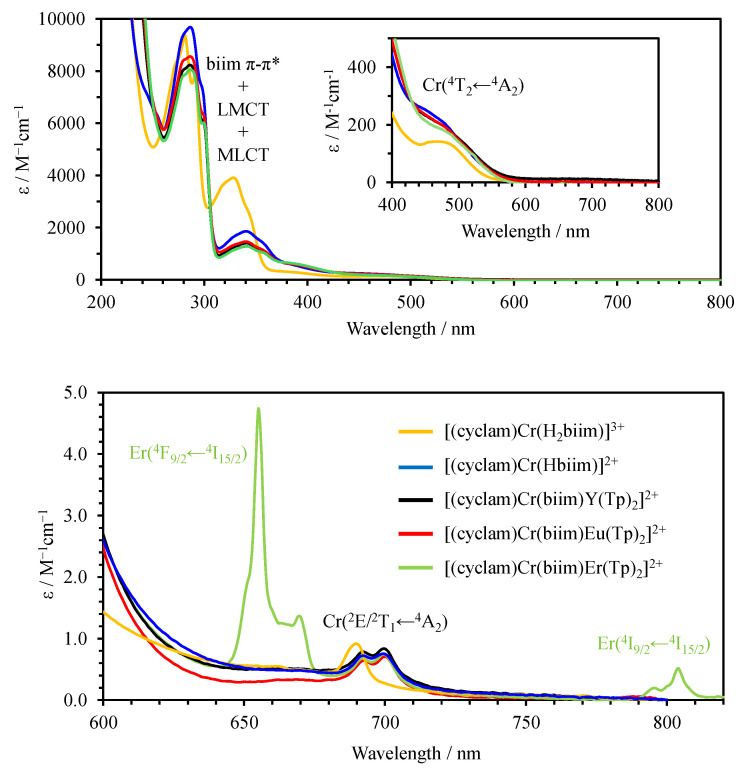
Absorption spectra of [(cyclam)Cr(H*_n_*biim)]^(*n*+1)+^ (*n* = 2, 1) and [(cyclam)Cr(biim)Ln(Tp)_2_]^2+^ (Ln = Y, Eu, Er) in acetonitrile at 293 K with assignments of the transitions.

The absorption spectra of the complexes [(cyclam)Cr(H_2_biim)]^3+^ (yellow traces in Figure 10) and [(cyclam)Cr(Hbiim)]^2+^ (blue traces in Figure 10) could be recorded in acetonitrile, but [(cyclam)Cr(biim)]OTf was too insoluble in all available solvents to access its photophysical properties in solution. The UV part of the absorption spectra displays intense absorption bands (ε > 10^3^ M^−1^·cm^−1^), which correspond to π*←p transitions centered on the aromatic biimidazole ring, completed with charge transfer transitions (LMCT or MLCT, Figure 10, top). Compared with [(phen)_2_Cr(Hbiim)]^2+^ (Figure 7a), the absence of low-energy LLCT charge transfer bands in [(cyclam)Cr(Hbiim)]^2+^ (Figure 10) cleared the 450-800 nm visible domain from intense transitions, thus giving access to the easy detection of the weaker metal-centered spin-allowed Cr(^4^T_2_←^4^A_2_) transition around 500 nm (ε < 500 M^−1^·cm^−1^, Figure 10, top) and spin-forbidden Cr(^2^T_1_,^2^E←^4^A_2_) transition around 700 nm (0.55 M^−1^·cm^−1^ < ε < 0.67 M^−1^·cm^−1^, Figure 10, bottom). 

The [(cyclam)Cr(biim)Y(Tp)_2_]^2+^ dyad exhibits an absorption spectrum like the one of [Cr(cyclam)(Hbiim)]^2+^, thus confirming that the [Ln(Tp)_2_]^+^ group has an electro-attracting strength on the biimidazolate bridge comparable to a single H^+^. These characteristics match those previously observed for [(phen)_2_Cr(biim)Ln(Tp)_2_]^2+^ assemblies (Section 2.2). However, the maximum of the spin-flip Cr(^2^T_1_,^2^E←^4^A_2_) absorption bands is blue-shifted for [Cr(cyclam)(H_2_biim)]^3+^ (*λ*_max_ = 689 nm) and for the related cyclam-based [(cyclam)Cr(biim)Y(Tp)_2_]^2+^ dyads (*λ*_max_ = 700 nm, Figure 10, bottom), compared to their [(phen)_2_Cr(biim)Ln(Tp)_2_]^2+^ analogs at 77 K (*λ*_max_ = 750 nm in Figure 7c). This results from the weaker nephelauxetic effect induced by the aliphatic cyclam ligand, compared to that produced by the polyaromatic phenanthroline units.

The complex [(cyclam)Cr(biim)Er(Tp)_2_]^2+^ additionally exhibits the typical Er(^2*S*+1^*L_J_*←^4^I_15/2_) f-f absorption bands beyond 600 nm (Figure 10, bottom, and Figure S40), which exactly fit those recorded for [(Me_2_biim)Er(Tp)_2_]^+^ (Figure S40). In line with the crystal structures collected in the solid state, one concludes that the didentate ligand Me_2_biim mimics satisfyingly the local environment around the Er(III) ion found upon connection of [(cyclam)Cr(biim)]^+^ complex-as-ligand to Er(Tp)_2_]^+^ for the dyad in solution. Finally, the radiative rate constants *k*_rad_ for the Cr(^2^E/^2^T_1_←^4^A_2_) and Er(^2*S*+1^*L_J_*←^4^I_15/2_) transitions, which are crucial for estimating intrinsic emission quantum yields (vide infra), could be estimated from the absorption spectra using the Strickler-Berg Equation (11) [80,81,82]. They are collected in Table 2 and Table S17:

(11) $$\begin{array}{c} k_{rad}=2303\times \frac{{8\pi cn^{2}\tilde{\nu }}^{2}g_{GS}}{N_{A}g_{ES}}\int \varepsilon \left(\tilde{\nu }\right)\;d\tilde{\nu } \end{array}$$

Here, c is the speed of light in vacuum (cm·s^−1^), *n* is the refractive index of the solvent, N_A_ is Avogadro’s number (mol^−1^), *g*_GS_ is the degeneracy of the ground state, *g*_ES_ is the degeneracy of the excited state, $\tilde{\nu }$ is the barycenter of the transition in wavenumber (cm^−1^) and $\int \varepsilon \left(\tilde{\nu }\right)\;d\tilde{\nu }$ is the area under the absorption spectrum of each transition (M^−1^·cm^−2^). 

**Figure 11:**
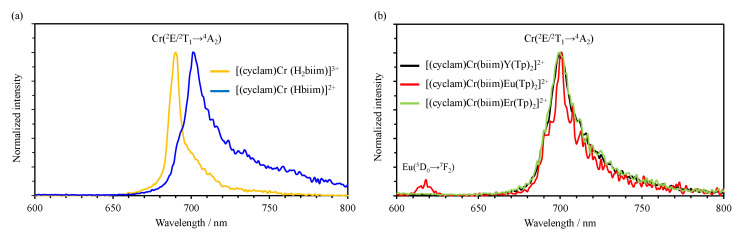
Emission spectra of (**a**) [(cyclam)Cr(H*_n_*biim)]^(*n*+1)+^ (*n* = 2, *λ*_exc =_ 300 nm; *n* = 1, *λ*_exc_ = 350 nm) and (**b**) [(cyclam)Cr(biim)Ln(Tp)_2_]^2+^ (Ln = Y, Eu, *λ*_exc_ = 300 nm; Ln = Er, *λ*_exc_ = 350 nm) in acetonitrile at 293 K with assignments of the transitions.

Upon excitation in the UV, the complexes [Cr(cyclam)(H_2_biim)]^3+^ and [Cr(cyclam)(Hbiim)]^2+^ emit Cr(^2^E,^2^T_1_→^4^A_2_) phosphorescence at 690 nm (14,500 cm^−1^) and 701 nm (14,300 cm^−1^) respectively (Figure 11a). The emission bands are isoenergetic with the absorption bands of the transition ^2^E←^4^A_2_, indicating negligible Stokes shifts (Figure 10). The measured total emission lifetime *τ*_tot_ reaches 1.13 μs and 0.42 μs for [Cr(cyclam)(H_2_biim)]^3+^ and [Cr(cyclam)(Hbiim)]^2+^, respectively, from which the intrinsic quantum yields (*Φ*_intrinsic_) could be estimated using Equation (12), where *k*_rad_ is the radiative rate constant (Table 2):

(12) $$\begin{array}{c} {ɸ}_{intrinsic}=\;\frac{k_{rad}}{k_{rad}{+\;k}_{nonrad}}=k_{rad}\cdot \tau_{tot} \end{array}$$

*Φ*_intrinsic_ = 1.6·10^−4^ found for [Cr(cyclam)(H_2_biim)]^3+^ and *Φ*_intrinsic_ = 5.7 × 10^−5^ for [Cr(cyclam)(Hbiim)]^2+^ are low compared to many other Cr(III) complexes for which the emission quantum yield can be higher than 0.1 at room temperature [31,32,35,83]. This implies efficient non-radiative relaxation pathways tentatively ascribed to the presence of the high-energy N-H bond oscillators of the cyclam ring close to the Cr(III) center [26,32,84]. To test this hypothesis, we tried to synthesize the same complex with the fully *N*-methylated variant of cyclam: Me_4_cyclam, but all our attempts to complex this bulky ligand to Cr(III) proved to be unsuccessful (Supplementary File S5 in the ESI). 

**Figure 12:**
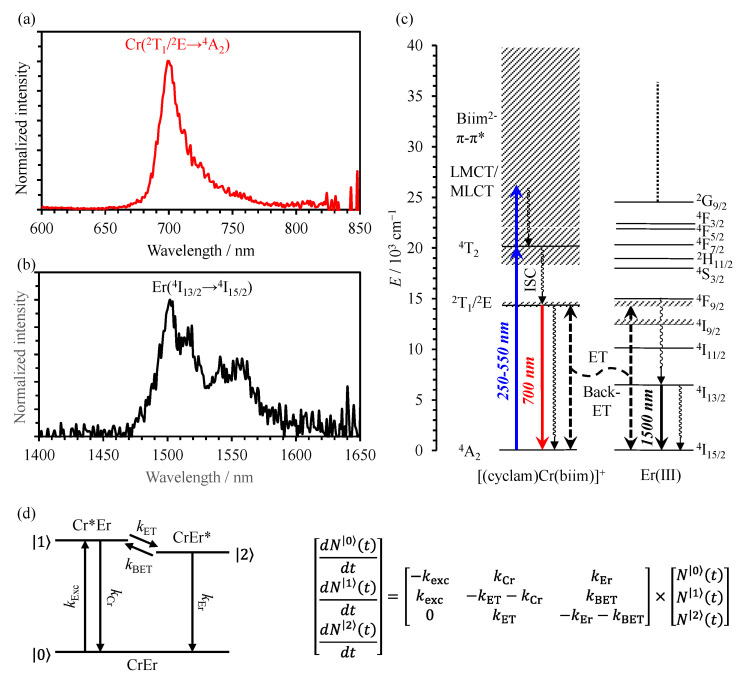
Emission spectra of [(cyclam)Cr(biim)Er(Tp)_2_]^2+^ upon light excitation (**a**) *λ*_exc_ = 300 nm (*c* = 1 mM) and (**b**) *λ*_exc_ = 500 nm (*c* = 10 mM) in acetonitrile at 293 K with assignments of the transitions. (**c**) Jablonski diagram of the energy levels of the Cr(III) complex-as-ligand and Er(III) center in [(cyclam)Cr(biim)Er(Tp)_2_]^2+^ and showing radiative (straight arrows) and non-radiative transitions (wavy arrows) upon UV-Vis excitation. ET = energy transfer; ISC = intersystem. (**d**) Simplified kinetic model with associated first-order kinetic matrix equation.

UV-Visible excitations of the complexes [(cyclam)Cr(biim)Ln(Tp)_2_]^2+^ (Ln = Y, Eu, Er) at room temperature (Figure 11b), or at 77K (Figure S44), result in spin-flip Cr(^2^E/^2^T_1_→^4^A_2_) emissions at *λ*_max_ = 700 nm together with a trace of the forced electric-dipole hypersensitive Eu(^5^D_0_→^7^F_2_) band at 618 nm for the Cr-Eu pair (Figure 11b). The total Cr(^2^E) emission lifetimes of 0.23 ≤ *τ*_tot_ ≤ 0.25 ms recorded for Cr-Y and Cr-Eu dyads are shorter than those gathered for [Cr(cyclam)(H_2_biim)]^3+^ and [Cr(cyclam)(Hbiim)]^2+^ (Table 2), pointing to a global increase in the non-radiative deexcitation rate constant of the latter excited state in the dyads. The further reduction in the lifetime to reach *τ*(Cr(^2^E))_tot_ = 0.1 ms in Cr-Er, combined with the observation of (i) a dual NIR Cr(^2^E/^2^T_1_→^4^A_2_) (Figure 12a) and IR Er(^4^I_13/2_→^4^I_15/2_) luminescence (Figure 12b) and (ii) a biexponential decay of the Cr(^2^E) donor level (Table 2 and Figure S48), suggests the operation of a reversible energy transfer (ET) process connecting the two metallic centers (Figure 12c). The latter assumption is corroborated by (i) the excitation spectrum upon analyzing the Er(^4^I_13/2_→^4^I_15/2_) emission at 1550 nm, which shows the spin-allowed Cr(^4^T_2_→^4^A_2_) transition at 525 nm acting as the main sensitizing channel (Figure S45) and (ii) the successful sensitization of the Er(^4^I_13/2_→^4^I_15/2_) emission by selective excitation of the spin-forbidden Cr(^2^E/^2^T_1_←^4^A_2_) transition using a 698 nm laser beam (Figure S46).

Accordingly, the Jablonski diagram modeling the intermetallic energy transfer implies Cr(^2^E,^2^T_1_) acting as the donor level, while the Er(III)-centered acceptor level may be either ^4^I_9/2_ or ^4^F_9/2_ (Figure 12c). The associated energy diagram built in Figure 12d can be modeled with a set of three linear differential equations written in the matrix form given in Equation (13), further detailed in Figure 12d:

(13) $$\begin{array}{c} \left[\frac{dN^{\left|i\right\rangle }}{dt}\right]=M\times \left[N^{\left|i\right\rangle }\right] \end{array}$$

Following an initial pulsed excitation, the solution of the differential Equation (13) modeling the biexponential time-dependent relaxation of the Cr^*^Er level (referred to as $\left|1\right\rangle$ in the associated energy diagram of Figure 12d) is obtained using the Lagrange-Sylvester formula (Supplementary File S6) [59,85]. Reasonably fixing *k*_Cr_ as the inverse of the total emission lifetime recorded for Cr-Y (*k*_Cr_ = 1/τ_tot_ = 4.0 × 10^6^ s^−1^, Table 2), the three other constants *k*_Er_ = 1.2 × 10^6^ s^−1^, *k*_ET_ = 4.3 × 10^6^ s^−1^ and *k*_BET_ = 2.5 × 10^6^ s^−1^ could be estimated by a non-linear least-square fit of the relative population *N*^(|1⟩^(*t*) (computed with the Lagrange-Sylvester formula) to the experimental biexponential emission trace of the Cr(^2^E,^2^T_1_) excited level observed for the Cr-Er dyad (Figure S48 in Supplementary File S6). The Cr→Er energy transfer rate constant *k*_ET_ = 4.3 × 10^6^ s^−1^ extracted with this method for [(cyclam)Cr(biim)Er(Tp)_2_]^2+^ (*d*_Cr-Er_ = 5.9 Å, Table S15) is circa 50 times larger than the value of *k*_ET_ = 8.96 × 10^4^ s^−1^ previously reported for {[(dqp)Cr(L2)]_3_Er}^6+^ (*d*_Cr-Er_ = 14 Å, Figure 2c) [61].

The intrinsic energy transfer efficiency $\eta_{ETCr\to Er}$ can now be estimated from *k*_Cr_ and *k*_ET_ (ignoring the back-energy transfer):

(14) $$\begin{array}{c} \eta_{ETCr\to Er}=\frac{k_{ET}}{k_{Cr}+k_{ET}}=\;\frac{4.3\times 10^{6}}{4.0\times 10^{6}+4.3\times 10^{6}}=0.52 \end{array}$$

It reaches $\eta_{ETCr\to Er}$ = 52% efficiency, which is favorable (optimum = 50%) for boosting energy transfer upconversion (ETU) in molecular complexes [59,86].

### 2.5. Looking for Light Upconversion in [(Me_2_biim)Er(Tp)_2_]OTf Complex and in [(cyclam)Cr(biim)Er(Tp)_2_](OTf)_2_ Dyad

**Figure 13:**
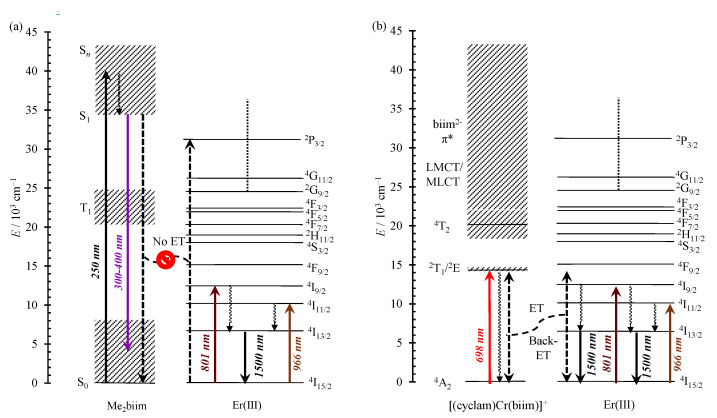
Jablonski diagrams of (**a**) [(Me_2_biim)Er(Tp)_2_]^+^ and (**b**) [(cyclam)Cr(biim)Er(Tp)_2_]^2+^ showing radiative (straight arrows) and non-radiative transitions (wavy arrows) upon NIR excitations at 250 nm, 698 nm, 801 nm and 966 nm. ET = energy transfer depicted with dashed traces.

Using high-excitation-power lasers, it is possible to excite [(Me_2_biim)Er(Tp)_2_]^+^ and [(cyclam)Cr(biim)Er(Tp)_2_]^2+^ through the Laporte-forbidden intrashell f-f transitions of the Er(III) center at 801 nm (Er(^4^I_9/2_←^4^I_15/2_)) and at 966 nm (Er(^4^I_11/2_←^4^I_15/2_)). Standard one-photon light downshifting processes result in ultimate Er(^4^I_13/2_⟶I_15/2_) emission at 1550 nm (Figure 13Figures S49 and S50). Surprisingly, upon UV excitation at 250 nm, the excited levels centered on either Tp^−^ or Me_2_biim ligands bound to Er(III) in mononuclear [(Me_2_biim)Er(Tp)_2_] only show broad ligand-centered π*⟶π emission bands (Figure S51) with no trace of Er-centered emission induced by the antenna effect (Figure 13a). This contrasts with the successful indirect UV-based sensitization of Er(III) centers observed in the [(cyclam)Cr(biim)Er(Tp)_2_]^2+^ dyad, where the doubly deprotonated bridging ligand biim^2−^ ensures ultimate sensitization of Er(^4^I_13/2_→^4^I_15/2_) *via* energy transfer (Figure 12c).

**Figure 14:**
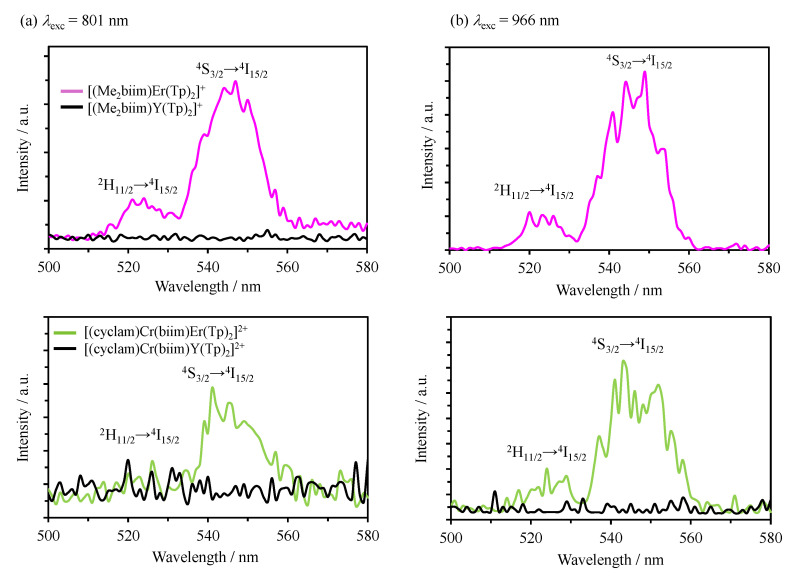
Upconversion emission spectra of [(Me_2_biim)Ln(Tp)_2_]^+^ (top) and [(cyclam)Cr(biim)Ln(Tp)_2_]^2+^ (bottom; Ln = Er or Y) in acetonitrile at room temperature (*c* = 10^−2^ M) upon laser excitation at (**a**) *λ*_exc_ = 801 nm (*P* = 1.54 W) and (**b**) *λ*_exc_ = 966 nm (*P* = 4.9 W).

**Figure 15:**
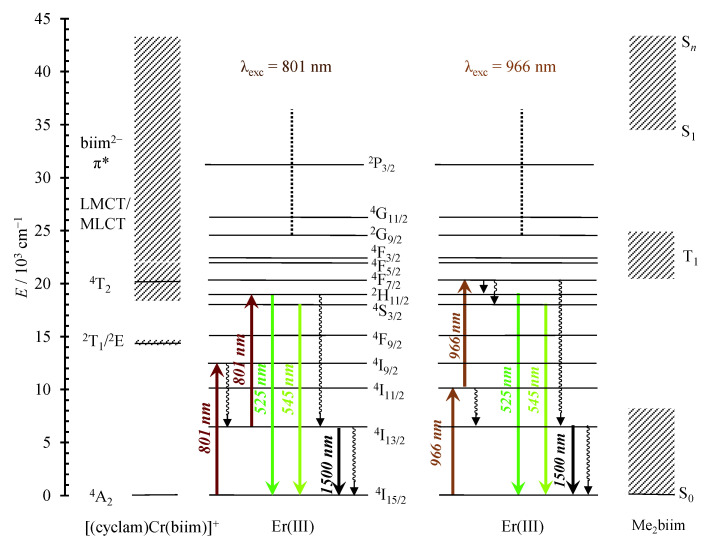
Jablonski diagrams showing the proposed two-photon erbium-centered Excited State Absorption (ESA) mechanisms operating in [(Me_2_biim)Er(Tp)_2_]^+^ and [(cyclam)Cr(biim)Er(Tp)_2_]^2+^ upon excitation at *λ*_exc_ = 801 nm (left) and *λ*_exc_ = 966 nm (right). Radiative transitions = straight arrows and non-radiative transitions = wavy arrows.

Using highly focused 801 nm and 966 nm lasers, beyond inducing the light downshifting response described above (Figure 13a), the excitation light is intense enough to induce light upconversion in solution for both [(Me_2_biim)Er(Tp)_2_]^+^ and [(cyclam)Cr(biim)Er(Tp)_2_]^2+^ at room temperature (Figure 14). The upconversion signals are, however, very weak using the 801 nm laser (close to noise level, see Figure 14a), but become slightly more intense when using the 966 nm laser (Figure 14b) because (i) our 966 nm laser can reach higher excitation power and (ii) the Er(^4^I_11/2_←^4^I_15/2_) transition absorbs more efficiently the initial photon densities than the Er(^4^I_9/2_←^4^I_15/2_) transition (see Figure S40). The upconversion emission spectra display two bands corresponding to the blue-green Er(^4^S_3/2_→^4^I_15/2_) and Er(^2^H_11/2_→^4^I_15/2_) transitions. Except for excitation of [(cyclam)Cr(biim)Er(Tp)_2_]^2+^ at 801 nm, which provided upconverted signals that were too weak to be safely recorded at low incident powers, the integrations of the upconverted emission bands upon 966 nm excitation for both complexes, [(Me_2_biim)Er(Tp)_2_]^+^ and [(cyclam)Cr(biim)Er(Tp)_2_]^2+^, are strong enough to be accessible (Figures S52 and S53). They show linear log(*I*) *vs* log(*P*) plots, with an experimental slope close to 2, which confirms the absorption of two successive photons according to the single-center Excited State Absorption (ESA) mechanism depicted in Figure 15 and previously established for closely related mononuclear Er(III) complexes in solution [59,87]. Finally, chromium-centered excitation into the Cr(^2^E,^2^T_1_←^4^A_2_) at 698 nm for [(cyclam)Cr(biim)Er(Tp)_2_]^2+^ gave only faint Er(^4^S_3/2_→^4^I_15/2_) and Er(^2^H_11/2_→^4^I_15/2_) upconverted emission bands at any temperature (Figure S54), but induced standard light-downshifting *via* efficient intramolecular Cr→Er energy transfer detailed in Section 2.4 (Figure 13b). One concludes that no reasonable Energy Transfer Upconversion (ETU) mechanism could be implemented for this specific Cr-Er dyad in solution.

To summarize, the upconversion signals induced upon excitation at *λ*_exc_ = 698, 801 or 966 nm of [(Me_2_biim)Er(Tp)_2_]^+^ and [(cyclam)Cr(biim)Er(Tp)_2_]^2+^ remain very weak (close to the detection limit of our setup), while other Er(III) complexes reported in the literature upconvert much more efficiently [86,88,89,90]. Furthermore, we found that the complex [(Me_2_biim)Er(Tp)_2_]^+^, which contains no chromium sensitizer, upconverts more efficiently than the chromium-containing [(cyclam)Cr(biim)Er(Tp)_2_]^2+^ dyad upon all the light excitations explored in this work. 

## 3. Materials and Methods

### 3.1. Solvents and Starting Materials

Chemicals were purchased from Sigma-Aldrich (Burlington, MA, United States) and Acros (Switzerland) and used without further purification unless otherwise stated. Dry reagents were either purchased as packed under inert atmosphere with acroseal and molecular sieves or distilled by appropriate procedures. Dry solvents were distilled over either calcium hydride or metallic sodium. Silica-gel plates (Merck (Germany), 60 F_254_) were used for thin-layer chromatography, and SiliaFlash^®^ (Quebec, Canada) silica gel P60 (0.04–0.063 mm) and Acros (Stabio, Switzerland) silica gel 60 (0.035–0.07 mm) were used for preparative column chromatography. The complex [Cr(phen)_2_(biim)]OTf was synthesized according to the published procedure [64]. Potassium hydrotris(1-pyrazolyl)borate (KTp) and its Ln(III) complexes Ln(Tp)_2_OTf (Ln = Y, Eu) were adapted from the literature procedure [72].

### 3.2. Spectroscopic and Analytical Measurements

^1^H and ^13^C NMR spectra were recorded at 298 K on a Bruker Avance (Karlsruhe, Germany) 400 MHz spectrometer equipped with BCU temperature control for variable temperature measurements. Chemical shifts are given in ppm with respect to TMS. Pneumatically-assisted electrospray (ESI-MS) mass spectra were recorded from ~1 × 10^−4^ M (ligands) and ~1 × 10^−3^ M (complexes) solutions on an Applied Biosystems (Foster City, CA, United States) API 150EX LC/MS System equipped with a Turbo Ionspray source. Elemental analyses were performed by K. L. Paglia from the Microchemical Laboratory of the University of Geneva. Electronic spectra in the UV-Vis region were recorded at 293 K from solutions in CH_3_CN with a Perkin-Elmer Lambda 1050 (Waltham, MA, USA) using quartz cells of 0.1 or 1.0 mm path length. Solid-state absorption spectra were recorded with a Perkin-Elmer Lambda 900 using capillaries. The emission spectra were recorded using a Fluorolog (Horiba Jobin-Yvon) instrument equipped with an iHR320 imaging spectrometer, a 450 W xenon lamp illuminator (FL-1039A/40A) and a Peltier-cooled photomultiplier tube (PMT Hamamatsu R928P, Hamamatsu, Japan). The emission spectra were corrected for the wavelength-dependent sensitivity of the PMT. Time-resolved data were collected using a digital oscilloscope (Tektronix MDO4104C, Portland, OR, United States) coupled to a water-cooled photomultiplier tube (PMT Hamamatsu R928P) or to a time-gated photomultiplier module (Hamamatsu H11526-20-NF, Hamamatsu, Japan). Pulsed excitation at 355 nm was achieved using the third harmonic of a pulsed Nd:YAG laser (Quantel Q-Smart 850, Lumibird, France) or MPL-F-355nm-100mW-DB22’005 from CNI Laser (10kHz repetition rate). Variable temperature measurements were done using a closed-cycle cryosystem (Janis, CCS-900/204N, Woburn, MA, United States) with the sample sitting in the exchange gas (helium) to achieve efficient cooling. Complexes of known corrected molecular weight were dissolved in acetonitrile to obtain ~1 mM solutions that were immobilized in 2 mm-diameter cylindrical quartz cuvettes. The cuvettes, sealed with fast-drying silver agar gel, were mounted on a metallic copper sample holder. The ultrafast time-gated experiments used a pulsed laser as an excitation light source (355 nm, CNI Laser MPL-F-355, Changchun, China). Time-correlated single-photon counting was performed with a quTAG MC (quTools), and the detector of the FluoroLog (R5509-73, Horiba Scientific, Kyoto, Japan) was used as the detector.

### 3.3. X-Ray Crystallography

Summary of crystal data intensity measurements and structure refinements for complexes [(cyclam)Cr(H_2_biim)]OTf_3_, [(cyclam)Cr(Hbiim)]OTf_2,_ [(phen)_2_Cr(biim)Ln(Tp)_2_] (Ln = Y, Eu) [(cyclam)Cr(biim)LnTp)_2_]OTf_2_ (Ln = Y, Eu, Er) and [Ln(Tp)_2_(Me_2_biim)]OTf complexes (Ln = Y, Er) are collected in Supplementary File S3 with pertinent bond lengths and bond angles. ORTEP views with numbering schemes. The crystals were mounted on Hampton cryoloops with protection oil. X-ray data collections were performed with an XtaLAB Synergy-S diffractometer (Rigaku, Japan) equipped with a hybrid pixel array “hypix arc 150” detector. The structures were solved by using dual-space methods [91]. Full-matrix least-squares refinements on F^2^ were performed with SHELXL-2020 [92]. CCDC 2550803-2550811 contains the supplementary crystallographic data for this paper. These data can be obtained free of charge from The Cambridge Crystallographic Data Center via https://www.ccdc.cam.ac.uk/structures/ (accessed on 11 May 2026).

### 3.4. Synthesis

#### 3.4.1. Synthesis of Potassium hydrotris(1-pyrazolyl)borate (KTp) [72]

In a flask, 20 g of pyrazole (291 mmol) and 4 g of KBH_4_ (74.2 mmol) were mixed, and the resulting solid was heated at 190° for 2 h under reflux, until no more hydrogen evolved. The hot mixture was precipitated in toluene under stirring, then filtered. The white solid was recrystallized in anisole. A total of 13.620 g (54.0 mmol, 73%) of KTp was obtained. ^1^H NMR (400 MHz, CD_3_CN) δ 7.54 (d, *J* = 2.1, 3H), 7.44 (d, *J* = 1.6 Hz, 3H), 6.07 (t, *J* = 1.9 Hz, 3H), and 4.71 (m, 1H).

#### 3.4.2. Synthesis of [Y(Tp)_2_OTf] [72]

In a flask, 1.290 g (2.41 mmol) of Y(OTf)_3_ was dissolved in 50 ml of dry THF. Molecular sieves were added to the solution to remove possible traces of water remaining in the solvent and in the salt. A total of 1.215 g (4.82 mmol) of KTp was added, and the solution was stirred at room temperature for 1 h. The THF was removed under reduced pressure, and the residue was dried under vacuum. Toluene was added, and the suspension was filtered to remove insoluble KOTf. The solution was evaporated under reduced pressure, then dried under vacuum at 100 °C for 30 min. To remove the last traces of toluene, the white solid was dissolved in 250 mL of Et_2_O, and the solution was evaporated under reduced pressure. The white solid was dried under vacuum overnight. A total of 1.359 g (2.05 mmol, 85%) of YTp_2_OTf was obtained. ^1^H NMR (400 MHz, CD_3_CN) δ 7.84 (d, *J* = 2.1 Hz, 6H), 7.16 (d, *J* = 2.1 Hz, 6H), 6.15 (t, *J* = 2.1 Hz, 6H), 4.74 (very br m, 2H). Anal. calc. for C_19_H_20_B_2_F_3_N_12_O_3_SY: C 34.37, H 3.04, N 25.31. Found: C 34.13, H 3.16, N 25.29.

#### 3.4.3. Synthesis of Eu(Tp)_2_OTf [72]

In a flask, 1.141 g (1.90 mmol) of Eu(OTf)_3_ was dissolved in 50 ml of dry THF. Molecular sieves were added to the solution to remove possible traces of water remaining in the solvent and in the salt. A total of 0.960 g (3.81 mmol) of KTp was added, and the solution was stirred at room temperature for 1 h. The THF was removed under reduced pressure, and the flask was dried under vacuum. Toluene was added, and the suspension was filtered to remove insoluble KOTf. The solution was evaporated under reduced pressure, then dried under vacuum at 100 °C for 30 min. To remove the last traces of toluene, the white solid was dissolved in 250 mL of Et_2_O, and the solution was evaporated under reduced pressure. The white solid was dried under vacuum overnight. A total of 1.003 g (1.38 mmol, 72%) of EuTp_2_OTf was obtained. ^1^H NMR (400 MHz, CD_3_CN) δ 13.93 (s, 6H), 3.04 (d, *J* = 2.1 Hz, 6H), 0.46 (s, 6H), −1.06–−2.19 (very br m, 2H). Anal. calc. for C_19_H_20_B_2_F_3_N_12_O_3_SEu: C 31.39, H 2.77, N 23.12. Found: C 31.37, H 2.72, N 23.21.

#### 3.4.4. Synthesis of [(Me_2_biim)Y(Tp)_2_]OTf

In a flask, 25 mg (0.15 mmol) of Me_2_biim and 100 mg (0.15 mmol) of YTp_2_OTf were dissolved in dry MeCN. The solution was evaporated under reduced pressure, yielding 111 mg (0.13 mmol, 89%) of [(Me_2_biim)Y(Tp)_2_]OTf as a white powder. Vapor diffusion of pentane into a THF solution of [(Me_2_biim)Y(Tp)_2_]OTf led to the formation of block-shaped crystals suitable for XRD. Anal. calc. for C_27_H_30_B_2_F_3_N_16_O_3_SY: C 39.25, H 3.66, and N 27.12. Found: C 38.76, H 3.48, N 26.78. m/z (ESI-HRMS): calcd. for [Y(Tp)_2_(Me_2_biim)]^+^ (C_26_H_30_B_2_N_16_Y^+^): 677.209, found: 677.208.

#### 3.4.5. Synthesis of [(Me_2_biim)Er(Tp)_2_]OTf

In a flask, 26 mg (0.16 mmol) of Me_2_biim and 120 mg (0.16 mmol) of ErTp_2_OTf were dissolved in dry MeCN. The solution was stirred for 5 min at room temperature. The solution was then evaporated under reduced pressure, yielding 124 mg (0.14 mmol, 85%) of [(Me_2_biim)Er(Tp)_2_]OTf as a white powder. Vapor diffusion of pentane into a THF solution of [(Me_2_biim)Er(Tp)_2_]OTf led to the formation of block-shaped crystals suitable for XRD. Anal. calc. for C_27_H_30_B_2_F_3_N_16_O_3_SEr·1.2H_2_O: C 35.01, H 3.53, and N 24.20. Found: C 34.84, H 3.29, and N 23.97. m/z (ESI-HRMS): calcd. for [Er(Tp)_2_(Me2biim)]^+^ (C_26_H_30_B_2_N_16_Er^+^): 754.235, found: 754.235.

#### 3.4.6. Synthesis of [Cr(cyclam)Cl_2_]Cl [78,79]

A total of 1.00 g (5.00 mmol) of cyclam and 1.50 g (5.63 mmol) of CrCl_3_·6H_2_O were dissolved in DMF (30mL). The solution was refluxed for 20 min. The solution was cooled and filtered, and the purple precipitate was washed with 20 mL of acetone and dried. A total of 1.57 g (4.37 mmol, 88%) of cis-[Cr(cyclam)Cl_2_]Cl was obtained as a purple powder.

#### 3.4.7. Synthesis of [Cr(cyclam)OTf_2_]OTf [93]

A total of 1.568 g (4.37 mmol) of cis-[Cr(cyclam)Cl_2_]Cl was added to 2 mL (22.8 mmol) of trifluoromethanesulfonic acid in a Schlenk tube connected to an N_2_ flux and a trap bubbler with a Na_2_CO_3_ aqueous solution to quench gaseous HCl. The solution was stirred for 1 h at room temperature, then 1 h at 100 °C. The mixture was cooled to 5 °C and was carefully poured into 40 mL of Et_2_O. A pink precipitate started to form with a little bit of scratching to help the precipitation process. The solid was filtered and washed twice with Et_2_O (40 mL). A total of 2.413 g (3.45 mmol, 79%) of cis-[Cr(cyclam)OTf_2_]OTf was obtained as a pink powder.

#### 3.4.8. Synthesis of [(cyclam)Cr(H_2_biim)]OTf_3_

A total of 500 mg (0.715 mmol) of [Cr(cyclam)OTf_2_]OTf and 100 mg (7.45 mmol) of H_2_biim were added in a vial with 10 mL of MeCN. The vial was heated for 3 h at 120° under microwave irradiation; the dark orange mixture was filtered to remove excess H_2_biim. TEA (1 mL) was then added, and an orange precipitate started to form immediately. The precipitate was filtered, and the orange powder was redissolved in 5mL MeOH with 0.1 M of HOTf. The solution was filtered to remove any insoluble solid, then precipitated by adding Et_2_O into the orange solution. A total of 0.338g (0.405 mmol, 57%) of [(cyclam)Cr(H_2_biim)]OTf_3_ was obtained as an orange powder. Anal. calc. for C_19_H_30_CrF_9_N_8_O_9_S_3_: C 27.37, H 3.63, and N 13.44. Found: C 27.13, H 3.68, N 13.44. *m*/*z* (ESI-HRMS,): calcd. for [Cr(cyclam)(Hbiim)]OTf^+^ (C_17_H_29_CrF_3_N_8_O_3_S^+^): 534.144, found: 534.144; calcd. for [Cr(cyclam)(Hbiim)]^2+^ (C_16_H_29_CrN_8_^2+^): 192.595, found: 192.594; calcd. for [Cr(cyclam)(biim)]^+^ (C_16_H_28_CrN_8_^+^): 384.183, found: 384.183.

#### 3.4.9. Synthesis of [(cyclam)Cr(Hbiim)]OTf_2_

A total of 100 mg (0.12 mmol) of [Cr(cyclam)(H_2_biim)]OTf_3_ was dissolved in 1 mL of H_2_O. A total of 120 µL of aqueous NaOH 1 M (0.12 mmol) was added to the solution. The solution was evaporated completely under reduced pressure. The orange solid was dissolved in 2 mL of methanol, and the solution was recrystallized by slow diffusion of Et_2_O into the solution. A total of 59 mg (0.086 mmol, 72%) of orange crystals was collected after filtration. (ESI-HRMS): calcd. for [Cr(cyclam)(Hbiim)]OTf^+^ (C_17_H_29_CrF_3_N_8_O_3_S^+^): 534.144, found: 534.144; calcd. for [Cr(cyclam)(Hbiim)]^2+^ (C_16_H_29_CrN_8_^2+^): 192.595, found: 192.594; calcd. for [Cr(cyclam)(biim)]^+^ (C_16_H_28_CrN_8_^+^): 384.183, found: 384.183.

#### 3.4.10. Synthesis of [(cyclam)Cr(biim)]OTf

A total of 500 mg (0.600 mmol) of [(cyclam)Cr(H_2_biim)]OTf_3_ was dissolved in 10 mL of MeCN, 1.0 mL (7.2 mmol) of triethylamine was added, and an orange precipitate immediately formed. The suspension was filtered, and the solid was washed with 10 mL MeCN and 10 mL of Et_2_O. A total of 305 mg (0.572 mmol, 95%) of [(cyclam)Cr(biim)]OTf was obtained as an orange powder. Anal. calc. for C_17_H_28_CrF_3_N_8_O_3_S·1.15H_2_O: C 36.84, H 5.51, N 20.22. Found: C 36.95, H 5.25, N 19.96. *m*/*z* (ESI-HRMS): calcd. for [Cr(cyclam)(Hbiim)]OTf^+^ (C_17_H_29_CrF_3_N_8_O_3_S^+^): 534.144, found: 534.144; calcd. for [Cr(cyclam)(Hbiim)]^2+^ (C_16_H_29_CrN_8_^2+^): 192.595, found: 192.594; calcd. for [Cr(cyclam)(biim)]^+^ (C_16_H_28_CrN_8_^+^): 384.183, found: 384.183.

#### 3.4.11. Synthesis of [(cyclam)Cr(biim)Y(Tp)_2_]OTf_2_

A total of 60 mg (0.090 mmol) of [Y(Tp)_2_OTf] was dissolved in 5 mL of MeCN, and 52 mg (0.098 mmol) of [Cr(cyclam)(biim)]OTf was added to the solution. The solution was heated to help the solution solubilize. The solution was filtered to remove any excess insoluble [Cr(cyclam)(biim)]OTf. A total of 50 mL of Et_2_O was added to the clear orange solution. A precipitate started to appear after a few minutes and was filtered after 2 h. The solid was washed with Et_2_O and dried. A total of 101 mg (0.084 mmol, 93%) of [(cyclam)Cr(biim)Y(Tp)_2_]OTf_2_ was obtained as an orange powder. Anal. calc. for C_36_H_48_B_2_CrF_6_N_20_O_6_S_2_Y·3.15H_2_O: C 34.47, H 4.36, N 22.33. Found: C 34.56, H 4.27, N 21.95. *m*/*z* (ESI-HRMS): calcd. for [(cyclam)Cr(biim)Y(Tp)_2_]OTf^+^ (C_35_H_48_B_2_CrF_3_N_20_O_3_SY^+^): 1048.253, found: 1048.253.

#### 3.4.12. Synthesis of [(cyclam)Cr(biim)Eu(Tp)_2_]OTf_2_

A total of 70 mg (0.096 mmol) of [Eu(Tp)_2_OTf] was dissolved in 5 mL of MeCN, and 50 mg (0.094 mmol) of [Cr(cyclam)(biim)]OTf was added to the solution. The solution was heated to help the solution to solubilize. The solution was filtered to remove any excess of insoluble [Cr(cyclam)(biim)]OTf. A total of 50 mL of Et_2_O was added to the clear orange solution. A precipitate started to appear after a few minutes and was filtered after 2 h. The solid was washed with Et_2_O and dried. A total of 93 mg (0.074 mmol, 79%) of [(cyclam)Cr(biim)Eu(Tp)_2_]OTf_2_ was obtained as an orange powder. Anal. calc. for C_36_H_48_B_2_CrF_6_N_20_O_6_S_2_Eu·3.2H_2_O: C 32.80, H 4.16, N 21.25. Found: C 32.84, H 4.09, N 21.19. *m*/*z* (ESI-HRMS): calcd. for [(cyclam)Cr(biim)Eu(Tp)_2_]OTf^+^ (C_35_H_48_B_2_CrF_3_N_20_O_3_SEu^+^): 1112.268, found: 1112.267.

#### 3.4.13. Synthesis of [(cyclam)Cr(biim)Er(Tp)_2_]OTf_2_

A total of 30 mg (0.056 mmol) of [Er(Tp)_2_OTf] was dissolved in 10 mL of MeCN, and 42 mg (0.057 mmol) of [Cr(cyclam)(biim)]OTf was added to the solution. The mixture was stirred until the powder completely solubilized. The orange solution was evaporated under reduced pressure. A total of 70 mg (0.055 mmol, 98%) of [(cyclam)Cr(biim)Er(Tp)_2_]OTf_2_ was obtained as an orange powder. Anal. calc. for C_36_H_48_B_2_CrF_6_N_20_O_6_S_2_Er·2.3H_2_O: C 32.82, H 4.02, N 21.27. Found: C 32.77, H 3.91, N 21.16. *m*/*z* (ESI-HRMS): calcd. for [(cyclam)Cr(biim)Er(Tp)_2_]OTf^+^ (C_35_H_48_B_2_CrF_3_N_20_O_3_SEr^+^): 1126.282, found: 1126.286.

## 4. Conclusions

The doubly deprotonated complex-as-ligand [(phen)_2_Cr(biim)]^+^ binds to Ln(III) triflate (Ln = Lu or La) to give heterometallic d-f assemblies {Ln[(biim)Cr(phen)_2_]*_n_*}^(*n*+3)+^ with stoichiometric ratios 1 ≤ *n* ≤ 4. Reaching such high nuclearity for the LnCr_4_ complex, despite the considerable charge repulsion, is a clear indication that the biimidazolate ligand is a valuable and attractive bridging ligand for connecting Cr(III) cations to trivalent lanthanides. However, the successive anti-cooperative association steps limit the thermodynamic formation constants so that intricate mixtures of different LnCr*_n_* species exist in solution at millimolar concentrations, which prevents easy access to individual and well-defined assemblies.

Reaction of [(phen)_2_Cr(biim)]^+^ complex-as-ligand with the coordinately unsaturated lanthanide complex [Ln(Tp)_2_]^+^ in acetonitrile led to the selective formation of stable and fully characterized heterometallic [(phen)_2_Cr(biim)Ln(Tp)_2_]^2+^ (Ln = Y, Eu) dyads. The short intermetallic distance (5.9 Å) ensured by the compact biimidazolate bridge is appropriate for energy transfer between the metallic centers following light excitation. However, the visible tail of the biim^2−^→phen LLCT band efficiently quenches the doublet Cr(^2^E,^2^T1) excited state at room temperature, thus preventing its use as a donor for ultimate lanthanide sensitization. 

Replacement of the two polyaromatic didentate phen ligands with an aliphatic tetradentate cyclam leads to closely related [(cyclam)Cr(biim)Ln(Tp)_2_]^2+^ dyads in solution (Ln = Y, Eu, Er). The targeted removal of low-energy LLCT bands makes Cr(^2^E,^2^T_1_) excited centers of the latter dyads emissive at room temperature following UV excitation. Moreover, a remarkably efficient intramolecular Cr→Er energy transfer (*k*_ET_ = 4.3 × 10^6^ s^−1^, $\eta_{ETCr\to Er}$ = 52%) is responsible for infrared Er(^4^I_13/2_→^4^I_15/2_) emission at 1550 nm following visible Cr(^4^T_2_←^4^A_2_) excitation at 500 nm, or Cr(^2^T_1_,^2^E←^4^A_2_) excitation at 698 nm.

Focusing laser excitation beams at 801 nm (Er(^4^I_9/2_←^4^I_15/2_)), or at 966 nm (Er(^4^I_11/2_←^4^I_15/2_)), induces weak upconverted signals at 525 nm (Er(^2^H_11/2_→^4^I_15/2_)) and 545 nm (Er(^4^S_3/2_→^4^I_15/2_)) in the [(cyclam)Cr(biim)Er(Tp)_2_]^2+^ dyad and its mononuclear model complex [(Me_2_biim)Er(Tp)_2_]^+^ (ESA mechanism). However, the Cr(III) sensitizer does not contribute to the light upconversion. Attempts to directly excite Cr(^2^E,^2^T_1_←^4^A_2_) excitation at 698 nm for inducing an alternative energy transfer upconversion (ETU) mechanism failed. Indeed, the upconversion quantum yield for the ETU mechanism $\Phi^{up}\left(ETU\right)$ for a sensitizer (Cr)–acceptor (Er) dyad can be estimated with Equation (15) [86]:

(15) $$\begin{array}{c} {\Phi^{up}\left(ETU\right)}_{Cr-Er}\approx \left[\left(\frac{\lambda_{p}}{hc}\right){\times \sigma }_{Cr}^{0\to 1}\times P\right]\eta_{ET\;1}\times \eta_{ET\;2}\times \tau_{Er}^{1}{\times \Phi }_{Er}^{rad\left(2\to 0\right)} \end{array}$$

*λ*_p_ is the excitation wavelength with intensity *P*, and h and c are the Planck constant and the speed of light; $\sigma_{Cr}^{0\to 1}$ is the absorption cross-section of the sensitizer, which is directly proportional to the absorption coefficient $\varepsilon_{Cr}^{0\to 1}$ of the Cr(^2^E,^2^T_1_←^4^A_2_) transition; $\tau_{Er}^{1}$ is the lifetime of the first excited state of the acceptor working as relay, $\Phi_{Er}^{rad\left(2\to 0\right)}$ is the intrinsic radiative quantum yield of the transition 2→0 of the doubly excited acceptor, $\eta_{ET\;1}$ and $\eta_{ET\;2}$ are the intrinsic energy transfer efficiency from the excited sensitizer to the first (0→1) and second transition of the acceptor (1→2), respectively. Using a Cr(III)-based sensitizer for implementing ETU on the Er(III) partner in [(cyclam)Cr(biim)Er(Tp)_2_]^2+^ provides a too small absorption cross-section $\sigma_{S}^{0\to 1}$ (Figure 10, ε_Cr_ = 0.8 M^−1^·cm^−1^ at 698 nm) for overcoming the deleterious short $\tau_{Er}^{1}$ (within the microsecond domains [87]) and the faint intrinsic quantum yields $\Phi_{Er}^{rad\left(2\to 0\right)}$ (within the 10^−6^–10^−5^ range for molecular erbium complexes [86]). Optimizing the energy transfer rate constants will not solve this issue because the energy transfer efficiency $n_{ET\;1}$ = 0.52 operating in [(cyclam)Cr(biim)Cr(Tp)_2_]^2+^ is already favorable and $n_{ET\;2}$ can never exceed 1. 

## Figures and Tables

**Table 1:** Association constants represented as $log\left(\beta_{1,n}^{Ln,Cr}\right)$ extracted from the titration of [(phen)_2_Cr(biim)]^+^ with Ln(OTf)_3_ (Ln = La, Lu) or Zn(OTf)_2_ in CH_3_CN (293 K).

Titration	Lu/Cr	La/Cr	Zn/Cr
$log\left(\beta_{1,1}^{Ln,Cr}\right)$	9.01(3)	8.16(5)	8.4(2)
$log\left(\beta_{1,2}^{Ln,Cr}\right)$	14.8(3)	16.7(1)	14.5(6)
$log\left(\beta_{1,3}^{Ln,Cr}\right)$	21.1(5)	24.2(3)	20.1(8)
$log\left(\beta_{1,4}^{Ln,Cr}\right)$	26.1(6)	28.8(3)	-
Δ*G*_M,Cr_/kJ·mol^−1^	−38(2)	−42(2)	−38(2)
Δ*E*_Cr,Cr/_kJ·mol^−1^	3(2)	2(2)	4(2)
Ionic radius of the metal/Å	1.12	1.30	0.88
*Z*^2^/*R* of the metal/Å^−1^	8.0	6.9	4.5

**Table 2:** Radiative (*τ*_rad_ = 1/*k*_rad_, Equation (12)) and total (*τ*_tot_ = 1/*k*_tot_) emission lifetimes, and intrinsic emission quantum yield Φ_intrinsic_ (Equation (13)) of the Cr(^2^E) excited levels in [(cyclam)Cr(H_2_biim)]^+^, [Cr(cyclam)(Hbiim)]^2+^ and [(cyclam)Cr(biim)Ln(Tp)_2_]^2+^ (Ln = Y, Eu, Er) complexes in CH_3_CN at room temperature upon excitation at *λ*_exc_ = 480 nm, and recording at *λ*_em_ = 700 nm (*λ*_em_ = 690 nm for [Cr(cyclam)(H_2_biim)]^3+^).

Complex	*τ*_rad_/ms	*τ*_tot_/μs	*Φ*_intrinsic_
[Cr(cyclam)(H_2_biim)]^3+^	7.3(4)	1.13(6)	1.6(1) × 10^−4^
[Cr(cyclam)(Hbiim)]^2+^	7.4(4)	0.42(2)	5.7(4) × 10^−5^
[(cyclam)Cr(biim)Y(Tp)_2_]^2+^	5.7(3)	0.25(2)	4.4(3) × 10^−5^
[(cyclam)Cr(biim)Eu(Tp)_2_]^2+^	6.5(3)	0.23(2)	3.5(3) × 10^−5^
[(cyclam)Cr(biim)Er(Tp)_2_]^2+^	4.1(7)	0.101(5); 0.51(3)	2.5(7) × 10^−5^

## Data Availability

Supporting information contains the following information: Experimental section, NMR spectra, crystal structures, thermodynamic data, and photophysical data for the synthesized compounds. CCDC 2550803-2550811 contain the supplementary crystallographic data for this paper. These data can be obtained free of charge from The Cambridge Crystallographic Data Centre via www.ccdc.cam.ac.uk/data_request/cif (accessed on 11 May 2026).

## Supplementary Materials

The following supporting information can be downloaded at: https://www.mdpi.com/article/10.3390/molecules31122016/s1, Tables S1–S18 and Figures S1–S54 collect thermodynamic analysis, photophysical data (absorption and emission spectra), ESI-MS data, NMR data, together with six supplementary files. Supplementary File S1. Symmetry number method; Supplementary File S2; Spectrophotometric titrations of [(phen)_2_Cr(biim)]OTf with Ln(Tp)2OTf; Supplementary File S3. Crystal structures; Supplementary File S4. Characterization of [(Me_2_biim)Ln(Tp)_2_]^+^ (Ln = Y, Er) in solution; Supplementary File S5. Synthesis of Me_4_cyclam and its Cr(III) complexes; Supplementary File S6. Kinetic modeling of the excited state dynamics of complex [(cyclam)Cr(biim)Er(Tp)_2_].

## References

[1] Tanabe Y., Sugano S. (1954). On the Absorption Spectra of Complex Ions 2. J. Phys. Soc. Jpn..

[2] Brik M.G., Srivastava A.M. (2013). Systematic Analysis of the Spectroscopic Characteristics of 3d Ions in a Free State and Some Cubic Crystals. Opt. Mater..

[3] Zhou Q., Dolgov L., Srivastava A.M., Zhou L., Wang Z.L., Shi J.X., Dramicanin M.D., Brik M.G., Wu M.M. (2018). Mn(2+) and Mn(4+) Red Phosphors: Synthesis, Luminescence and Applications in WLEDs. A Review. J. Mater. Chem. C.

[4] Ferguson J. (1970). Spectroscopy of 3d Complexes. Prog. Inorg. Chem..

[5] Adachi S. (2021). Spectroscopy of Cr3+ Activator: Tanabe-Sugano Diagram and Racah Parameter Analysis. J. Lumin..

[6] Adachi S. (2024). Practical Consideration on Racah Parameter and Tanabe- Sugano Diagram Analyses for Mn(4+)and Cr(3+)-Activated Phosphors. J. Lumin..

[7] Frost J.M., Harriman K.L.M., Murugesu M. (2016). The Rise of 3-d Single-Ion Magnets in Molecular Magnetism: Towards Materials from Molecules?. Chem. Sci..

[8] Winpenny R.E.P. (2002). Serendipitous Assembly of Polynuclear Cage Compounds. J. Chem. Soc. Dalton.

[9] Timco G.A., McInnes E.J.L., Pritchard R.G., Tuna F., Winpenny R.E.P. (2008). Heterometallic Rings Made From Chromium Stick Together Easily. Angew. Chem. Int. Ed..

[10] Geue N., Kumar D., Ham J., Huang S.P., Timco G.A., Burton N.A., Winpenny R.E.P., Anggara K., Barran P.E. (2025). Self-Assembly, Rearrangement, and Disassembly of {Cr6} Horseshoe Oligomers. Angew. Chem. Int. Ed..

[11] Langley S.K., Wielechowski D.P., Vieru V., Chilton N.F., Moubaraki B., Chibotaru L.F., Murray K.S. (2014). Modulation of Slow Magnetic Relaxation by Tuning Magnetic Exchange in {CrDy} Single Molecule Magnets. Chem. Sci..

[12] Langley S.K., Wielechowski D.P., Chilton N.F., Moubaraki B., Murray K.S. (2015). A Family of {CrLn} Butterfly Complexes: Effect of the Lanthanide Ion on the Single-Molecule Magnet Properties. Inorg. Chem..

[13] Pedersen K.S., Sorensen M.A., Bendix J. (2015). Fluoride-Coordination Chemistry in Molecular and Low-Dimensional Magnetism. Coord. Chem. Rev..

[14] Langley S.K., Wielechowski D.P., Moubaraki B., Murray K.S. (2016). Enhancing the Magnetic Blocking Temperature and Magnetic Coercity of {Cr(III)_2_Ln(III)_2_} Single-Molecule Magnets via Bridging Ligand Modification. Chem. Commun..

[15] Shukla P., Das S., Bag P., Dey A. (2023). Magnetic Materials Based on Heterometallic Cr(II)/(III)-Ln(III) Complexes. Inorg. Chem. Front..

[16] Swain A., Whyatt Y.L., Wielechowski D., Muthu S., Benjamin S.L., Murray K.S., Rajaraman G., Langley S.K. (2025). Enhancing Blocking Temperatures in {CrDy} Butterfly SMMs: Deciphering the Role of Exchange Interactions and Developing Magneto-structural Maps. Inorg. Chem. Front..

[17] Liu S.J., Xie X.R., Zheng T.F., Bao J., Liao J.S., Chen J.L., Wen H.R. (2015). Three-Dimensional Two-Fold Interpenetrated Cr-Gd Heterometallic Framework as an Attractive Cryogenic Magnetorefrigerant. CrystEngComm.

[18] Cui C., Cao J.-P., Luo X.-M., Lin Q.-F., Xu Y. (2018). Two Pairs of Chiral “Tower-Like” Ln4Cr4 (Ln = Gd, Dy) Clusters: Syntheses, Structure, and Magneticaloric Effect. Chem. Eur. J..

[19] Cabrosi D., Ortiz J.H., Cruz C., Paredes-García V., Alborés P. (2025). A {CrLn} Complex with Exchange Coupled {Cr_2_} Units: Structural Description and Magnetic Study. Chem. Eur. J..

[20] Campanella A.J., Nguyen M.T., Zhang J., Ngendahimana T., Antholine W.E., Eaton G.R., Eaton S.S., Glezakou V.A., Zadrozny J.M. (2021). Ligand Control of Low-Frequency Electron Paramagnetic Resonance Linewidth in Cr(III) Complexes. Dalton Trans..

[21] Lenz S., Bamberger H., Hallmen P.P., Thiebes Y., Otto S., Heinze K., van Slageren J. (2019). Chromium(III)-Based Potential Molecular Quantum Bits with Long Coherence Times. Phys. Chem. Chem. Phys..

[22] Helm L., Merbach A.E. (2005). Inorganic and Bioinorganic Solvent Exchange Mechanisms. Chem. Rev..

[23] Richens D.T. (2005). Ligand Substitution Reactions at Inorganic Centers. Chem. Rev..

[24] Barker K.D., Barnett K.A., Connel S.M., Glaeser J.W., Wallace A.J., Wildsmith J., Herbert B.J., Wheeler J.F., Kane-Maguire N.A.P. (2001). Synthesis and Characterization of Heteroleptic [Cr(diimine)_3_]^3+^ Complexes. Inorg. Chim. Acta.

[25] Donnay E.G., Schaeper J.P., Brooksbank R.D., Fox J.L., Potts R.G., Davidsdon R.M., Wheeler J.F., Kane-Maguire N.A.P. (2007). Synthesis and Characterization of Tris(heteroleptic)diimine Complexes of Chromium(III). Inorg. Chim. Acta.

[26] Jimenez J.-R., Doistau B., Poncet M., Piguet C. (2021). Heteroleptic Trivalent Chromium in Coordination Chemistry: Novel Building Blocks for Addressing Old Challenges in Multimetallic Luminescent Complexes. Coord. Chem. Rev..

[27] Kirk A.D. (1999). Photochemistry and Photophysics of Chromium(III) Complexes. Chem. Rev..

[28] Kane-Maguire N.A.P. (2007). Photochemistry and Photophysics of Coordination Compounds: Chromium. Top. Curr. Chem..

[29] Burgin T.H., Glaser F., Wenger O.S. (2022). Shedding Light on the Oxidizing Properties of Spin-Flip Excited States in a Cr(III) Polypyridine Complex and Their Use in Photoredox Catalysis. J. Am. Chem. Soc..

[30] Yang G.J., Shillito G.E., Seeber P., Wenger O.S., Kupfer S. (2025). Unraveling the Photoredox Chemistry of a Molecular Ruby. Chem. Sci..

[31] Kitzmann W.R., Moll J., Heinze K. (2022). Spin-Flip Luminescence. Photochem. Photobiol. Sci..

[32] Forster L.S. (1990). The Photophysics of Chromium(III) Complexes. Chem. Rev..

[33] Hauser A., Reber C. (2017). Spectroscopy and Chemical Bonding in Transition Metal Complexes. Structure and Bonding.

[34] Wenger O.S. (2018). Photoactive Complexes with Earth-Abundant Metals. J. Am. Chem. Soc..

[35] Förster C., Heinze K. (2020). Photophysics and Photochemistry with Earth-Abundant Metals—Fundamentals and Concepts. Chem. Soc. Rev..

[36] Kitzmann W.R., Heinze K. (2023). Charge-Transfer and Spin-Flip States: Thriving as Complements. Angew. Chem. Int. Ed..

[37] Sinha N., Yaltseva P., Wenger O.S. (2023). The Nephelauxetic Effect Becomes an Important Design Factor for Photoactive First-Row Transition Metal Complexes. Angew. Chem. Int. Ed..

[38] Ma C.-G., Brik M.G., Liu D.-X., Feng B., Tian Y., Suchocki A. (2016). Energy Level Schemes of f*^N^* Electronic Configurations for the Di-, Tri-, and Tetravalent Lanthanides and Actinides in a Free State. J. Lumin..

[39] Bovero E., Cavalli E., Jaque D., Solé J.G., Speghini A., Bettinelli M. (2005). Cr(3+)→Nd(3+) Energy Transfer in the YAl_3_(BO_3_)4 Nonlinear Laser Crystal. J. Appl. Phys..

[40] Xu J., Ueda J., Tanabe S. (2016). Novel Persistent Phosphors of Lanthanide-Chromium Co-doped Yttrium Aluminum Gallium Garnet: Design Concept with Vacuum Referred Binding Energy Diagram. J. Mater. Chem. C.

[41] Li Y.J., Cheng L.X., Liu M., Gong W.P., Zhao Z.T., Song J.X., Tang W.D., Cao R.P. (2020). Site-Related Broadband Sensitization in La_3_Ga_5.5_Nb_0.5_O_14_: Cr(3+), Ln(3+) (Ln = Yb, Er) phosphors. Spectrochim. Acta A.

[42] Wang X.H., Hao P.C., Gao J.L., Qiao X., Liu Y.Y., Wang Z.Z., Wang J., Liu B., Zhang J., Wang J.Y. (2026). Breaking Spin-Forbidden Transition by an Antisite Defect Strategy for Enhancing Far-Red Emission of Cr(3+)-Activated Phosphors. Inorg. Chem..

[43] Dong L.P., Zhang L., Jia Y.C., Xu Y.H., Yin S.W., You H.P. (2021). Realizing Broadband Spectral Conversion in Novel Ce(3+), Cr(3+), Ln(3+) (Ln = Yb, Nd, Er) Tridoped Near-Infrared Phosphors via Multiple Energy Transfers. Ceram. Int..

[44] Piotrowski W.M., Maciejewska K., Dalipi L., Fond B., Marciniak L. (2022). Cr(3+) Ions as an Efficient Antenna for the Sensitization and Brightness Enhancement of Nd(3+), Er(3+)-Based Ratiometric Thermometer in GdScO Perovskite Lattice. J. Alloy Compd..

[45] Gan W.J., Cao L.Y., Gu S.M., Lian H.W., Xia Z.G., Wang J. (2023). Broad-Band Sensitization in Cr(3+)-Er(3+) Co-Doped Cs_2_AgInCl_6_ Double Perovskites with 1.5 μm Near-Infrared Emission. Chem. Mater..

[46] Liu S.Q., Guo Y., Song Z., Peng D.F., Liu Q.L., Wang F. (2025). Bright Chromium-Sensitized Lanthanide NIR-II Mechanoluminescence in a Piezoelectric Oxide. Adv. Mater..

[47] Ming J., Xie Z., Wu J.X., Zhang F. (2025). Synthesis of Transition Metal-Sensitized Lanthanide Near-Infrared Luminescent Nanoparticles. Nat. Protoc..

[48] Heer S., Wermuth M., Krämer K., Ehrentraut D., Güdel H.U. (2001). Up-Conversion Excitation of Sharp Cr(III) ^2^E emission in YGG and YAG codoped with Cr(III) and Yb(III). J. Lumin..

[49] Guo Y.A., Zhao L.J., Fu Y.T., Yu H. (2019). Tailoring Up-Conversion Luminescence for Single Band Located in First Biological Windows and Optical Thermometry of Yb(3+)/Ln(3+) (Ln = Er, Tm) doped oxyfluoride ceramics via Cr(3+) doping. J. Lumin..

[50] Song Z., Tanner P.A., Liu Q.L. (2024). Host Dependency of Boundary between Strong and Weak Crystal Field Strength of Cr(III) Luminescence. J. Phys. Chem. Lett..

[51] Ishii T., Tsuboi S., Sakane G., Yamashita M., Breedlove B.K. (2009). Universal Spectrochemical Series of Six-Coordinate Octahedral Metal Complexes for Modifying the Ligand Field Splitting. Dalton Trans..

[52] Liu S.Q., Li L.P., Chen B., Liu Q.L., Wang F. (2026). Chromium-Activated Phosphors: From Theory to Applications. Chem. Soc. Rev..

[53] Brayshaw P.A., Bünzli J.-C.G., Froidevaux P., Harrowfield J.M., Kim Y., Sobolev A.N. (1995). Synthetic, Structural and Spectroscopic Studies on Solids Containing Tris(dipicolinato) Rare Earth Anions and Transition or Main Group Metal Cations. Inorg. Chem..

[54] Kalmbach J., Wang C., You Y., Förster C., Schubert H., Heinze K., Resch-Genger U., Seitz M. (2020). Near-IR to Near-IR Upconversion Luminescence in Molecular Chromium Ytterbium Salts. Angew. Chem. Int. Ed..

[55] Lazarides T., Davies G.M., Adams H., Sabatini C., Barigelletti F., Barbieri A., Pope S.J.A., Faulkner S., Ward M.D. (2007). Ligand-Field Excited States of Hexacyanochromate and Hexacyanocobaltate as Sensitisers for Near-Infrared Luminescence from Nd(III) and Yb(III) in Cyanide-Bridged d–f Assemblies. Photochem. Photobiol. Sci..

[56] Aboshyan-Sorgho L., Cantuel M., Petoud S., Hauser A., Piguet C. (2012). Optical Sensitization and Upconversion in Discrete Polynuclear Chromium-Lanthanide Complexes. Coord. Chem. Rev..

[57] Sanada T., Suzuki T., Yoshida T., Kaizaki S. (1998). Heterodinuclear Complexes Containing d- and f-Block Elements: Synthesis, Structural Characterization and Metal-Metal Interactions of Novel Chromium(III)-Lanthanide(III) Compounds Bridged by Oxalate. Inorg. Chem..

[58] Zare D., Suffren Y., Guénée L., Eliseeva S.V., Nozary H., Aboshyan-Sorgho L., Petoud S., Hauser A., Piguet C. (2015). Smaller than a Nanoparticle with the Design of Discrete Polynuclear Molecular Complexes Displaying Near-Infrared to Visible Upconversion. Dalton Trans..

[59] Suffren Y., Zare D., Eliseeva S.V., Guénée L., Nozary H., Lathion T., Aboshyan-Sorgho L., Petoud S., Hauser A., Piguet C. (2013). Near-Infrared to Visible Light-Upconversion in Molecules: From Dream to Reality. J. Phys. Chem. C.

[60] Subhan M.A., Nakata H., Suzuki T., Choi J.-H., Kaizaki S. (2003). Simultaneous observation of low temperature 4f-4f and 3d-3d emission spectra in a series of Cr(III)oxLn(III) assembly. J. Lumin..

[61] Poncet M., Besnard C., Jimenez J.R., Piguet C. (2024). Maximizing Nanoscale Downshifting Energy Transfer in a Metallosupramolecular Cr(III)-Er(III) Assembly. Inorg. Chem..

[62] Lazarides T., Sykes D., Faulkner S., Barbieri A., Ward M.D. (2008). On the Mechanism of d-f Energy Transfer in Ru(II)/Ln(III) and Os(II)/Ln(III) Dyads: Dexter-Type Energy Transfer over a Distance of 20Å. Chem. Eur. J..

[63] Ward M.D. (2010). Mechanisms of Sensitization of Lanthanide(III)-Based Luminescence in Transition Metal/Lanthanide and Anthracene/Lanthanide Dyads. Coord. Chem. Rev..

[64] Chong J., Benchohra A., Besnard C., Guenee L., Rosspeintner A., Cruz C.M., Jimenez J.R., Piguet C. (2024). Taming 2,2′-Biimidazole Ligands in Trivalent Chromium Complexes. Dalton Trans..

[65] Gampp H., Maeder M., Meyer C.J., Zuberbuehler A.D. (1985). Calculation of Equilibrium Constants from Multiwavelength Spectroscopic Data. III. Model-free Analysis of Spectrophotometric and ESR titrations. Talanta.

[66] Gampp H., Maeder M., Meyer C.J., Zuberbuehler A.D. (1986). Calculation of Equilibrium Constants from Multiwavelength Spectroscopic Data—IV. Model-free Least-Squares Refinement by Use of Evolving Factor Analysis. Talanta.

[67] Clifford S., Lawrance G.A., Neuhold Y.-M., Maeder M. (2010). Conjoint Analysis of Kinetic and Equilibrium Data for Mechanistic Elucidation in Polynuclear Complexation Reactions, Exemplified by Metal(II) Helicate Complex Formation. Aust. J. Chem..

[68] Maeder M., King P., Varmuza K. (2012). Analysis of Chemical Processes, Determination of the Reaction Mechanism and Fitting of Equilibrium and Rate Constants. Chemometrics in Practical Applications.

[69] Piguet C. (2010). Five Thermodynamic Describers for Addressing Serendipity in the Self-Assembly of Polynuclear Complexes in Solution. Chem. Commun..

[70] Benson S.W. (1958). Statistical Factors in the Correlation of Rate Constants and Equilibrium Constants. J. Am. Chem. Soc..

[71] Baudet K., Kale V., Mirzakhani M., Babel L., Naseri S., Besnard C., Nozary H., Piguet C. (2020). Neutral Heteroleptic Lanthanide Complexes for Unravelling Host-Guest Assemblies in Organic Solvents: The Law of Mass Action Revisited. Inorg. Chem..

[72] Chowdhury T., Horsewill S.J., Wilson C., Farnaby J.H., Chowdhury T., Horsewill S.J., Wilson C., Farnaby J.H. (2022). Heteroleptic Lanthanide(III) Complexes: Synthetic Utility and Versatility of the Unsubstituted Bis-Scorpionate Ligand Framework. Aust. J. Chem..

[73] Abdus Subhan M., Suzuki T., Kaizaki S. (2001). Stereospecific Assembly of Chiral Λ-Cr(III)-Δ-Ln(III)-Oxalato-Bridged Dinuclear 3d–4f Complexes (Ln = Yb or Dy) and near Infrared Circular Dichroism in the 4f→4f Transitions. J. Chem. Soc. Dalton Trans..

[74] Abdus Subhan M., Suzuki T., Kaizaki S. (2002). Solution NIR CD and MCD in 4f–4f Transitions of a Series of Chiral 3d–4f Dinuclear Complexes: X-Ray Structures of (Λ-Δ)-[(Acac) 2 Cr III (μ-Ox)Ln III (HBpz 3) 2] (Ln = Sm, Ho and Er). J. Chem. Soc. Dalton Trans..

[75] Pinsky M., Avnir D. (1998). Continuous Symmetry Measures. 5. The Classical Polyhedra. Inorg. Chem..

[76] Casanova D., Cirera J., Llunell M., Alemany P., Avnir D., Alvarez S. (2004). Minimal Distortion Pathways in Polyhedral Rearrangements. J. Am. Chem. Soc..

[77] Jordan R.B. (2023). Lanthanide Contraction: What is Normal?. Inorg. Chem..

[78] Ferguson J., Tobe M.L. (1970). Complexes of Chromium(III) with a Cyclic Tetradentate Secondary Amine. Inorg. Chim. Acta.

[79] Bakac A., Espenson J.H. (1992). A High-Yield One-Step Synthesis of Dichloro(Tetraazacyclotetradecane)Chromium(1+) Chloride (Trans-[Cr([14]aneN_4_)Cl_2_]Cl) and Its Conversion to Trans-[Cr([14]aneN_4_)(H_2_O)_2_](CF_3_SO_3_)_3_. Inorg. Chem..

[80] Strickler S.J., Berg R.A. (1962). Relationship between Absorption Intensity and Fluorescence Lifetime of Molecules. J. Chem. Phys..

[81] Birks J.B., Dyson D.J. (1997). The Relations between the Fluorescence and Absorption Properties of Organic Molecules. Proc. R. Soc. Lond. Ser. Math. Phys. Sci..

[82] Bünzli J.-C.G., Chauvin A.-S., Kim H.K., Deiters E., Eliseeva S.V. (2010). Lanthanide Luminescence Efficiency in Eight- and Nine-Coordinate Complexes: Role of the Radiative Lifetime. Coord. Chem. Rev..

[83] Jimenez J.R., Poncet M., Doistau B., Besnard C., Piguet C. (2020). Luminescent Polypyridyl Heteroleptic Cr(III) Complexes with High Quantum Yields and Long Excited State Lifetimes. Dalton Trans..

[84] Otto S., Dorn M., Förster C., Bauer M., Seitz M., Heinze K. (2018). Understanding and Exploiting Long-Lived Near-Infrared Emission of a Molecular Ruby. Coord. Chem. Rev..

[85] Starzak M.E. (1989). Mathematical Methods in Chemistry and Physics.

[86] Bolvin H., Furstenberg A., Golesorkhi B., Nozary H., Taarit I., Piguet C. (2022). Metal-Based Linear Light Upconversion Implemented in Molecular Complexes: Challenges and Perspectives. Acc. Chem. Res..

[87] Golesorkhi B., Fürstenberg A., Nozary H., Piguet C. (2019). Deciphering and Quantifying Linear Light Upconversion in Molecular Erbium Complexes. Chem. Sci..

[88] Charbonnière L.J., Nonat A.M., Knighton R.C., Godec L. (2024). Upconverting Photons at the Molecular Scale with Lanthanide Complexes. Chem. Sci..

[89] Sun R., Sun L., Bünzli J.-C.G., Kauzlarich S.M. (2024). Rare-earth Upconversion Luminescence and its Application: From Molecular to Nano and Micro Scales. Handbook on the Physics and Chemistry of Rare Earths.

[90] Lu W., Yan W.C., Bian Z.Q., Liu Z.W. (2026). Upconversion Luminescence of Molecular Lanthanide Complexes. Chem. Eur. J..

[91] Sheldrick G.M. (2015). SHELXT-Intergrated space-group and crystal-structure determination. Acta Cryst. A.

[92] Sheldrick G.M. (2015). Crystal Structure Refinement with SHELXL. Acta Cryst. C.

[93] Sun C., Turlington C.R., Thomas W.W., Wade J.H., Stout W.M., Grisenti D.L., Forrest W.P., VanDerveer D.G., Wagenknecht P.S. (2011). Synthesis of Cis and Trans Bis-Alkynyl Complexes of Cr(III) and Rh(III) Supported by a Tetradentate Macrocyclic Amine: A Spectroscopic Investigation of the M(III)–Alkynyl Interaction. Inorg. Chem..

