# Supplementary material for: Heteroleptic [CrIIIN6] Chromophores as Partners for Lanthanide-Based Light Conversion in d-f Molecular Complexes
Source: Molecules. 2026 Jun 9;31(12):2016. doi: 10.3390/molecules31122016 (PMC13304974; doi:10.3390/molecules31122016)
Supplement: Supplementary file 1 [file molecules-31-02016-s001.zip › molecules-4340794-supplementary.pdf]

Heteroleptic [$Cr^{III}N_6$] Chromophores as Partners for Lanthanide-Based Light-Conversion in d-f Molecular Complexes

---

Julien Chong, Inès Taarit, Laure Guénée, Arnulf Rosspeintner and Claude Piguet*

Supporting Information

(57 pages)

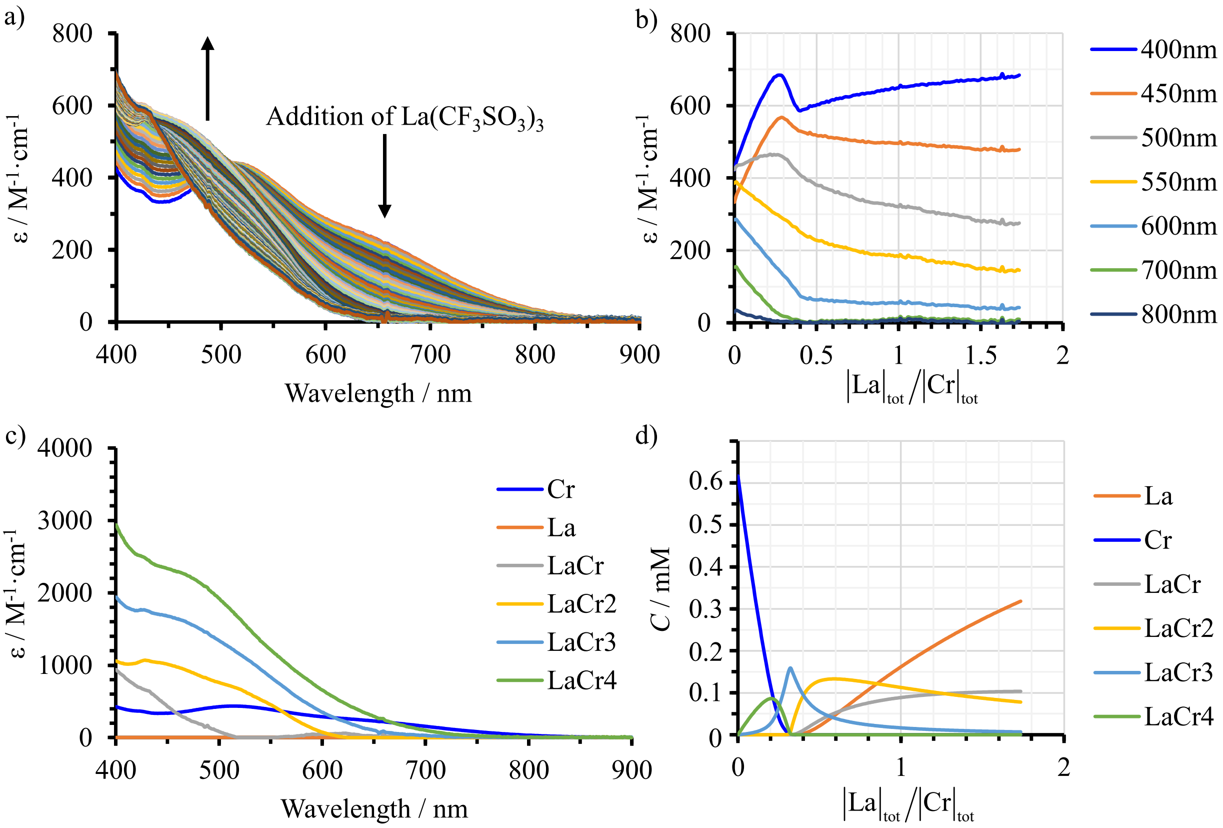

Figure S1. Evolutions of a) absorption spectra (normalized for the total concentration of Cr) and b) related absorbance at selected wavelengths after each addition of La(OTf)$_3$ to [(phen)$_2$Cr(biim)](OTf) ($6\cdot10^{-4}$ M in CH$_3$CN). c) Extracted absorption spectra for the absorbing species (Cr and LaCr$_n$) and e) evolution of concentrations of each species along the titration.

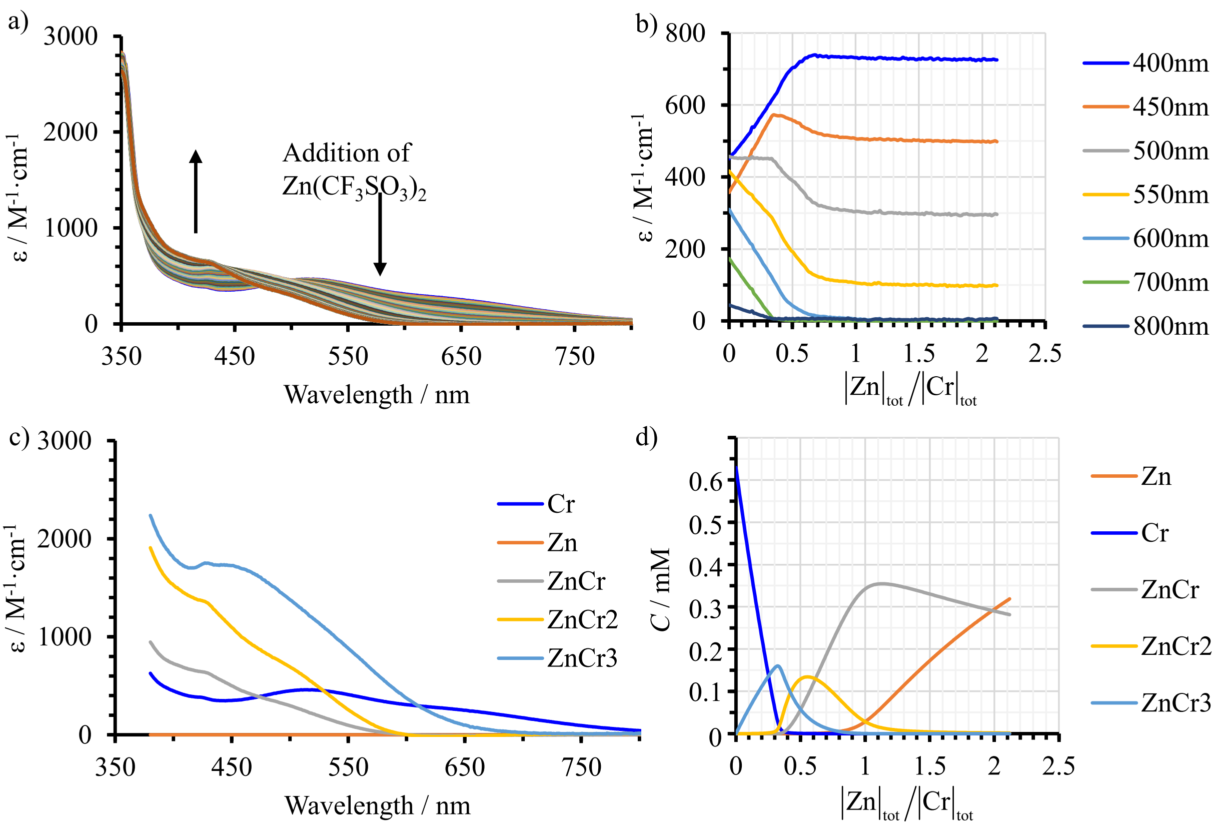

Figure S2. Evolutions of a) absorption spectra (normalized for the total concentration of Cr) and b) related absorbance at selected wavelengths after each addition of $Zn(OTf)_2$ to $[(phen)_2Cr(biim)](OTf)$ ($6\cdot10^{-4}$ M in CH_3CN). c) Extracted absorption spectra for the absorbing species (Cr and $ZnCr_n$) and e) evolution of concentrations of each species along the titration.

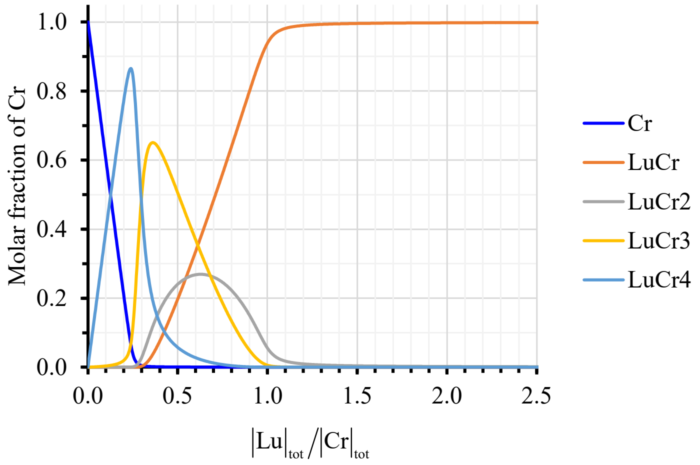

Figure S3. Molar fraction of the ligand Cr ([(phen)$_2$Cr(biim)]$^+$) under each forms (Cr, LuCr, LuCr$_2$, LuCr$_3$, LuCr$_4$) as a function of the number of equivalent of Lu(OTf)$_3$ added for a solution with a total Cr concentration fixed at 10^{-3} M. Computed by using the formation constants gathered in Table 1 with the software HySS2009 (Alderighi, L.; Gans, P.; Ienco, A.; Peters, D.; Sabatini, A.; Vacca, A. Hyperquad Simulation and Speciation (HySS): A Utility Program for the Investigation of Equilibria Involving Soluble and Partially Soluble Species. *Coord. Chem. Rev.* **1999**, *184*, 311–318).

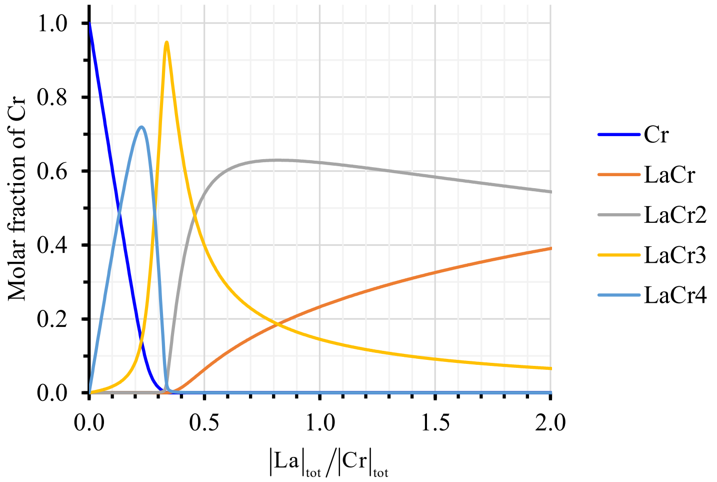

Figure S4. Molar fraction of the ligand Cr ([(phen)$_2$Cr(biim)]$^+$) under each forms (Cr, LaCr, LaCr$_2$, LaCr$_3$, LaCr$_4$) as a function of the number of equivalent of La(OTf)$_3$ added for a solution with a total Cr concentration fixed at 10^{-3} M. Computed by using the formation constants gathered in Table 1 with the software HySS2009 (Alderighi, L.; Gans, P.; Ienco, A.; Peters, D.; Sabatini, A.; Vacca, A. Hyperquad Simulation and Speciation (HySS): A Utility Program for the Investigation of Equilibria Involving Soluble and Partially Soluble Species. *Coord. Chem. Rev.* **1999**, *184*, 311–318).

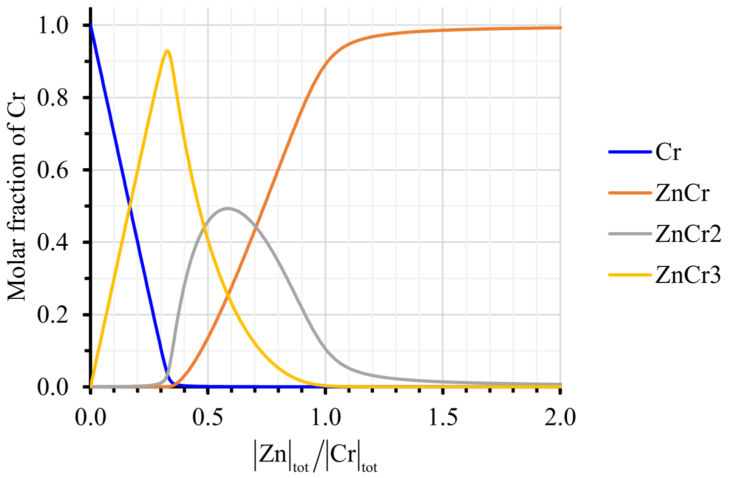

Figure S5. Molar fraction of the ligand Cr ($[(phen)_2Cr(biim)]^+$) under each forms (Cr, ZnCr, $ZnCr_2$, $ZnCr_3$) as a function of the number of equivalent of $Zn(OTf)_2$ added for a solution with a total Cr concentration fixed at 10^{-3} M. Computed by using the formation constants gathered in Table 1 with the software HySS2009 (Alderighi, L.; Gans, P.; Ienco, A.; Peters, D.; Sabatini, A.; Vacca, A. Hyperquad Simulation and Speciation (HySS): A Utility Program for the Investigation of Equilibria Involving Soluble and Partially Soluble Species. *Coord. Chem. Rev.* **1999**, *184*, 311–318).

Supplementary File S1. Symmetry number method

The statistical contributions of each association constants K_{stat} were determined with the symmetry number method developed by Benson [70] and Ercolnai [A1-1]. For the reaction of Zn^{2+} with $[(phen)_2Cr(biim)](OTf)$ (referred to as Cr), it is assumed that the Zn^{2+} ion always adopts an octahedral geometry with Cr acting as bidentate ligands and CH_3CN as monodentate solvent molecules completing the coordination sphere. With this assumption, the solution can contain the following species: Cr, CH_3CN, $[Zn(CH_3CN)_6]^{2+}$, $[ZnCr(CH_3CN)_4]^{3+}$, $[ZnCr_2(CH_3CN)_2]^{4+}$ and $[ZnCr_3]^{5+}$. Note that the complex $[ZnCr_2(CH_3CN)_2]^{4+}$ has two isomers *cis*- and *trans*- which should be considered independently for the determination of their symmetry numbers. $[(phen)_2Cr(biim)]^+$ can take two enantiomeric forms Δ and Λ, this chirality source also induces some stereoisomers and enantiomers on MCr_n complexes. But for the sake of simplicity, we will assume Cr to behave like an achiral bidentate ligand, with a C_{2v} symmetry. For each species considered, there are three associated values: σ_{ext}, σ_{int} and σ_{mix} (Table S1). The external symmetry number is given by the number of different but indistinguishable atomic arrangements that can be obtained by rotating a given molecule. It can be obtained by multiplying the order of independent symmetry operations of the first kind related to the molecule point group. The internal symmetry number σ_{int} corresponds to the number of different but indistinguishable atomic arrangements obtained by internal rotations around single bounds. Finally, σ_{mix} accounts for the number of chemically distinguishable isomeric species. In our case, σ_{mix} = ½ if the molecule is chiral, σ_{mix} = 1 otherwise. σ_{tot} is the product of the three (Equation A1-1).

$$\sigma_{tot} = \sigma_{ext} \cdot \sigma_{int} \cdot \sigma_{mix} \quad \text{(A1-1)}$$

Table S1. Symmetry point group, and σ values associated for each molecule considered in the reaction of Zn^{2+} with Cr.

Molecule	$[Zn(CH_3CN)_6]^{2+}$	Cr	$[ZnCr(CH_3CN)_4]^{3+}$	$[ZnCr_2(CH_3CN)_2]^{4+}$		$[ZnCr_3]^{5+}$	CH_3CN
Isomers	1	1	1	2		1	1
Group	O_h	C_{2v}	C_{2v}	C_2	D_{2h}	D_3	C_{3v}
σ_{ext}	24	2	2	2	4	6	3
σ_{int}	3^6	1	3^4	3^2	3^2	1	1
σ_{mix}	1	1	1	1/2	1	1/2	1
σ_{tot}	17496	2	162	9	36	3	3

For a given reaction:

$$aA + bB \rightarrow cC + dD \quad \text{(A1-2)}$$

The rotational statistical factors (ω) is defined as the ratio of σ_{tot} of the reactants to the products one each raised to their respective stoichiometric coefficients

$$\omega = \frac{(\sigma_{tot}^{A})^{a} \cdot (\sigma_{tot}^{B})^{b}}{(\sigma_{tot}^{C})^{c} \cdot (\sigma_{tot}^{D})^{d}} \quad \text{(A1-3)}$$

The statistical constant K_{stat} for each reaction is equal to ω for the reactions where only one isomer is formed. Because we assume all the isomers are isoenergetic for simplicity (i.e. $\Delta E_{Cr,Cr}$ and $\Delta G_{M,Cr}$ are independent of the geometrical arrangement of the ligands (equation 7 in the main text), if there is more than one possible isomer formed, K_{stat} is the sum of all ω_i for each isomer:

$$K_{stat} = \sum \omega_i \quad \text{(A1-4)}$$

$$[Zn(CH_3CN)_6]^{2+} + Cr \xrightarrow{K_{stat}=24} [ZnCr(CH_3CN)_4]^{3+} + 2\ CH_3CN \quad \text{(A1-5)}$$

$$[Zn(CH_3CN)_6]^{2+} + 2Cr \xrightarrow{K_{stat}=96+24=120} [ZnCr_2(CH_3CN)_2]^{4+} + 4\ CH_3CN \quad \text{(A1-6)}$$

$$[Zn(CH_3CN)_6]^{2+} + 3Cr \xrightarrow{K_{stat}=64} [ZnCr_3]^{5+} + 6\ CH_3CN \quad \text{(A1-7)}$$

For the reaction of Ln^{3+} with Cr, we assume a square antiprism geometry arrangement of the coordination sphere as it has been found in the crystal structures of $[(phen)_2Cr(biim)Ln(Tp)_2]^{2+}$ (Ln = Y, Eu and Er) (Figure 5). With this assumption, there are 22 possible species of $[LnCr_n(CH_3CN)_{(8-2n)}]^{(3+n)+}$ with n = 0-4, where bidentate ligands occupy one of the edges and the solvents molecules occupy the remaining vertices of the square antiprism (Table S2).

Applying the equations, we find the four K_{stat} values for the reactions of Ln^{3+} + Cr:

$$[Ln(CH_3CN)_8]^{3+} + Cr \xrightarrow{K_{stat}=32} [LnCr(CH_3CN)_6]^{3+} + 2\ CH_3CN \quad \text{(A1-8)}$$

$$[Ln(CH_3CN)_8]^{3+} + 2Cr \xrightarrow{K_{stat}=288} [LnCr_2(CH_3CN)_4]^{5+} + 4\ CH_3CN \quad \text{(A1-9)}$$

$$[Ln(CH_3CN)_8]^{3+} + 3Cr \xrightarrow{K_{stat}=702} [LnCr_3(CH_3CN)_4]^{6+} + 6\ CH_3CN \quad \text{(A1-10)}$$

$$[Ln(CH_3CN)_8]^{3+} + 4Cr \xrightarrow{K_{stat}=224} [LnCr_4]^{6+} + 8\ CH_3CN \quad \text{(A1-11)}$$

Reference

[A1-1] Ercolani, G.; Piguet, C.; Borkovec, M.; Hamacek, J. Symmetry Numbers and Statistical Factors in Self-Assembly and Multivalency. *J. Phys. Chem. B* **2007**, *111*, 12195-12203. DOI: 10.1021/jp0740705.

Table S2. Symmetry point group, and σ values associated for each molecule considered in the reaction of Ln^{3+} (Ln = Lu or La) with Cr.

Molecule	$[Ln(CH_3CN)_8]^{3+}$	Cr	$[LnCr(CH_3CN)_6]^{4+}$		$[LnCr_2(CH_3CN)_4]^{5+}$								$[LnCr_3(CH_3CN)_2]^{6+}$								$[LnCr_4]^{7+}$			CH_3CN
Number of isomers	1	1	2		8								8								3			1
Point group	D_{4d}	C_{2v}	C_2	C_s	D_2	C_2	C_s	C_1	C_1	C_2	C_2	C_{2v}	C_1	C_1	C_2	C_2	C_1	C_s	C_2	C_2	D_4	D_2	C_2	C_{3v}
σ_{ext}	8	2	2	1	4	2	1	1	1	2	2	2	1	1	2	2	1	1	2	2	8	4	2	3
σ_{int}	3^8	1	3^6	3^6	3^4	3^4	3^4	3^4	3^4	3^4	3^4	3^4	3^2	3^2	3^2	3^2	3^2	3^2	3^2	3^2	1	1	1	1
σ_{mix}	1	1	1/2	1	1/2	1/2	1	1/2	1/2	1/2	1/2	1	1/2	1/2	1/2	1/2	1/2	1	1/2	1/2	1/2	1/2	1/2	1
σ_{tot}	52488	2	729	729	162	81	81	81/2	81/2	81	81	162	9/2	9/2	9	9	9/2	9	9	9/2	4	2	1	3

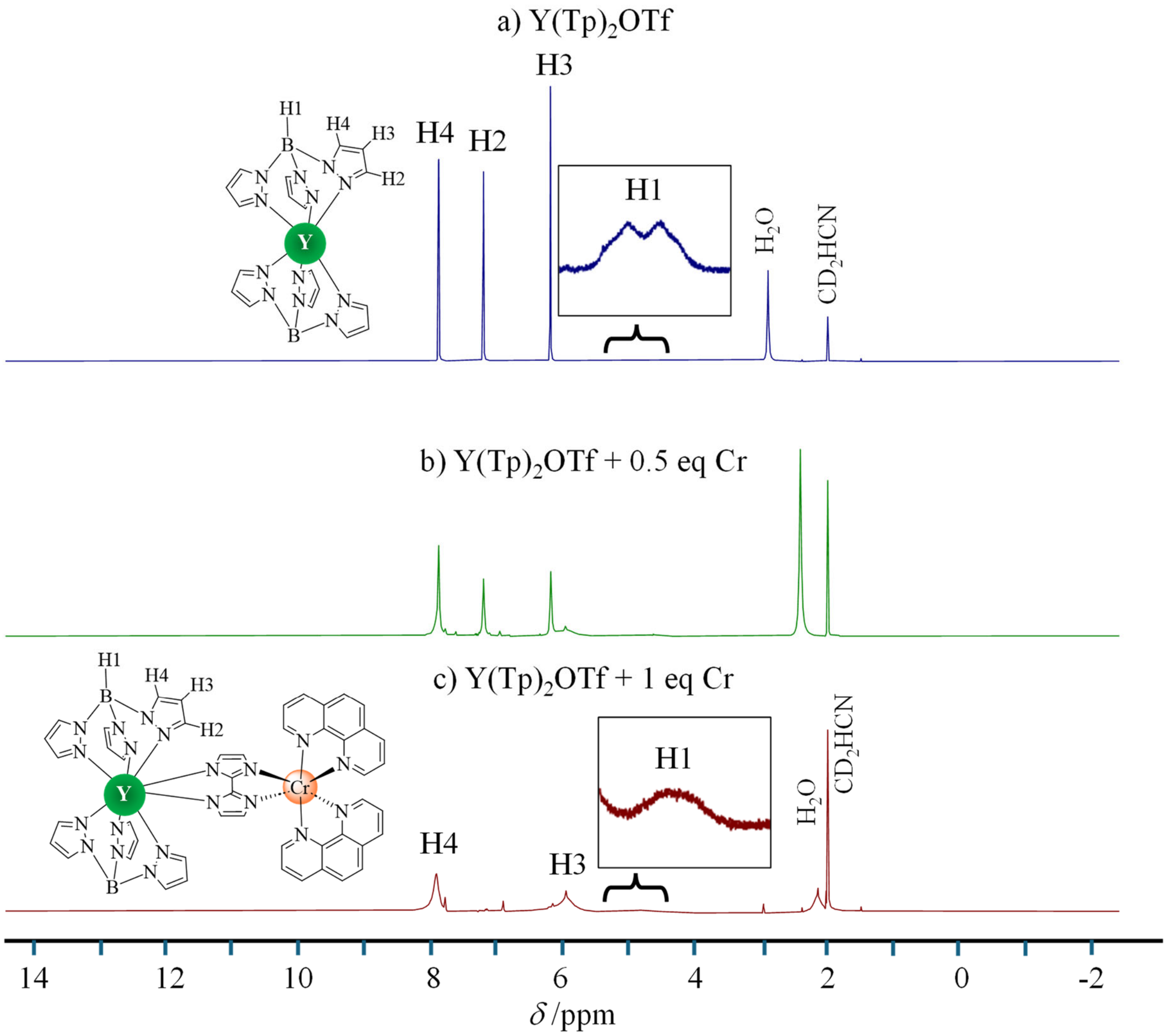

Figure S6. ^{1}H-NMR spectra recorded for a) Y(Tp)$_2$(OTf), b) Y(Tp)$_2$(OTf) + 0.5 eq [(phen)$_2$Cr(biim)](OTf) and c) Y(Tp)$_2$(OTf) + 1 eq [(phen)$_2$Cr(biim)](OTf) in CD_3CN at 293 K.

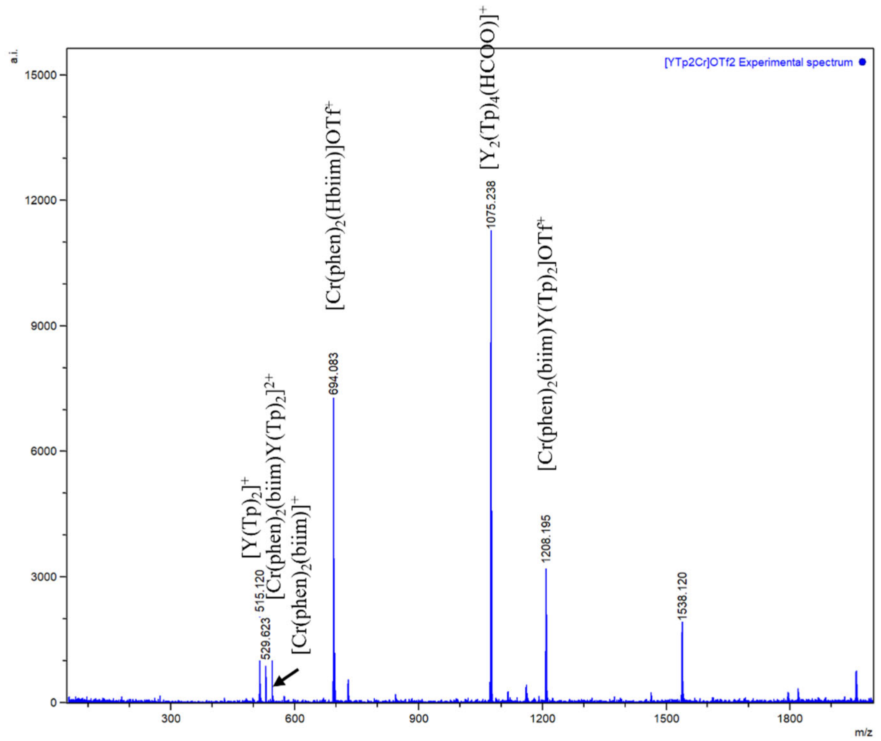

Figure S7. ESI-HRMS full spectrum of a solution of [(phen)2Cr(biim)]OTf with 1 equivalent of Y(Tp)2OTf (CH_3CN; $c = 5\cdot10^{-3}$M).

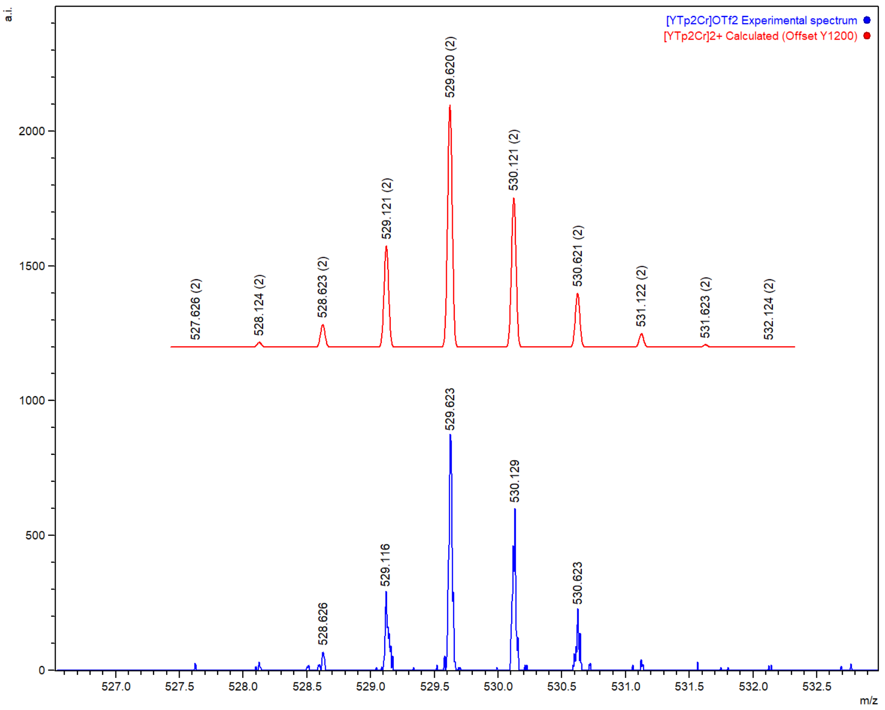

Figure S8. Zoom on the experimental HRMS spectrum of [(phen)2Cr(biim)]OTf with 1 equivalent of Y(Tp)2OTf (blue trace). Calculated (red trace) spectrum of [(phen)2Cr(biim)Y(Tp)2] $^{2+}$.

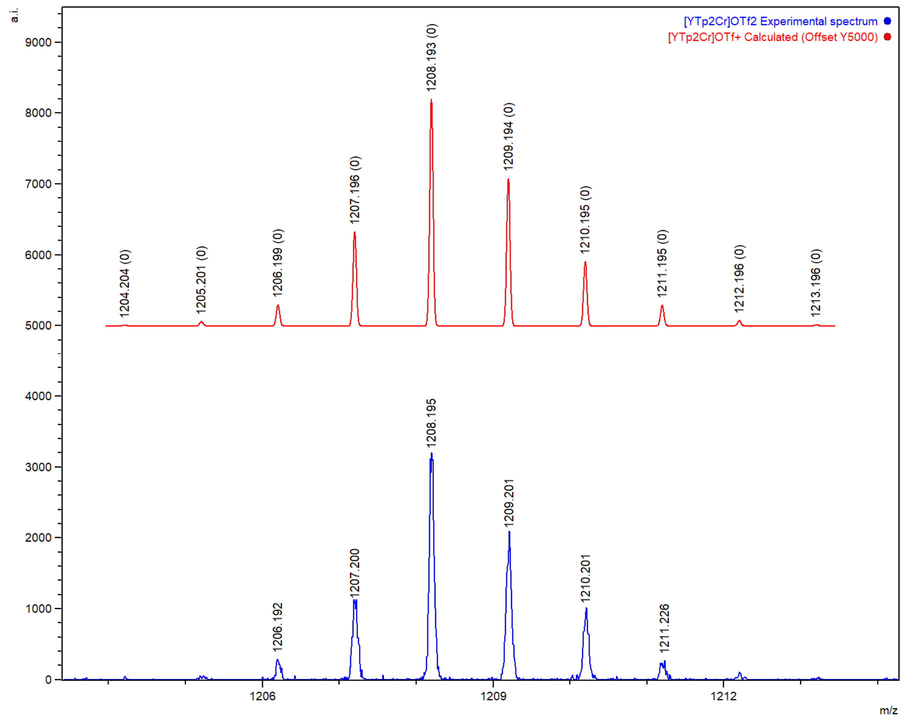

Figure S9. Zoom on the experimental HRMS spectrum of [(phen)2Cr(biim)]OTf with 1 equivalent of Y(Tp)2OTf (blue trace). Calculated (red trace) spectrum of [(phen)2Cr(biim)Y(Tp)2]OTf^{+}.

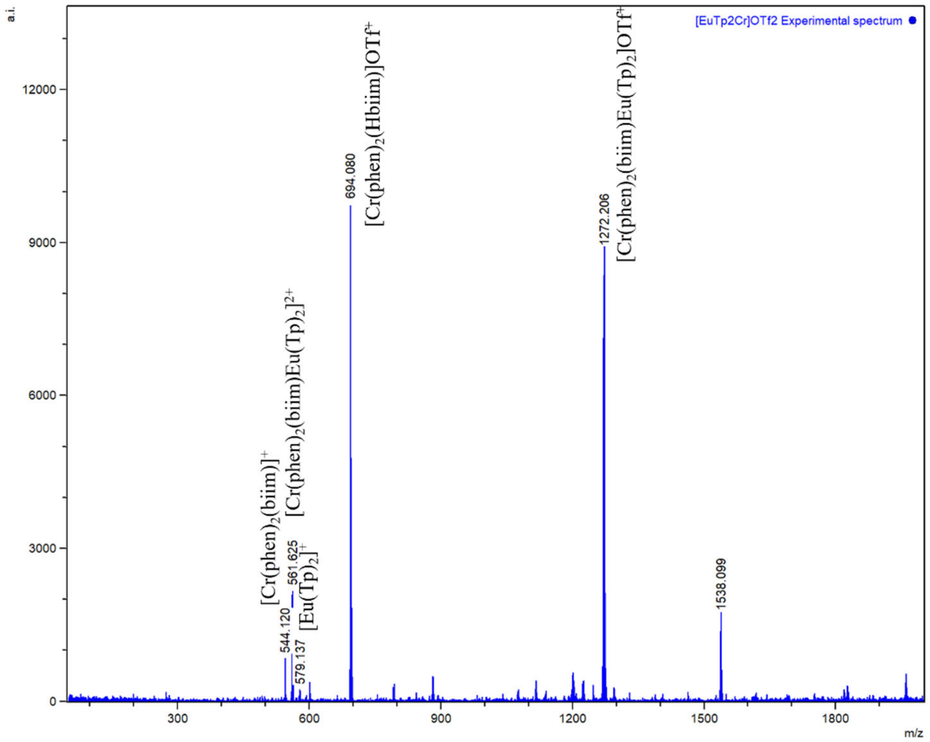

Figure S10. ESI-HRMS full spectrum of a solution of [(phen)2Cr(biim)]OTf with 1 equivalent of Eu(Tp)2OTf (CH_3CN; $c = 5 \cdot 10^{-3}$M).

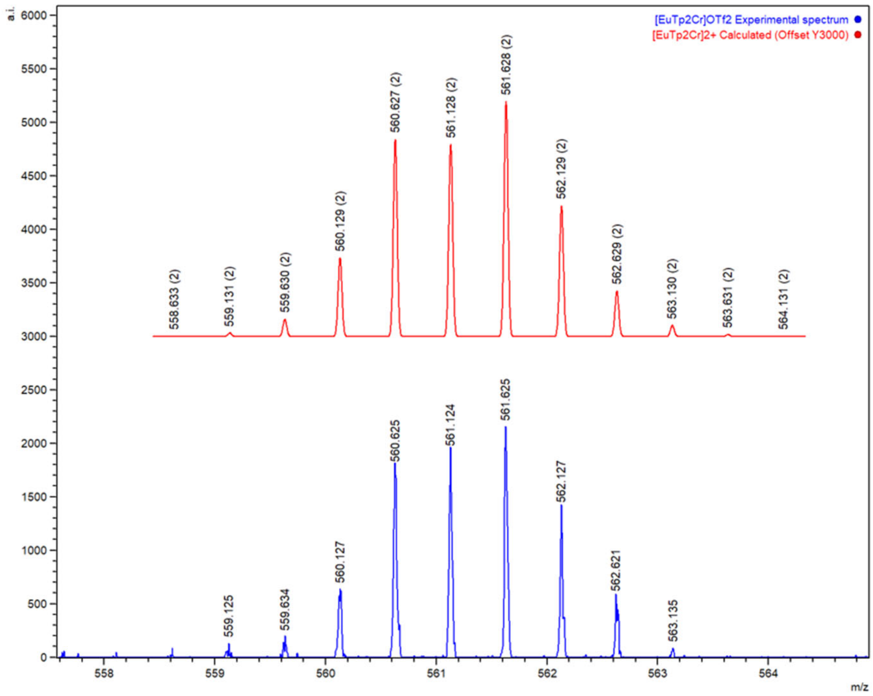

Figure S11. Zoom on the experimental HRMS spectrum of [(phen)$_2$Cr(biim)]OTf with 1 equivalent of Eu(Tp)$_2$OTf (blue trace). Calculated (red trace) spectrum of [(phen)$_2$Cr(biim)Eu(Tp)$_2$]$^{2+}$.

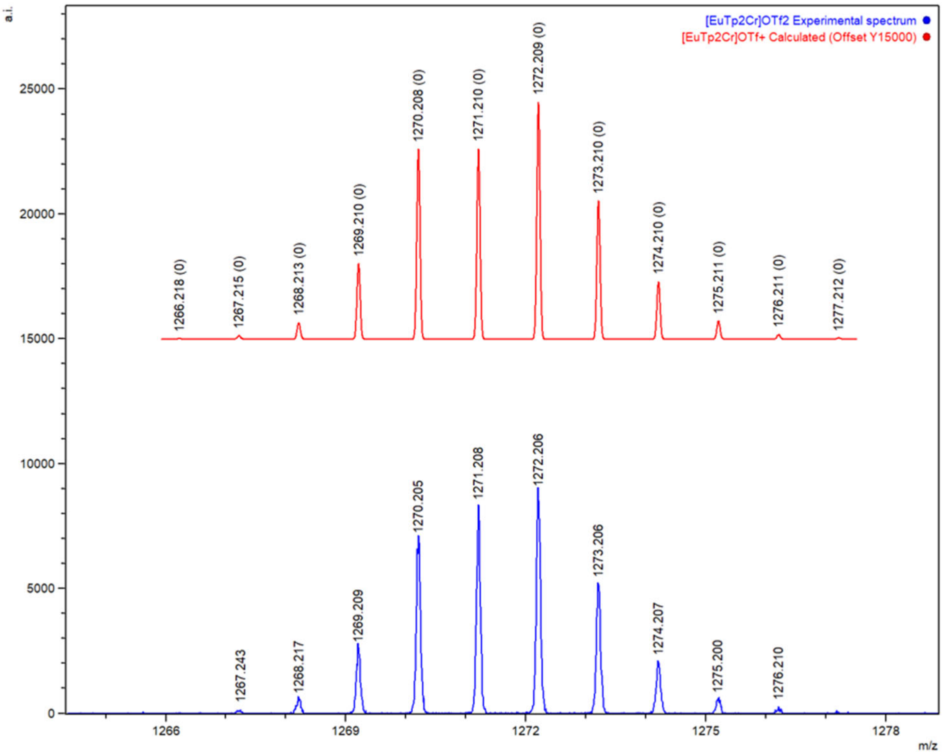

Figure S12. Zoom on the experimental HRMS spectrum of [(phen)$_2$Cr(biim)]OTf with 1 equivalent of Eu(Tp)$_2$OTf (blue trace). Calculated (red trace) spectrum of [(phen)$_2$Cr(biim)Eu(Tp)$_2$]OTf^{+}.

Supplementary File S2. Spectrophotometric titrations of [(phen)$_2$Cr(biim)]OTf with Ln(Tp)$_2$OTf

The ^{1}H-NMR and HRMS spectra indicate that the Cr complex coordinates to the $[Ln(Tp)_2]^{+}$ moiety in solution. In addition, when a solution of [Cr(phen)$_2$(biim)]OTf is mixed with a solution of Ln(Tp)$_2$OTf, a color change is observed. We therefore exploited the spectrophotometry method to extract the binding constant

$$\beta_{1,1}^{\mathrm{TpLn,Cr}} = \frac{[\mathrm{TpLnCr}]}{[\mathrm{Cr}]\cdot[\mathrm{TpLn}]} \quad \text{(A2-1)}$$

where TpLnCr denotes $[(phen)_2Cr(biim)Ln(Tp)_2]^{2+}$, TpLn denotes $[Ln(Tp)_2]^{+}$ and Cr denotes $[(phen)_2Cr(biim)]^{+}$.

Solutions of the complex [(phen)$_2$Cr(biim)](OTf) at ~ $6\cdot10^{-4}$ M in CH_3CN were titrated with a solution of the Y(Tp)$_2$OTf complex, while the absorption spectrum of the solution in the visible was monitored with an absorption probe using an optical path of 1 cm.

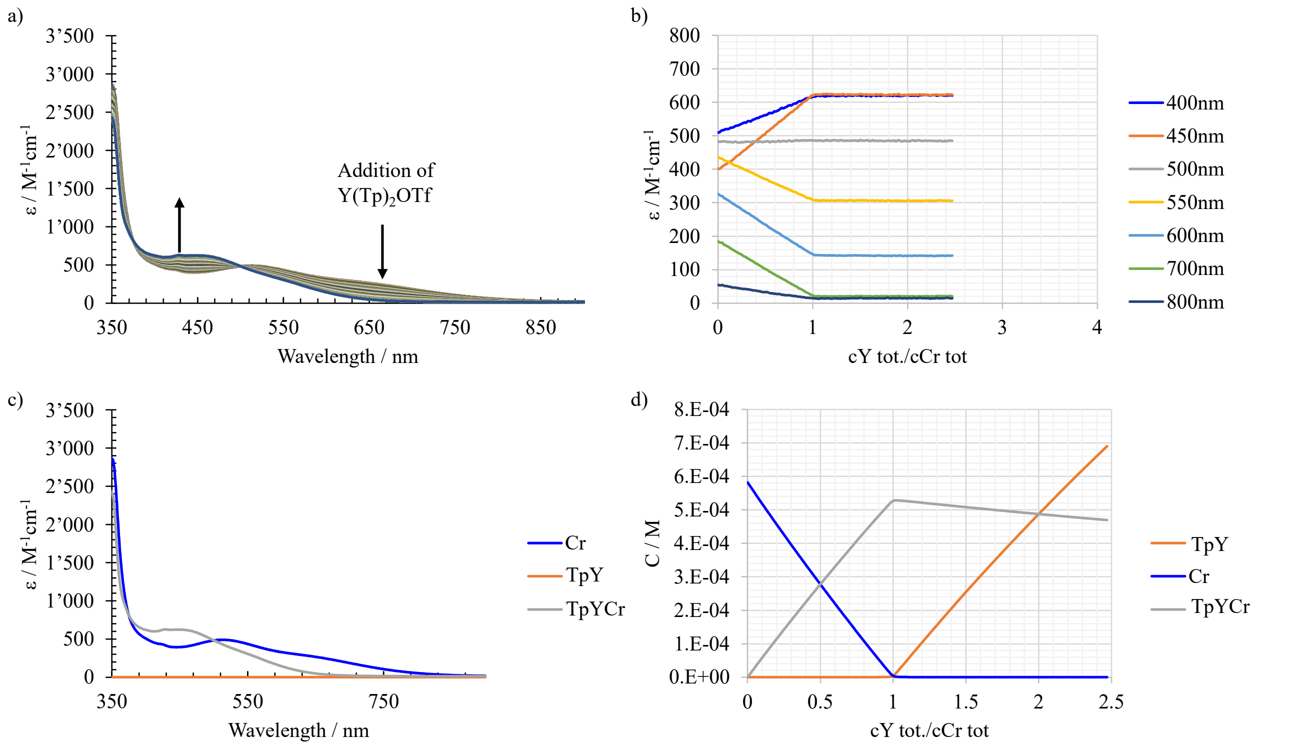

Figure S13. a) Evolution of the absorption spectrum of [(phen)$_2$Cr(biim)]OTf for each addition of the solution of Y(Tp)$_2$OTf, ε (y axis) is the absorbance divided by the total concentration of Cr in solution (corrected for dilution). b) Evolution of the absorbance of the solution at selected wavelengths as function of the number of equivalents of Y(Tp)$_2$OTf added ($c_{Y\ tot.}/c_{Cr\ tot.}$), ε (y axis) is the absorbance divided by the total concentration of Cr in solution (corrected for dilution). c) Extracted absorption spectra of the absorbing species (Cr and TpYCr). d) Evolution of concentrations of each species in solution as function of the ratio $c_{Y\ tot.}/c_{Cr\ tot.}$ during the titration.

Figure 13b indicates a linear change of the absorption spectrum of the solution upon addition of $Y(Tp)_2OTf$, until one equivalent is added. After this point, the absorption spectrum does not change anymore. This is a clear indication of the 1:1 stoichiometry ratio for the reaction. From these data, the absorption spectrum of YCr, the concentration profile of each species in solution and the association constant $\beta_{1,1}^{\mathrm{TpY,Cr}}$ = 7.6(3) could be extracted using non-linear least square fitting techniques (Figure S13). Figure S13d indicates that the formation of the complex is close to quantitative in these conditions

The titration of the chromium solution with $Eu(Tp)_2OTf$ gives similar results (Figure S14).

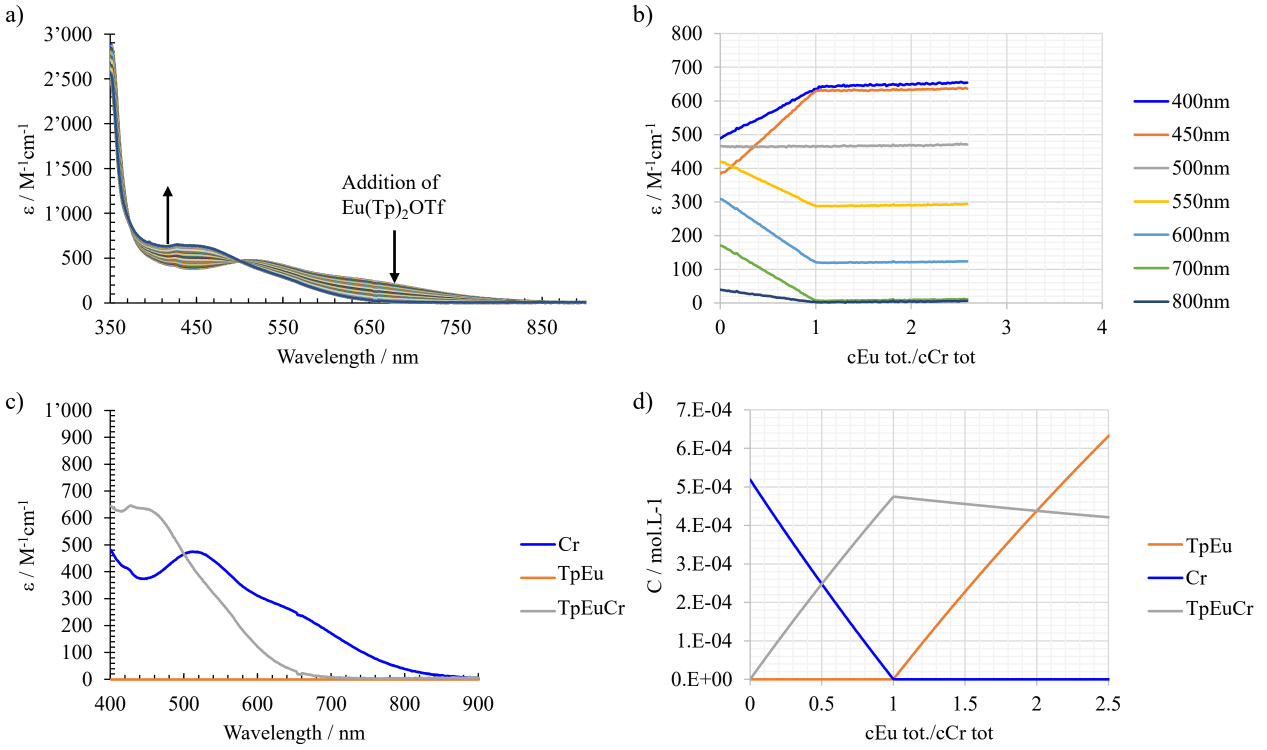

Figure S14. a) Evolution of the absorption spectrum of $[Cr(phen)_2(biim)]OTf$ for each addition of the solution of $Eu(Tp)_2OTf$, ε (y axis) is the absorbance divided by the total concentration of Cr in solution (corrected for dilution). b) Evolution of the absorbance of the solution at selected wavelengths as function of the number of equivalents of $Eu(Tp)_2OTf$ added ($c_{\mathrm{Eu\ tot.}}/c_{\mathrm{Cr\ tot.}}$), ε (y axis) is the absorbance divided by the total concentration of Cr in solution (corrected for dilution). c) Extracted absorption spectra of the absorbing species (Cr and TpEuCr). d) Evolution of concentrations of each species in solution as function of the ratio $c_{\mathrm{Eu\ tot.}}/c_{\mathrm{Cr\ tot.}}$ during the titration.

The concentration profile of each species in solution (Figure S14d) and the association constant $\beta_{1,1}^{\mathrm{TpEu,Cr}}$ = 8.7(7) could be extracted using non-linear least square fitting techniques.

Supplementary File S3. Crystal structures

A3.1. Crystal structures of the mononuclear heteroleptic complexes [(cyclam)Cr (H$_n$biim)]$^{(n+1)+}$

Single crystals suitable for XRD diffraction of [(cyclam)Cr(H$_2$biim)]OTf$_3$ (Figures S15 and S17) and [(cyclam)Cr(Hbiim)]OTf$_2$ (Figures S16 and S18) were obtained by slow diffusion of Et$_2$O into methanolic solutions of the corresponding complexes. The crystal structure of [(cyclam)Cr(biim)]OTf could not by resolved because the complex is too insoluble in solvents to obtain suitable single crystals by recrystallization.

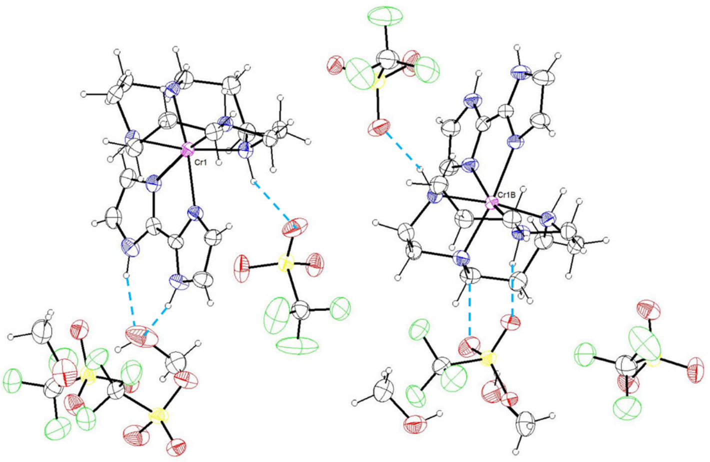

Figure S15. Ortep view of the asymmetric unit of [(cyclam)Cr(H$_2$biim)]OTf$_3$ (thermal ellipsoids are drawn at 50% probability).

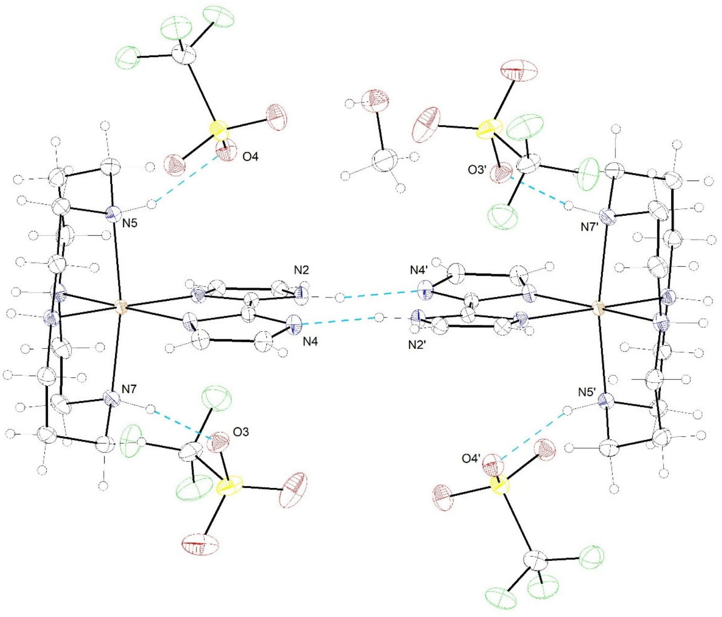

Figure S16. Ortep view of the asymmetric unit of [(cyclam)Cr(Hbiim)]OTf$_2$ highlighting the hydrogen bonds in the crystal structure (thermal ellipsoids are drawn at 50% probability).

In both crystal structures, we observe hydrogen bonds involving the N-H donors of the biimidazole ligands. For [(cyclam)Cr(H$_2$biim)]OTf$_3$, the N-H bonds do hydrogen bonds with methanol

molecules nearby (Figure S15); while for [(cyclam)Cr(Hbiim)]OTf_2, there are pairs of hydrogen bonds between two $Hbiim^-$ ligands of adjacent complexes (Figure S16). Similar pairs of hydrogen bonds were also observed in the crystal structures of $[Cr(Hbiim)]_3$, $[Cr(Hbiim)_2(biim)]PPh_4$ and $[Cr(phen)_2(Hbiim)]OTf_2$ [64]. Additionally, there are hydrogen bonds between the N-H of the cyclam ligands and the oxygen atoms of the triflate anions (d_{O-H} = 1.9-2.0 Å).

Table S3. Crystal data and structure refinement for [(cyclam)Cr(H_2biim)]OTf_3.

CCDC	2550803	
Empirical formula	C21 H38 Cr F9 N8 O11 S3	
Formula weight	897.77	
Temperature	119.99(10) K	
Wavelength	1.54184 Å	
Crystal system	Triclinic	
Space group	P -1	
Unit cell dimensions	a = 12.93774(11) Å	α = 89.5218(8)°.
	b = 13.91171(13) Å	β = 88.7451(7)°.
	c = 20.0682(2) Å	γ = 81.7749(7)°.
Volume	3573.94(6) $Å^3$	
Z	4	
Density (calculated)	1.669 Mg/m^3	
Absorption coefficient	5.298 mm^{-1}	
F(000)	1844	
Crystal size	0.53 x 0.11 x 0.08 mm^3	
Theta range for data collection	3.210 to 76.068°.	
Index ranges	-10<=h<=15, -17<=k<=17, -24<=l<=25	
Reflections collected	61861	
Independent reflections	14407 [R(int) = 0.0344]	
Completeness to theta = 67.684°	99.8 %	
Absorption correction	Analytical	
Max. and min. transmission	0.704 and 0.280	
Refinement method	Full-matrix least-squares on F^2	
Data / restraints / parameters	14407 / 1 / 961	
Goodness-of-fit on F^2	1.041	
Final R indices [I>2sigma(I)]	R1 = 0.0513, wR2 = 0.1358	
R indices (all data)	R1 = 0.0559, wR2 = 0.1391	
Extinction coefficient	n/a	
Largest diff. peak and hole	1.938 and -0.982 e.$Å^{-3}$	

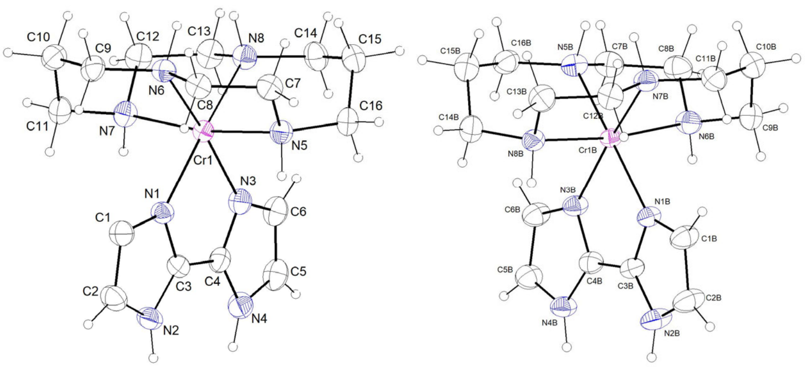

Figure S17. Ortep view of [(cyclam)Cr(H$_2$biim)]OTf$_3$ with mol A (left side) with N5(S) N6(S) N7(S) and N8(S) and mol B (right side) with with N5B(R) N6B(R) N7B(R) and N8B(R), numbering scheme.

Table S4. Hydrogen bonds for [(cyclam)Cr(H$_2$biim)]OTf$_3$ [Å and °].

D-H···A	*d*(D-H)	*d*(H···A)	*d*(D···A)	<(DHA)
N(2)-H(2)···O(1T)	0.88	2.57	3.094(3)	119.0
N(2)-H(2)···O(1S)	0.88	2.06	2.849(4)	148.9
N(4)-H(4)···O(1S)	0.88	2.01	2.813(4)	151.4
N(5)-H(5)···O(4T)	1.00	1.91	2.838(3)	152.3
N(6)-H(6)···O(2T)#1	1.00	2.35	3.102(3)	130.9
N(6)-H(6)···O(3T)#1	1.00	2.26	3.235(4)	163.0
N(7)-H(7)···O(7T)#2	1.00	2.15	3.040(4)	147.8
N(7)-H(7)···O(8T)#2	1.00	2.30	3.137(4)	140.2
N(8)-H(8)···O(3T)#1	1.00	1.90	2.886(4)	167.8
N(2B)-H(2B)···O(3S)#1	0.88	1.92	2.744(4)	154.4
N(4B)-H(4B)···O(13T)#3	0.88	2.59	3.103(3)	118.3
N(4B)-H(4B)···O(3S)#1	0.88	2.14	2.918(4)	147.1
N(5B)-H(5B)···O(18T)	1.00	2.04	2.932(3)	147.2
N(6B)-H(6B)···O(15T)	1.00	1.84	2.792(3)	158.5
N(7B)-H(7BA)···O(16T)	1.00	1.93	2.854(3)	152.9
N(8B)-H(8BA)···O(12T)#4	1.00	1.92	2.915(3)	170.1
O(2S)-H(2S)···O(11T)	0.84	2.18	2.896(4)	143.1
O(3S)-H(3S)···O(4S)	0.84	1.82	2.662(4)	174.4
O(4S)-H(4S)···S(6T)	0.84	3.07	3.798(3)	145.8
O(4S)-H(4S)···O(18T)	0.84	2.12	2.846(4)	144.6

Symmetry transformations used to generate equivalent atoms:

#1 x+1,y,z #2 x,y,z+1 #3 -x+2,-y+1,-z+1 #4 -x+1,-y+1,-z+1

Table S5. Crystal data and structure refinement for [(cyclam)Cr(Hbiim)]OTf_2.

CCDC	2550804	
Empirical formula	C19 H33 Cr F6 N8 O7 S2	
Formula weight	715.65	
Temperature	100.00(10) K	
Wavelength	1.54184 Å	
Crystal system	Monoclinic	
Space group	P 2_1/c	
Unit cell dimensions	a = 9.01496(5) Å	α= 90°.
	b = 17.15778(10) Å	β= 94.0797(5)°.
	c = 18.23680(11) Å	γ = 90°.
Volume	2813.66(3) $Å^3$	
Z	4	
Density (calculated)	1.689 Mg/m^3	
Absorption coefficient	5.611 mm^{-1}	
F(000)	1476	
Crystal size	0.35 x 0.11 x 0.05 mm^3	
Theta range for data collection	3.541 to 75.956°.	
Index ranges	-7≤h≤11, -21≤k≤21, -22≤l≤22	
Reflections collected	49924	
Independent reflections	5771 [R(int) = 0.0251]	
Completeness to theta = 67.684°	100.0 %	
Absorption correction	Analytical	
Max. and min. transmission	0.827 and 0.373	
Refinement method	Full-matrix least-squares on F^2	
Data / restraints / parameters	5771 / 0 / 390	
Goodness-of-fit on F^2	1.062	
Final R indices [I>2sigma(I)]	R1 = 0.0426, wR2 = 0.1117	
R indices (all data)	R1 = 0.0436, wR2 = 0.1123	
Extinction coefficient	n/a	
Largest diff. peak and hole	1.979 and -1.192 e.$Å^{-3}$	

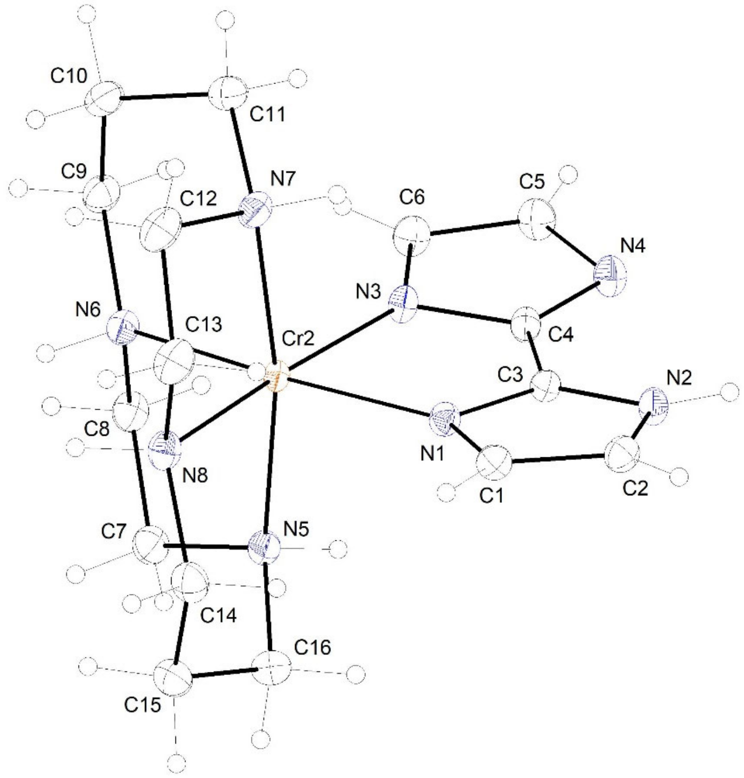

Figure S18. Ortep view of the complex [(cyclam)Cr(Hbiim)]OTf2 (thermal ellipsoids are drawn at 50% probability level) with a numbering scheme.

Table S6. Hydrogen bonds for [(cyclam)Cr(Hbiim)]OTf2 [Å and °].

D-H···A	*d*(D-H)	*d*(H···A)	*d*(D···A)	<(DHA)
N(2)-H(2)···N(4)#1	0.88	1.87	2.745(3)	169.3
N(5)-H(5)···O(4)	1.00	2.02	2.936(3)	150.3
N(5)-H(5)···O(6)	1.00	2.66	3.433(3)	134.4
N(6)-H(6)···O(1)#2	1.00	2.65	3.515(3)	145.0
N(6)-H(6)···O(2)#2	1.00	2.16	3.097(3)	156.0
N(7)-H(7)···O(3)	1.00	1.95	2.940(3)	169.6
N(8)-H(8)···O(2)#2	1.00	2.06	3.030(3)	162.0
O(7)-H(7C)···O(5)	0.84	2.02	2.845(3)	168.7

Symmetry transformations used to generate equivalent atoms:

#1 -x+1,-y+1,-z+1 #2 -x+2,y+1/2,-z+3/2

A3.2. Crystal structures of the heterodinuclear complexes

Several crystallization attempts of mixtures containing one equivalent of [(phen)$_2$Cr(biim)]OTf with one equivalent of Ln(Tp)$_2$OTf were performed in different conditions to obtain crystals suitable for X-ray diffraction (XRD). At first, the crystallization attempts lead to the unwanted protonated complex [Cr(phen)$_2$(Hbiim)](OTf)$_2$ (Figure S19a) [64]. This is believed to be the product of the hydrolysis of the complex due to traces amount of H_2O in the solvent where the chromium moiety takes a proton and crystallizes while the lanthanide takes the hydroxide and stays in solution. To remove traces of water, the crystallization was repeated while using molecular sieves in the solution (Figure S19b). Crystals of [(phen)$_2$Cr(biim)Ln(Tp)$_2$](OTf)$_2$ (Ln = Y in Figure S20; Ln = Eu in Figure S21) suitable for XRD could be then obtained. Both complexes [(phen)$_2$Cr(biim)Ln(Tp)$_2$](OTf)$_2$ (Y and Eu) crystallize in the triclinic P -1 space group with two co-crystallized CH_3CN molecules.

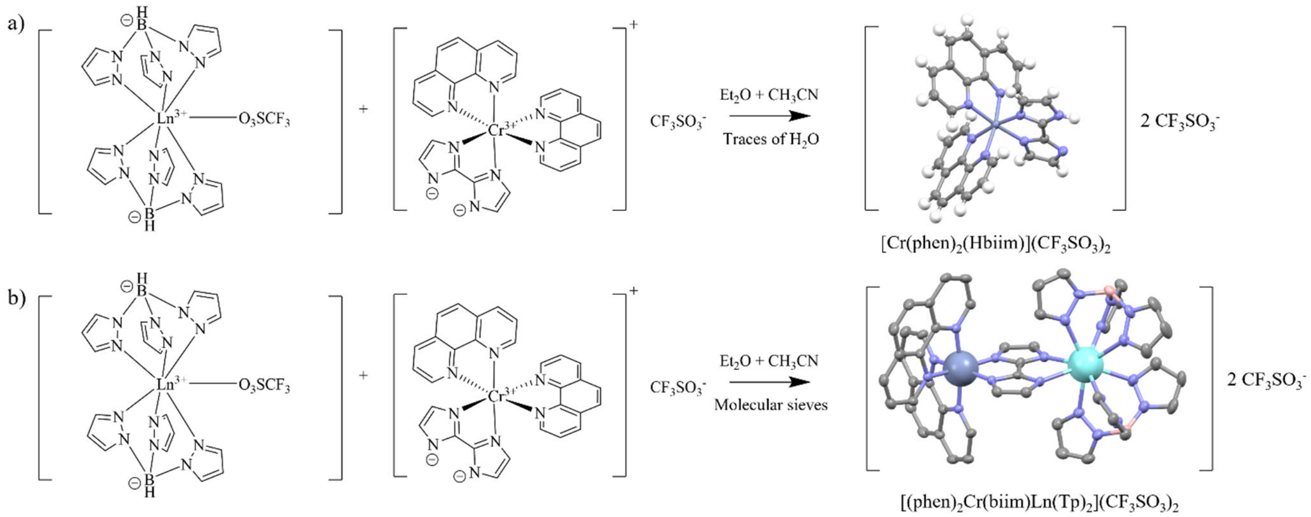

Figure S19. a) Crystallization attempts by vapor diffusion of Et_2O in non-anhydrous solution of 1.0 equivalent of [(phen)$_2$Cr(biim)]OTf with 1.0 equivalent of Ln(Tp)$_2$OTf in CH_3CN leading to the obtention of crystals of [(phen)$_2$Cr(Hbiim)](OTf)$_2$. b) Crystallization attempts by vapor diffusion of Et_2O in non-anhydrous solution of 1.0 equivalent of [(phen)$_2$Cr(biim)]OTf with 1.0 equivalent of Ln(Tp)$_2$OTf in CH_3CN dried with molecular sieves leading to the obtention of crystals of the desired complex [(phen)$_2$Cr(biim)Ln(Tp)$_2$](OTf)$_2$ (Figure S20 for Ln = Y, Figure S21 for Ln = Eu).

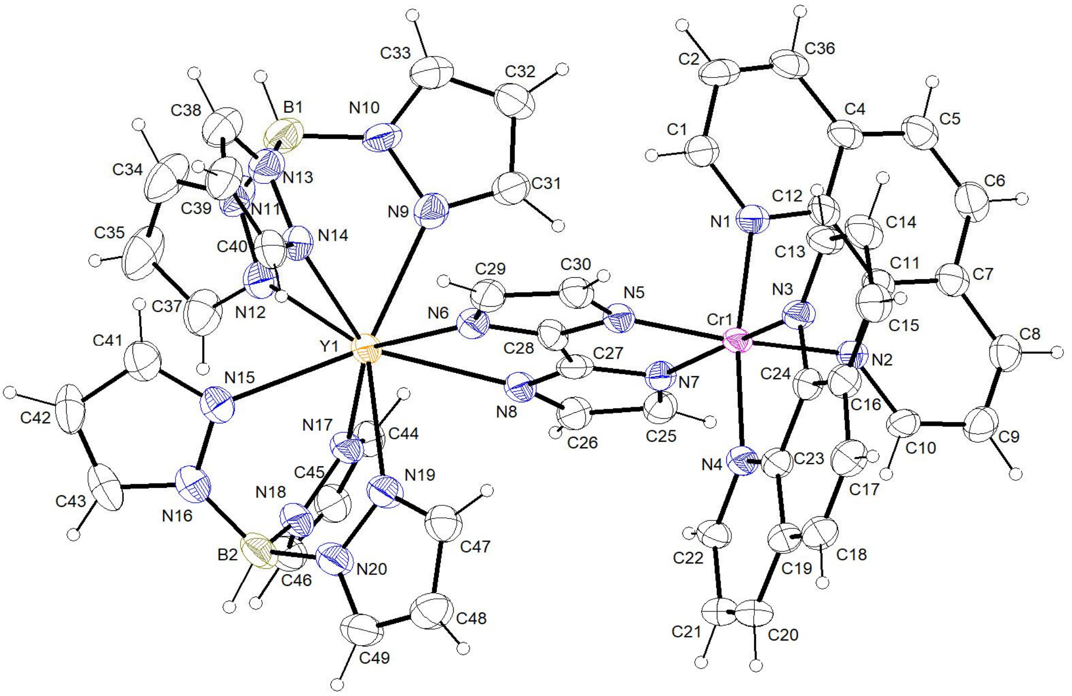

Figure S20. Ortep view of the complex [(phen)$_2$Cr(biim)Y(Tp)$_2$](OTf)$_2$·(CH$_3$CN)$_2$ (thermal ellipsoids are drawn at 50% probability level) with numbering scheme. Triflates counter ions and acetonitrile solvate molecules are omitted for clarity purposes.

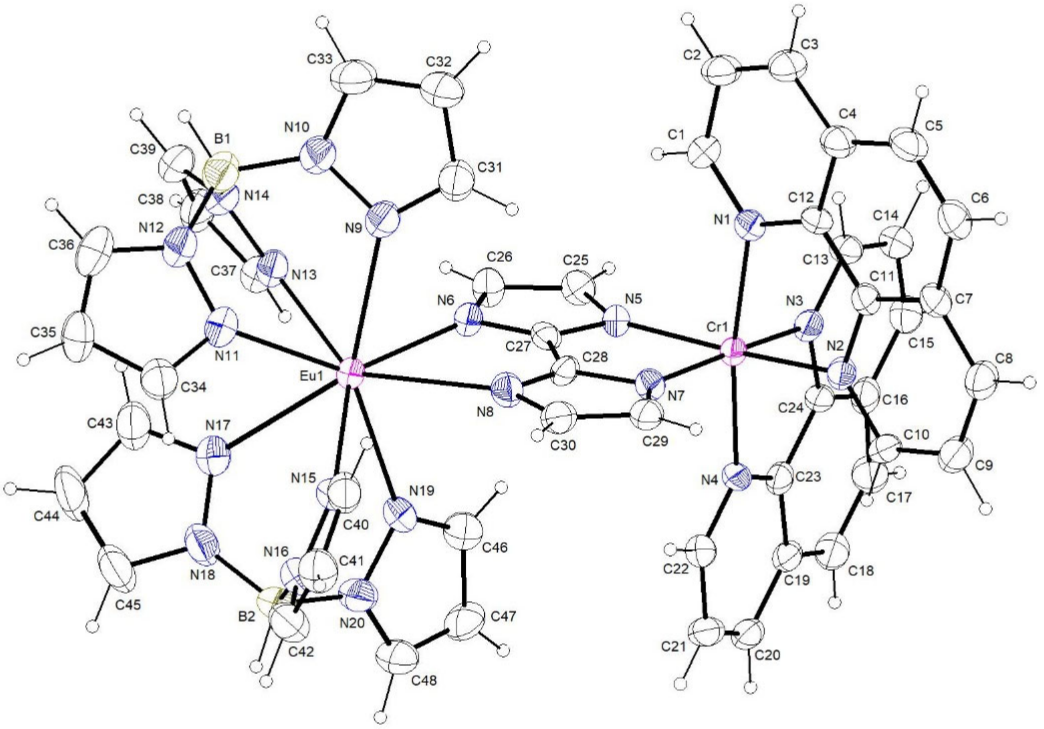

Figure S21. Ortep view of the complex [(phen)$_2$Cr(biim)Eu(Tp)$_2$](OTf)$_2$·(CH$_3$CN)$_2$ (thermal ellipsoids are drawn at 50% probability level) with numbering scheme. Triflates counter ions and acetonitrile solvate molecules are omitted for clarity purposes.

Table S7. Crystal data and structure refinement for [(phen)$_2$Cr(biim)Y(Tp)$_2$]·2CH_3CN.

CCDC	2550805	
Empirical formula	C54 H46 B2 Cr F6 N22 O6 S2 Y	
Chemical formula moiety	C48 H40 B2 Cr N20 Y, 2(C F3 O3 S), 2(C2 H3 N)	
Formula weight	1439.78	
Temperature	99.99(11) K	
Wavelength	1.54184 Å	
Crystal system	Triclinic	
Space group	P -1	
Unit cell dimensions	a = 12.79631(15) Å	α= 98.9587(14)°.
	b = 15.6209(3) Å	β= 110.0797(13)°.
	c = 16.9078(3) Å	γ = 101.5791(12)°.
Volume	3015.55(9) Å^3	
Z	2	
Density (calculated)	1.586 Mg/m^3	
Absorption coefficient	4.187 mm^{-1}	
F(000)	1462	
Crystal size	0.148 x 0.022 x 0.009 mm^3	
Theta range for data collection	2.870 to 76.116°.	
Index ranges	-15<=h<=15, -19<=k<=19, -21<=l<=20	
Reflections collected	13905	
Independent reflections	13905 [R(int) = ?]	
Completeness to theta = 67.684°	99.8 %	
Absorption correction	Analytical	
Max. and min. transmission	0.964 and 0.738	
Refinement method	Full-matrix least-squares on F^2	
Data / restraints / parameters	13905 / 0 / 850	
Goodness-of-fit on F^2	1.045	
Final R indices [I>2sigma(I)]	R1 = 0.0643, wR2 = 0.1708	
R indices (all data)	R1 = 0.0682, wR2 = 0.1742	
Extinction coefficient	n/a	
Largest diff. peak and hole	1.925 and -1.398 e.Å^{-3}	

Comments on the crystal structure:

All crystals have thin needle habits and were non-merohedral twins. Integration and structure refinement were performed on two components twinned data (merged hklf5 data) with refined twin fraction (BASF) = 0.5875(13)/04125(13)).

Table S8. Crystal data and structure refinement for $[(phen)_2Cr(biim)Eu(Tp)_2]\cdot 2CH_3CN$.

CCDC	2550806	
Empirical formula	C54 H46 B2 Cr Eu F6 N22 O6 S2	
Chemical formula moiety	C48 H40 B2 Cr Eu N20, 2(C F3 O3 S), 2(C2 H3 N)	
Formula weight	1502.83	
Temperature	100.00(15) K	
Wavelength	1.54184 Å	
Crystal system	Triclinic	
Space group	P -1	
Unit cell dimensions	a = 12.80809(14) Å	α= 98.7930(13)°.
	b = 15.5517(3) Å	β= 109.8712(12)°.
	c = 16.9622(3) Å	γ = 101.5256(12)°.
Volume	3022.73(8) $Å^3$	
Z	2	
Density (calculated)	1.651 Mg/m^3	
Absorption coefficient	10.244 mm^{-1}	
F(000)	1510	
Crystal size	0.493 x 0.045 x 0.028 mm^3	
Theta range for data collection	2.853 to 76.057°.	
Index ranges	-16<=h<=16, -19<=k<=19, -21<=l<=20	
Reflections collected	14187	
Independent reflections	14187 [R(int) = ?]	
Completeness to theta = 67.684°	99.8 %	
Absorption correction	Analytical	
Max. and min. transmission	0.776 and 0.191	
Refinement method	Full-matrix least-squares on F^2	
Data / restraints / parameters	14187 / 0 / 850	
Goodness-of-fit on F^2	1.090	
Final R indices [I>2sigma(I)]	R1 = 0.0594, wR2 = 0.1547	
R indices (all data)	R1 = 0.0608, wR2 = 0.1561	
Extinction coefficient	n/a	
Largest diff. peak and hole	2.454 and -1.644 e.$Å^{-3}$	

Comments on the crystal structure:

This Eu-Cr complex is isostructural to its Y-Cr analogue. Needle crystals are non-merohedral twins. Data integration and structure refinement were performed using 2 components twinned crystal (hklf5 format). The proportion of both components was refined to (BASF=) 0.4341(11)/0.5659(11).

Similarly, vapor diffusion of Et_2O into a solution of [(cyclam)Cr(biim)Ln$(Tp)_2$]OTf_2 (Ln = Y in Figure S22; Ln = Eu in Figure S23 and Ln = Er in Figure S24) in acetonitrile lead to the formation of crystals measurable by XRD. All three compounds crystallize with two independent Cr-Ln complexes (labeled A and B) in the asymmetric unit.

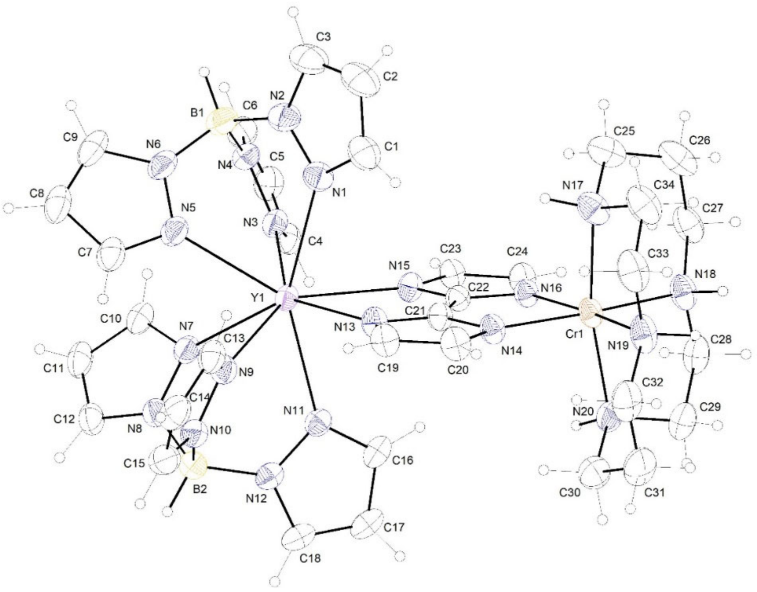

Figure S22. Ortep view of [(cyclam)Cr(biim)Y$(Tp)_2$]$^{2+}$ (complex A) (thermal ellipsoids are drawn at 50% probability) with numbering scheme. Triflate anions and solvent molecules are omitted for clarity.

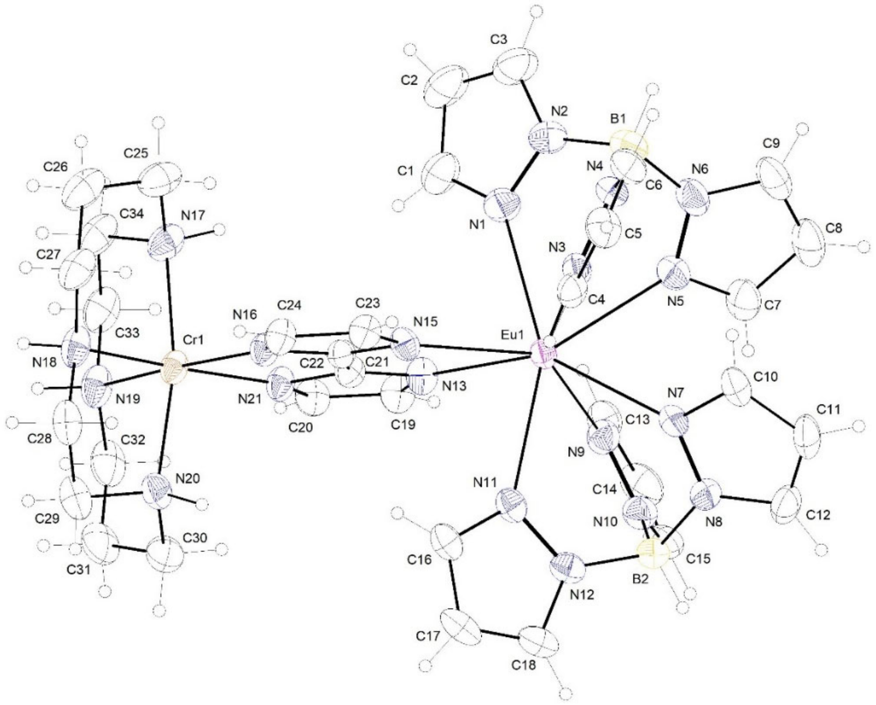

Figure S23. Ortep view of [(cyclam)Cr(biim)Eu(Tp)$_2$]$^{2+}$ (complex A) (thermal ellipsoids are drawn at 50% probability) with numbering scheme. Triflate anions and solvent molecules are omitted for clarity.

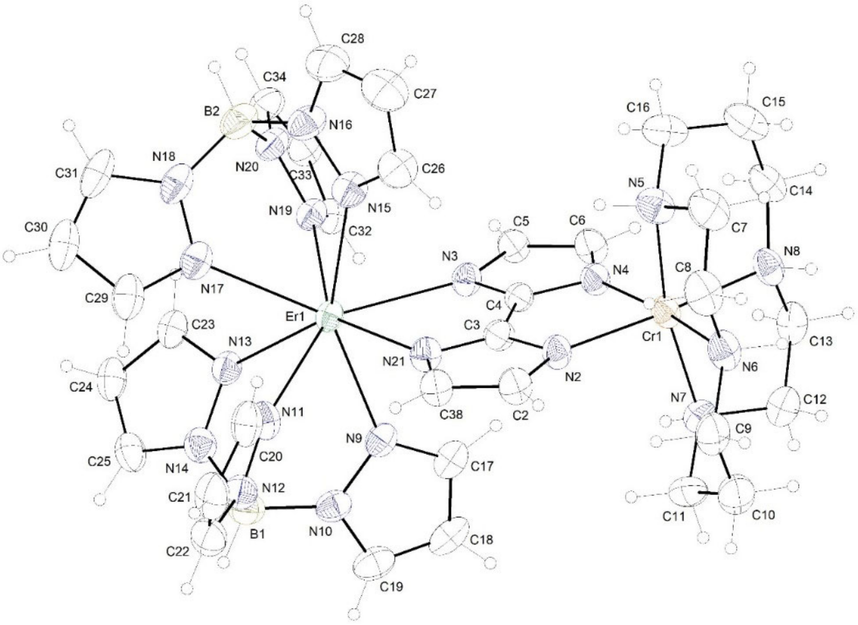

Figure S24. Ortep view of [(cyclam)Cr(biim)Er(Tp)$_2$]$^{2+}$ (complex A) (thermal ellipsoids are drawn at 50% probability) with numbering scheme. Triflate anions and solvent molecules are omitted for clarity.

Table S9. Crystal data and structure refinement for $[(cyclam)Cr(biim)Y(Tp)_2]OTf_2\cdot1.75CH_3CN\cdot0.25CH_4O\cdot0.25H_2O$

CCDC	2550807
Empirical formula	C39.75 H54.75 B2 Cr F6 N21.75 O6.50 S2 Y
Chemical formula moiety	$C_{34}H_{48}B_2CrN_{20}Y$, $2(CF_3O_3S)$, $1.75(C_2H_3N)$, $0.25(CH_4O)$, $0.25(H_2O)$
Formula weight	1281.95
Temperature	120.00(10) K
Wavelength	1.54184 Å
Crystal system	Monoclinic
Space group	$P\ 2_1/n$
Unit cell dimensions	a = 16.02101(8) Å α= 90°.
	b = 29.25948(16) Å β= 104.3954(6)°.
	c = 24.62773(13) Å γ = 90°.
Volume	11182.19(11) $Å^3$
Z	8
Density (calculated)	1.523 Mg/m^3
Absorption coefficient	4.433 mm^{-1}
F(000)	5252
Crystal size	0.49 x 0.03 x 0.02 mm^3
Theta range for data collection	2.390 to 74.314°.
Index ranges	-19<=h<=13, -36<=k<=34, -30<=l<=30
Reflections collected	111056
Independent reflections	22491 [R(int) = 0.0324]
Completeness to theta = 67.684°	100.0 %
Absorption correction	Analytical
Max. and min. transmission	0.927 and 0.410
Refinement method	Full-matrix least-squares on F^2
Data / restraints / parameters	22491 / 2 / 1450
Goodness-of-fit on F^2	1.005
Final R indices [I>2sigma(I)]	R1 = 0.0604, wR2 = 0.1655
R indices (all data)	R1 = 0.0693, wR2 = 0.1719
Extinction coefficient	n/a
Largest diff. peak and hole	1.554 and -1.169 e.$Å^{-3}$

Table S10. Crystal data and structure refinement for [(cyclam)Cr(biim)Eu(Tp)$_2$]OTf$_2$·2.125CH$_3$CN.

CCDC	2550808	
Empirical formula	C40.25 H54.38 B2 Cr Eu F6 N22.12 O6 S2	
Chemical formula moiety	$C_{34}H_{48}B_2CrEuN_{20}$, 2($CF_3O_3S$), 2.125($C_2H_3N$)	
Formula weight	1347.88	
Temperature	119.99(10) K	
Wavelength	1.54184 Å	
Crystal system	Monoclinic	
Space group	P 2_1/n	
Unit cell dimensions	a = 16.03829(9) Å	α= 90°.
	b = 29.26191(17) Å	β= 104.4037(6)°.
	c = 24.71791(15) Å	γ = 90°.
Volume	11235.75(12) Å^3	
Z	8	
Density (calculated)	1.594 Mg/m^3	
Absorption coefficient	10.938 mm^{-1}	
F(000)	5454	
Crystal size	0.54 x 0.03 x 0.03 mm^3	
Theta range for data collection	2.384 to 74.313°.	
Index ranges	-11<=h<=19, -36<=k<=36, -30<=l<=28	
Reflections collected	111693	
Independent reflections	22513 [R(int) = 0.0437]	
Completeness to theta = 67.684°	99.7 %	
Absorption correction	Analytical	
Max. and min. transmission	0.769 and 0.153	
Refinement method	Full-matrix least-squares on F^2	
Data / restraints / parameters	22513 / 2 / 1433	
Goodness-of-fit on F^2	1.027	
Final R indices [I>2sigma(I)]	R1 = 0.0536, wR2 = 0.1382	
R indices (all data)	R1 = 0.0642, wR2 = 0.1444	
Extinction coefficient	n/a	
Largest diff. peak and hole	1.386 and -1.038 e.Å^{-3}	

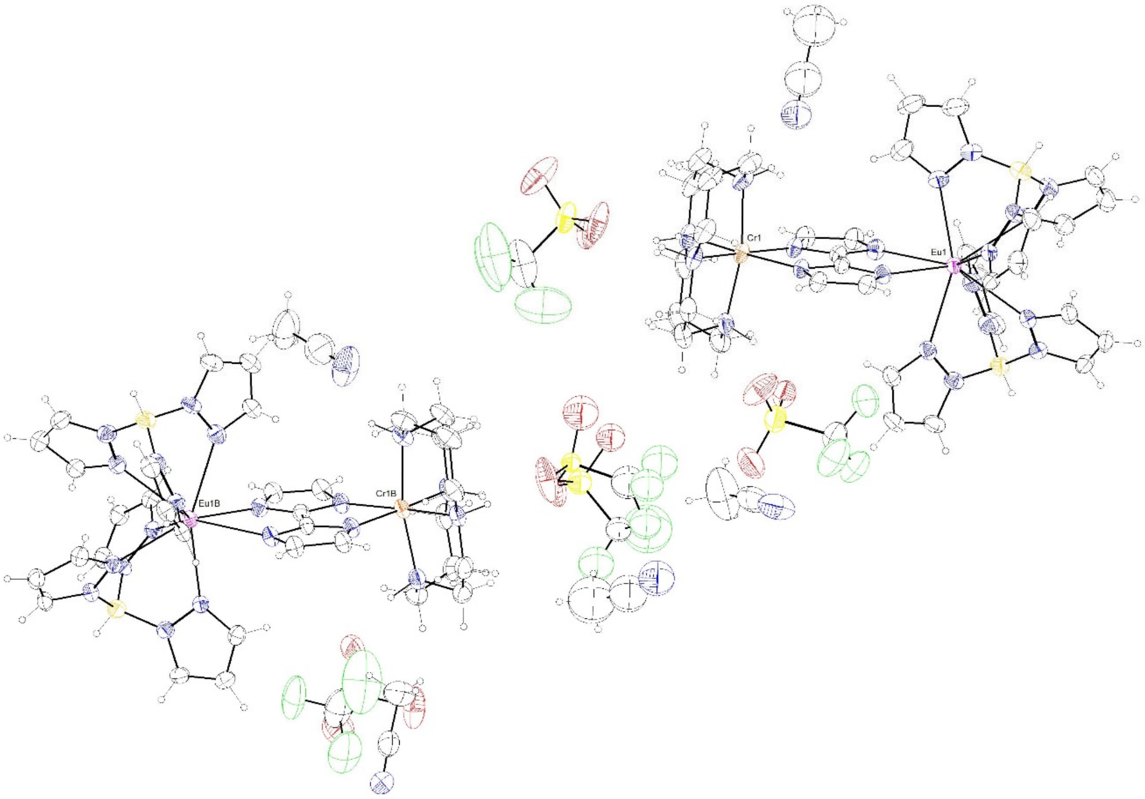

Figure S25. Ortep view of the asymmetric unit of [(cyclam)Cr(biim)Eu(Tp)$_2$]OTf$_2$ with anions and solvent molecules included.

Table S11. Crystal data and structure refinement for $[(cyclam)Cr(biim)Er(Tp)_2]OTf_2 \cdot 2.125CH_3CN \cdot 0.125CH_2Cl_2 \cdot 0.125H_2O$.

CCDC	2550809
Empirical formula	C40.38 H54.88 B2 Cl0.25 Cr Er F6 N22.12 O6.12 S2
Chemical formula moiety	$C_{34}H_{48}B_2CrErN_{20}$, $2(CF_3O_3S)$, $2.125(C_2H_3N)$, $0.125(CH_2Cl_2)$, $0.125(H_2O)$
Formula weight	1376.05
Temperature	119.98(10) K
Wavelength	1.54184 Å
Crystal system	Monoclinic
Space group	P 2_1/n
Unit cell dimensions	a = 16.01463(8) Å α= 90°.
	b = 29.30906(18) Å β= 104.1111(6)°.
	c = 24.63867(15) Å γ = 90°.
Volume	11215.77(12) $Å^3$
Z	8
Density (calculated)	1.630 Mg/m^3
Absorption coefficient	5.844 mm^{-1}
F(000)	5546
Crystal size	0.64 x 0.04 x 0.02 mm^3
Theta range for data collection	2.386 to 74.360°.
Index ranges	-16<=h<=19, -35<=k<=36, -30<=l<=30
Reflections collected	116157
Independent reflections	22573 [R(int) = 0.0339]
Completeness to theta = 67.684°	99.9 %
Absorption correction	Analytical
Max. and min. transmission	0.885 and 0.260
Refinement method	Full-matrix least-squares on F^2
Data / restraints / parameters	22573 / 27 / 1449
Goodness-of-fit on F^2	1.029
Final R indices [I>2sigma(I)]	R_1 = 0.0588, wR_2 = 0.1552
R indices (all data)	R_1 = 0.0679, wR_2 = 0.1617
Extinction coefficient	n/a
Largest diff. peak and hole	1.719 and -1.634 e.$Å^{-3}$

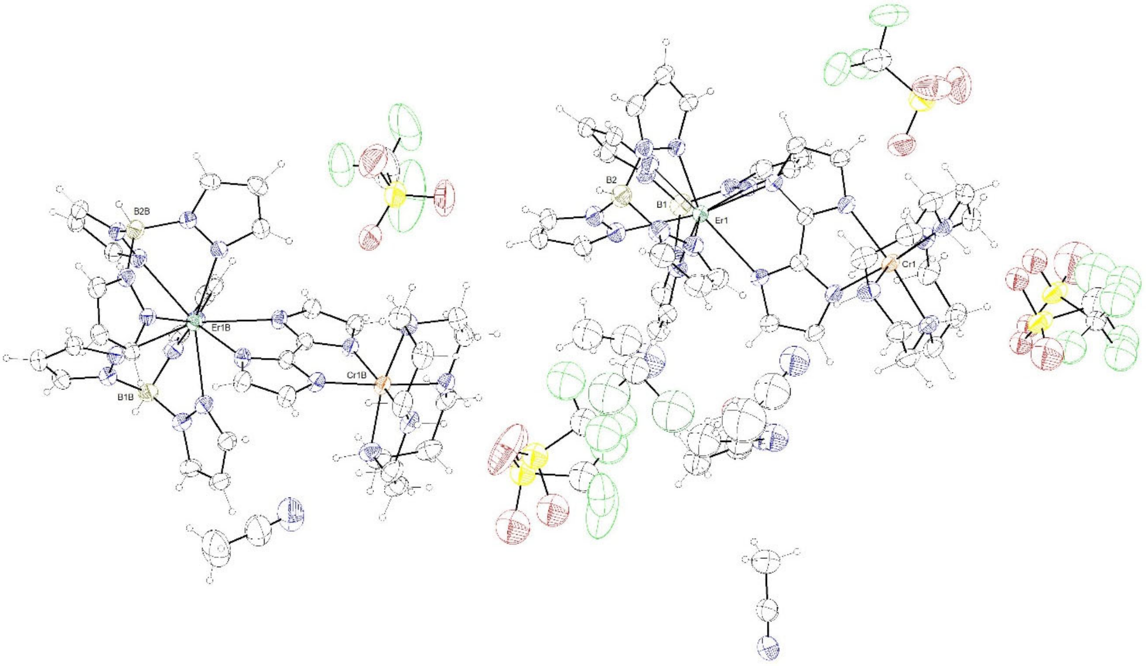

Figure S26. Asymmetric unit in the crystal structure of [(cyclam)Cr(biim)Er(Tp)$_2$]OTf$_2$ including triflates and solvent molecules.

A3.3. Crystal structures of the mononuclear heteroleptic complexes [Ln(Tp)₂(Me₂biim)]OTf.

The structure of the [Ln(Tp)$_2$(Me$_2$biim)]OTf complexes (Ln = Y in Figure S27 and Ln = Er in Figure S28) was obtained by vapor diffusion of pentane into a THF solution of complex, giving two isostructural block-shaped crystals.

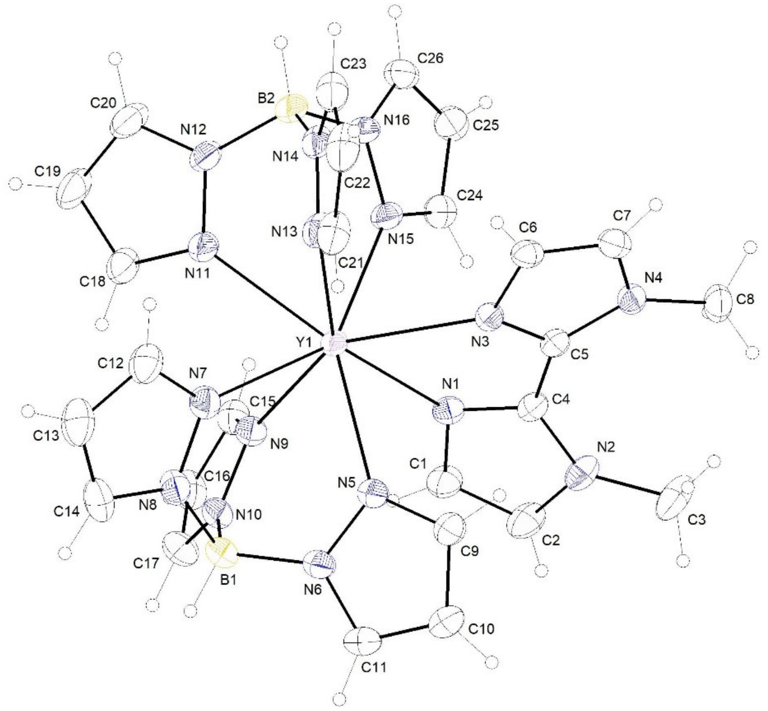

Figure S27. Ortep view of [Y(Tp)$_2$(Me$_2$biim)]$^+$ (thermal ellipsoids are drawn at 50% probability) with numbering scheme. Triflate anion is omitted for clarity.

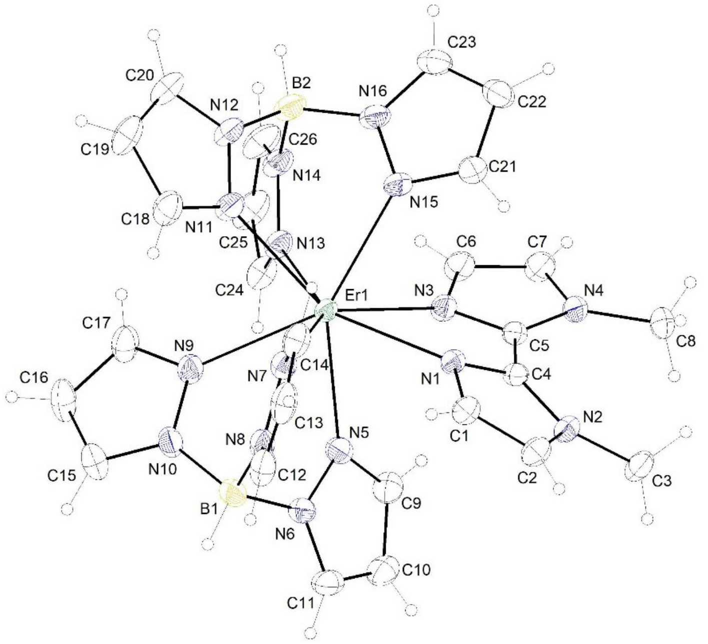

Figure S28. Ortep view of [Er(Tp)$_2$(Me$_2$biim)]$^+$ (thermal ellipsoids are drawn at 50% probability) with numbering scheme. Triflate anion is omitted for clarity.

Table S12. Crystal data and structure refinement for $[Y(Tp)_2(Me_2biim)]OTf$.

CCDC	2550810	
Empirical formula	C27 H30 B2 F3 N16 O3 S Y	
Chemical formula moiety	C26 H30 B2 N16 Y, C F3 O3 S	
Formula weight	826.26	
Temperature	119.99(10) K	
Wavelength	1.54184 Å	
Crystal system	Monoclinic	
Space group	$P\ 2_1/c$	
Unit cell dimensions	a = 8.37507(6) Å	a= 90°.
	b = 20.51570(16) Å	b= 90.6903(7)°.
	c = 20.56304(16) Å	g = 90°.
Volume	3532.89(5) $Å^3$	
Z	4	
Density (calculated)	1.553 Mg/m^3	
Absorption coefficient	3.507 mm^{-1}	
F(000)	1680	
Crystal size	0.39 x 0.2 x 0.12 mm^3	
Theta range for data collection	3.043 to 74.351°.	
Index ranges	-9<=h<=10, -25<=k<=25, -25<=l<=25	
Reflections collected	34932	
Independent reflections	7132 [R(int) = 0.0402]	
Completeness to theta = 67.684°	100.0 %	
Absorption correction	Analytical	
Max. and min. transmission	0.705 and 0.405	
Refinement method	Full-matrix least-squares on F^2	
Data / restraints / parameters	7132 / 18 / 517	
Goodness-of-fit on F^2	1.038	
Final R indices [I>2sigma(I)]	R1 = 0.0341, wR2 = 0.0930	
R indices (all data)	R1 = 0.0350, wR2 = 0.0938	
Extinction coefficient	n/a	
Largest diff. peak and hole	0.949 and -0.765 $e.Å^{-3}$	

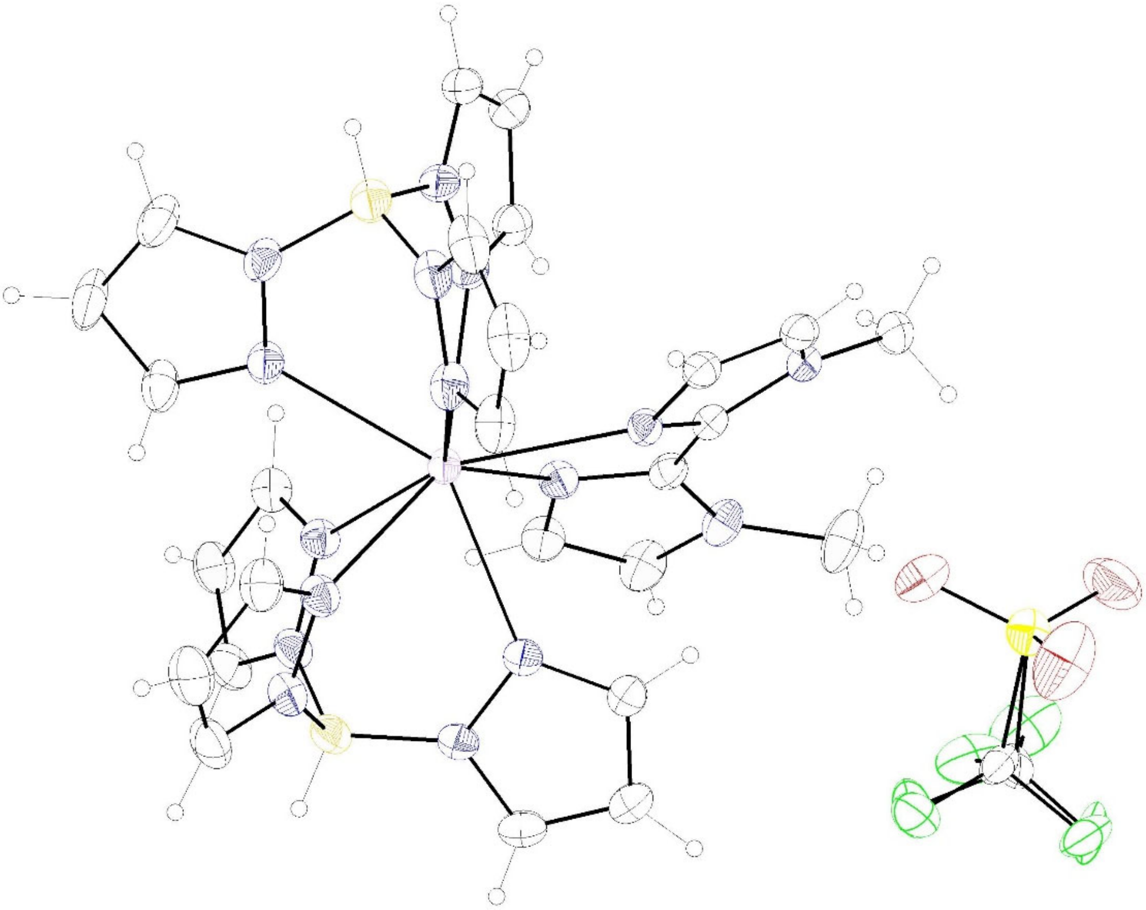

Figure S29. Molecular view of the asymmetric unit in the crystal of $[Y(Tp)_2(Me_2biim)]OTf$ (thermal ellipsoids are drawn at 50% probability level). Triflate ion is slightly disordered.

Table S13. Crystal data and structure refinement for $[Er(Tp)_2(Me_2biim)]OTf$.

CCDC	2550811	
Empirical formula	C27 H30 B2 Er F3 N16 O3 S	
Chemical formula moiety	C26 H30 B2 Er N16, C F3 O3 S	
Formula weight	904.61	
Temperature	120.00(10) K	
Wavelength	1.54184 Å	
Crystal system	Monoclinic	
Space group	P 2_1/c	
Unit cell dimensions	a = 8.09643(3) Å	α= 90°.
	b = 20.59536(9) Å	β= 90.6333(4)°.
	c = 21.01723(8) Å	γ = 90°.
Volume	3504.38(2) $Å^3$	
Z	4	
Density (calculated)	1.715 Mg/m^3	
Absorption coefficient	5.636 mm^{-1}	
F(000)	1796	
Crystal size	0.2 x 0.17 x 0.07 mm^3	
Theta range for data collection	3.004 to 76.038°.	
Index ranges	-9<=h<=10, -25<=k<=25, -26<=l<=17	
Reflections collected	59768	
Independent reflections	7168 [R(int) = 0.0217]	
Completeness to theta = 67.684°	100.0 %	
Absorption correction	Analytical	
Max. and min. transmission	0.839 and 0.644	
Refinement method	Full-matrix least-squares on F^2	
Data / restraints / parameters	7168 / 1 / 499	
Goodness-of-fit on F^2	1.071	
Final R indices [I>2sigma(I)]	R1 = 0.0265, wR2 = 0.0635	
R indices (all data)	R1 = 0.0267, wR2 = 0.0636	
Extinction coefficient	n/a	
Largest diff. peak and hole	1.049 and -0.830 e.$Å^{-3}$	

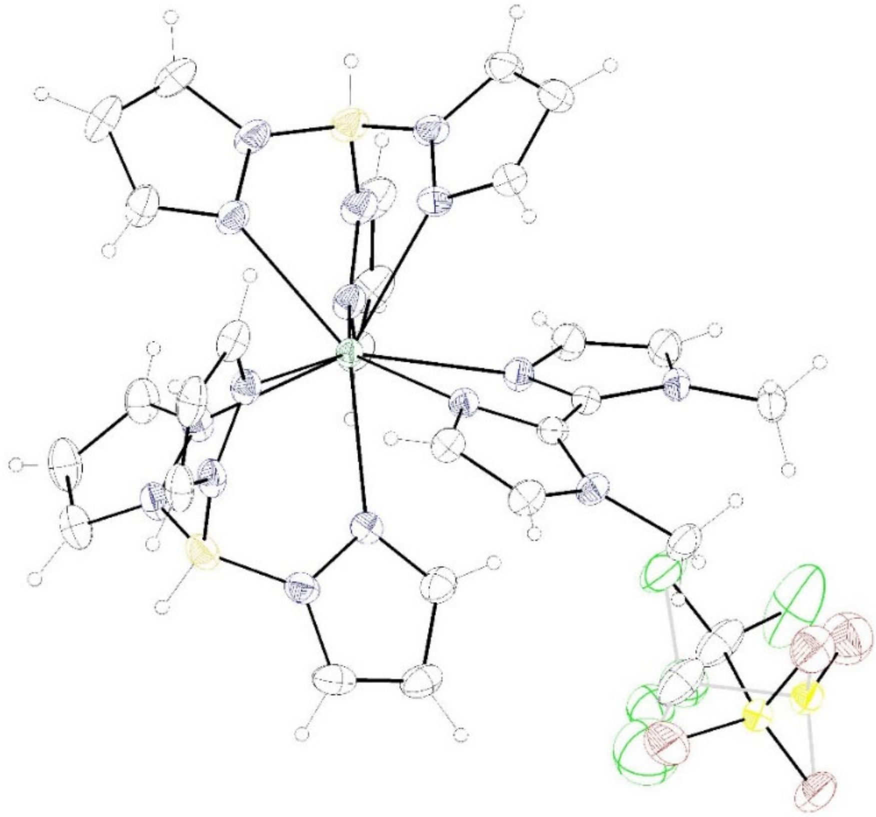

Figure S30. View of the asymmetric unit in crystal of [Er(Tp)$_2$(Me$_2$biim)]OTf (Thermal ellipsoids are drawn at 50% probability level). Triflate ion is disordered on two positions.

A3.4. Analysis of the crystal structures

For all the Cr-Ln and for $[Ln(Tp)_2(Me_2biim)]^+$ complexes, the lanthanide cation is surrounded by eight nitrogen atoms in the first coordination sphere, two from the biimidazolate (or Me_2biim) and three from each Tp^- ligands. The closest geometry of the first coordination sphere was determined using continuous shape measurements [A3-1],[A3-2] (abbreviated as CShM) and the software SHAPE [A3-3]. CShM is defined as the distance to an ideal shape, independent of size and orientation. For the first coordination sphere of the lanthanide, the coordinates of the eight coordinating atoms are given by their position vectors $\overrightarrow{Q_k}$ (k = 1, 2, ···, 8), whereas the coordinates for a given geometry closest in size and orientation is given by the vectors $\overrightarrow{P_k}$(k = 1, 2, ···, 8). The distance $S_Q(P)$ of the molecular structure Q to the perfect polyhedron P is then defined as:

$$S_Q(P) = \min \frac{\sum_{k=1}^{N} \left|\overrightarrow{Q_k} - \overrightarrow{P_k}\right|^2}{\sum_{k=1}^{N} \left|\overrightarrow{Q_k} - \overrightarrow{Q_0}\right|^2} \times 100 \qquad \text{(A3-1)}$$

where $\overrightarrow{Q_0}$ is the coordinate vector of the geometrical center of the investigated structure. With this equation, we obtain $0 < S_Q(P) < 100$. The closer $S_Q(P)$ is to 0, the closer to the given geometry is the analyzed structure, whereas if the coordination sphere is more distorted, $S_Q(P)$ increases. In this case, the CShM was compared with 13 known geometries for eight-coordinate centers, and it was found the closest geometry is the square antiprism (ideal symmetry point group D_{4d}, Figure S31 and Table S14).

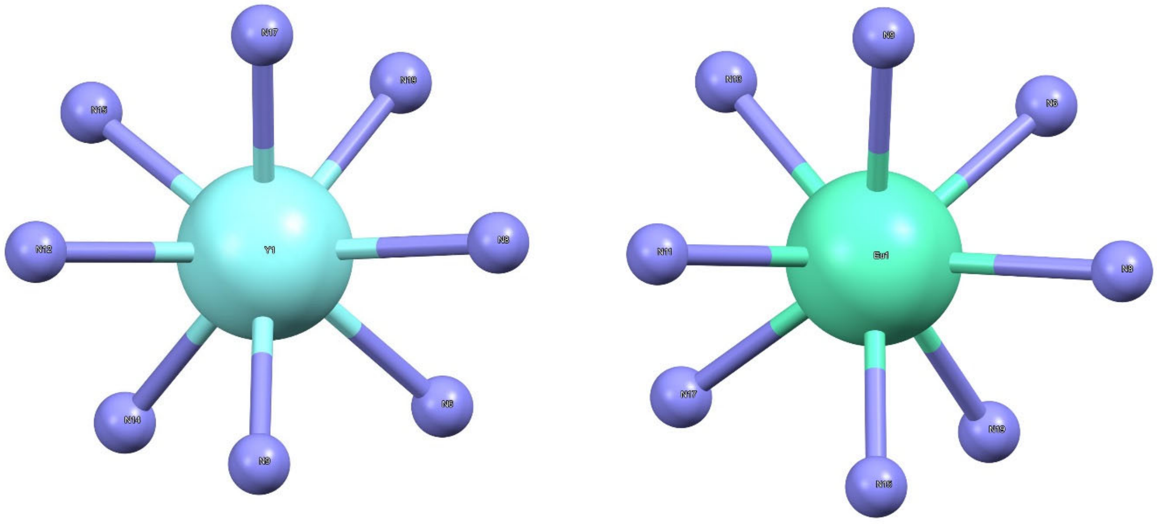

Figure S31. Structure of the first coordination sphere LnN_8 in the crystal structure of $[(phen)_2Cr(biim)Y(Tp)_2](OTf)_2$ (left) and $[(phen)_2Cr(biim)Eu(Tp)_2](OTf)_2$ (right), highlighting the square antiprism geometry of the nitrogen atoms around the Ln(III) cation. N6 and N8 are the nitrogen atoms of the $biim^{2-}$ ligand while the other N atoms are from the Tp^- ligands.

Table S14. Average Ln-N in the first coordination sphere of the Ln^{3+} cation, Ln-Cr distance in the crystal structures of $[(phen)_2Cr(biim)Ln(Tp)_2](OTf)_2$, and $S_Q(P)$ value for compared to square antiprism geometry.

Complex	$S_Q(P)$ for square antiprism geometry	Average Ln-N_{Tp} distance / Å	Average Ln-N_{biim} distance / Å
$[(phen)_2Cr(biim)Y(Tp)_2]^{2+}$	0.293	2.47(4)	2.55(1)
$[(phen)_2Cr(biim)Eu(Tp)_2]^{2+}$	0.311	2.51(4)	2.592(3)
$[(cyclam)Cr(biim)Y(Tp)_2]^{2+}$	0.5(1)	2.48(4)	2.52(1)
$[(cyclam)Cr(biim)Eu(Tp)_2]^{2+}$	0.5(1)	2.52(4)	2.56(1)
$[(cyclam)Cr(biim)Er(Tp)_2]^{2+}$	0.5(1)	2.47(4)	2.51(2)
$[Y(Tp)_2(Me_2biim)]^{+}$	0.997	2.47(4)	2.498(9)
$[Er(Tp)_2(Me_2biim)]^{+}$	1.354	2.45(3)	2.498(1)

The Ln-N bond length decreases slightly along Eu > Y > Er in agreement with the standard lanthanide contraction series. The Ln-N distance is slightly shorter with the Tp^- ligand than with the biimidazolate. The Ln···Cr distance of these two complexes is compared to other Ln-Cr assemblies reported in literature in Table S15.

Table S15. Ln-Cr distance in the crystal structure of a selection of heterometallic Ln-Cr complexes reported in literature. Py-bzimpy denotes the ligand 2-{6-[N,N-diethylcarboxamido]pyridin-2-yl}-1,1'-dimethyl-5,5'methylene-2'-(5-methylpyridin-2-yl)bis[1H-benzimidazole].

Complex	Ln-Cr distance / Å	Reference
$[(phen)_2Cr(biim)Y(Tp)_2]^{2+}$	5.8464(7)	This work
$[(phen)_2Cr(biim)Eu(Tp)_2]^{2+}$	5.879(1)	This work
$[(cyclam)Cr(biim)Y(Tp)_2]^{2+}$	5.889(1)	This work
$[(cyclam)Cr(biim)Eu(Tp)_2]^{2+}$	5.924(4)	This work
$[(cyclam)Cr(biim)Er(Tp)_2]^{2+}$	5.877(3)	This work
$[(acac)_2Cr(ox)Yb(Tp)_2]^{2+}$	5.631	[A3-4]
$[EuCr(py\text{-}bzimpy)_3]^{6+}$	9.324(1)	[A3-5]
$[(acac)_2Cr(bpypz)Yb(hfac)_3]$	4.6508(8)	[A3-6]
$[Cr(CN)_6Yb(DMF)_4(H_2O)_2]$	5.5863(7)	[A3-7]
trans-$[(py)_4CrF_2Gd(hfac)_4]$	4.280(6)	[A3-8]

The biimidazolate bridging ligand offers a significantly shorter Ln···Cr distance than in the triple helical complexes $[LnCr(py\text{-}bzimpy)_3]^{6+}$, but a similar Ln···Cr distance previously reported for

heterometallic complexes bridged by oxalate or cyanide ligands. This short intermetallic distance appears as an advantage to optimize the energy transfer or magnetic coupling between two metal centers.

On the chromium side, the six nitrogen atoms adopt a pseudo-octahedral geometry. The deviation from perfect octahedron was calculated according to the parameter $S_Q(P)$ discussed above, and Σ [A3-9] which is derived with equation (A3-2) and collected in Table S16.

$$\Sigma = \sum_{i=1}^{12} |\phi_i - 90| \qquad \text{(A3-2)}$$

where each Φ_i is one of the 12 N-Cr-N' cisoid angles.

Table S16. Average Cr-N in the first coordination sphere of the Cr^{3+} cation and deviation from the octahedral geometry represented by the parameters Σ and S_Q(P) for the octahedron.

Complex	Σ /°	$S_Q(P)$ for octahedral geometry	Average Cr-$N_{phen/cyclam}$ distance / Å	Average Cr-N_{biim} distance / Å
$[Cr(phen)_2(H_2biim)]^{3+}$	56.5	0.713	2.054(2)	2.024(7)
$[Cr(phen)_2(Hbiim)]^{2+}$	57.3	0.685	2.062(6)	2.018(4)
$[Cr(phen)_2(biim)]^{+}$	58.5	0.804	2.071(10)	1.997(5)
$[(phen)_2Cr(biim)Y(Tp)_2]^{2+}$	55.9	0.711	2.058(4)	2.041(5)
$[(phen)_2Cr(biim)Eu(Tp)_2]^{2+}$	56.0	0.714	2.064(9)	2.041(1)
$[Cr(cyclam)(H_2biim)]^{3+}$	49.5	0.62(3)	2.080(8)	2.093(6)
$[Cr(cyclam)(Hbiim)]^{2+}$	48.6	0.612	2.089(8)	2.07(2)
$[(cyclam)Cr(biim)Y(Tp)_2]^{2+}$	49.3	0.64(4)	2.086(9)	2.088(7)
$[(cyclam)Cr(biim)Eu(Tp)_2]^{2+}$	49.5	0.64(3)	2.085(9)	2.083(5)
$[(cyclam)Cr(biim)Er(Tp)_2]^{2+}$	48.7	0.63(1)	2.085(9)	2.088(10)

The distortion from the octahedral geometry of the coordination sphere around the chromium is similar in the heterometallic assemblies as in their corresponding mononuclear Cr(III) complexes. However, we notice that complexes with cyclam are less distorted than those with phen despite the similar formation of two five-membered chelate rings.

For the complexes $[(phen)_2Cr(biim)Ln(Tp)_2]^{2+}$, the Cr-$N_{biim}$ distance with the biimidazolate ligand is increased upon coordination to the Ln moiety, while the Cr-N_{phen} remains constant. This is the direct consequence of adding an electro-attracting group on the biimidazolate which reduces the negative charge density on this ligand and reduces its affinity toward the positively charged Cr^{3+}. A similar effect is observed upon the addition of protons instead of a metal as discussed in reference [64].

A3.5 References

A3-1 Pinsky, M.; Avnir, D. Continuous Symmetry Measures. 5. The Classical Polyhedra. *Inorg. Chem.* **1998**, *37*, 5575–5582.

A3-2 Casanova, D.; Cirera, J.; Llunell, M.; Alemany, P.; Avnir, D.; Alvarez, S. Minimal Distortion Pathways in Polyhedral Rearrangements. *J. Am. Chem. Soc.* **2004**, *126*, 1755–1763.

A3-3 Stereochemical Analysis of Molecular Fragments | IQTC - The Institute of Theoretical and Computational Chemistry of the Universitat de Barcelona. https://www.iqtc.ub.edu/uncategorised/program-for-the-stereochemical-analysis-of-molecular-fragments-by-means-of-continous-shape-measures-and-associated-tools/ (accessed 2023-03-17).

A3-4 Sanada, T.; Suzuki, T.; Yoshida, T.; Kaizaki, S. Heterodinuclear Complexes Containing D- and f-Block Elements: Synthesis, Structural Characterization, and Metal−Metal Interactions of Novel Chromium(III)−Lanthanide(III) Compounds Bridged by Oxalate. *Inorg. Chem.* **1998**, *37*, 4712–4717.

A3-5 Cantuel, M.; Bernardinelli, G.; Imbert, D.; G. Bünzli, J.-C.; Hopfgartner, G.; Piguet, C. A Kinetically Inert and Optically Active Cr III Partner in Thermodynamically Self-Assembled Heterodimetallic Non-Covalent d–f Podates. *J. Chem. Soc. Dalton Trans.* **2002**, 1929–1940.

A3-6 Kawahata, R.; Tsukuda, T.; Yagi, T.; Subhan, M. A.; Nakata, H.; Fuyuhiro, A.; Kaizaki, S. Novel Structural Transformation around Ln(III) and Unusual Bending of Acetylacetonato Chelate in A Series of New 3d–4f Dinuclear Complexes [(Hfac)3Ln(μ-Bpypz)Cr(Acac)2]. *Chem. Lett.* **2003**, *32*, 1084–1085.

A3-7 Lazarides, T.; M. Davies, G.; Adams, H.; Sabatini, C.; Barigelletti, F.; Barbieri, A.; A. Pope, S. J.; Faulkner, S.; D. Ward, M. Ligand -Field Excited States of Hexacyanochromate and Hexacyanocobaltate as Sensitisers for Near-Infrared Luminescence from Nd(III) and Yb(III) in Cyanide-Bridged d–f Assemblies. *Photochem. Photobiol. Sci.* **2007**, *6*, 1152–1157.

A3-8 Kumar Singh, S.; S. Pedersen, K.; Sigrist, M.; Aa. Thuesen, C.; Schau-Magnussen, M.; Mutka, H.; Piligkos, S.; Weihe, H.; Rajaraman, G.; Bendix, J. Angular Dependence of the Exchange Interaction in Fluoride-Bridged Gd^{III}–Cr^{III} Complexes. *Chem. Commun.* **2013**, *49*, 5583–5585.

A3-9 Ketkaew, R.; Tantirungrotechai, Y.; Harding, P.; Chastanet, G.; Guionneau, P.; Marchivie, M.; J. Harding, D. OctaDist: A Tool for Calculating Distortion Parameters in Spin Crossover and Coordination Complexes. *Dalton Trans.* **2021**, *50*, 1086–1096.

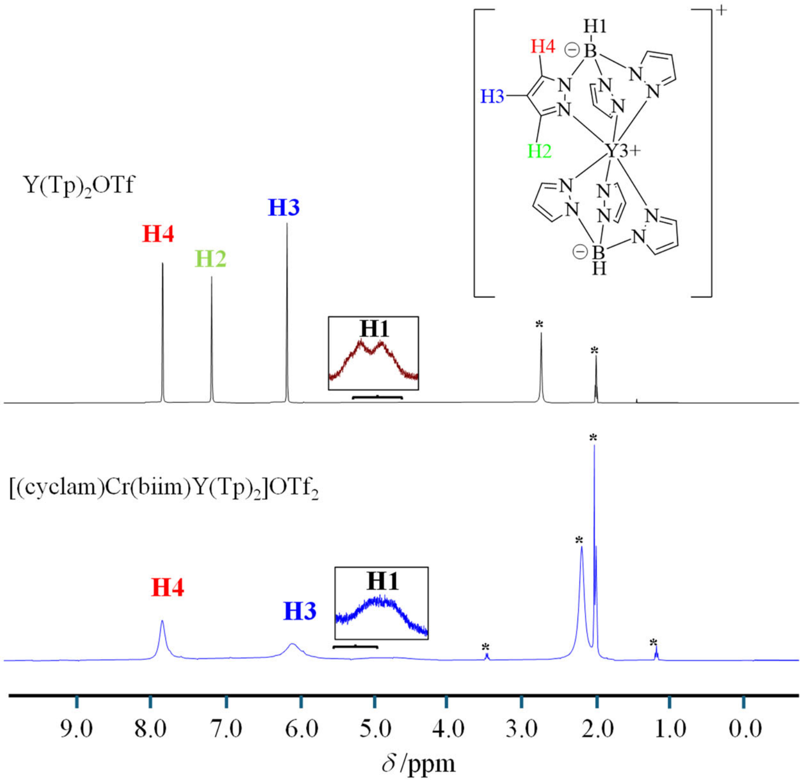

Figure S32. Stacked ^{1}H-NMR spectra in CD_3CN of: $Y(Tp)_2(OTf)$ (top) and $[(cyclam)Cr(biim)Y(Tp)_2]OTf_2$ (bottom). * denotes the solvents signals of CHD_2CN, H_2O and residual traces of Et_2O from the synthesis.

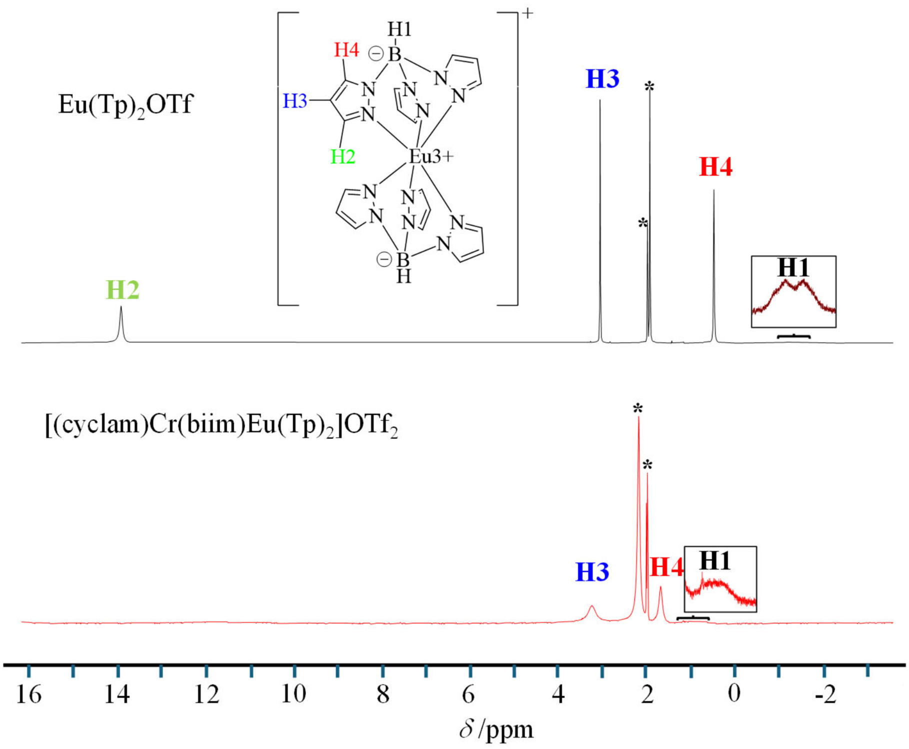

Figure S33. Stacked ^{1}H-NMR spectra in CD_3CN of: $Eu(Tp)_2(OTf)$ (top) and $[(cyclam)Cr(biim)Eu(Tp)_2]OTf_2$ (bottom). * denotes the solvents signals of CHD_2CN, H_2O and residual traces of CH_3CN from the synthesis.

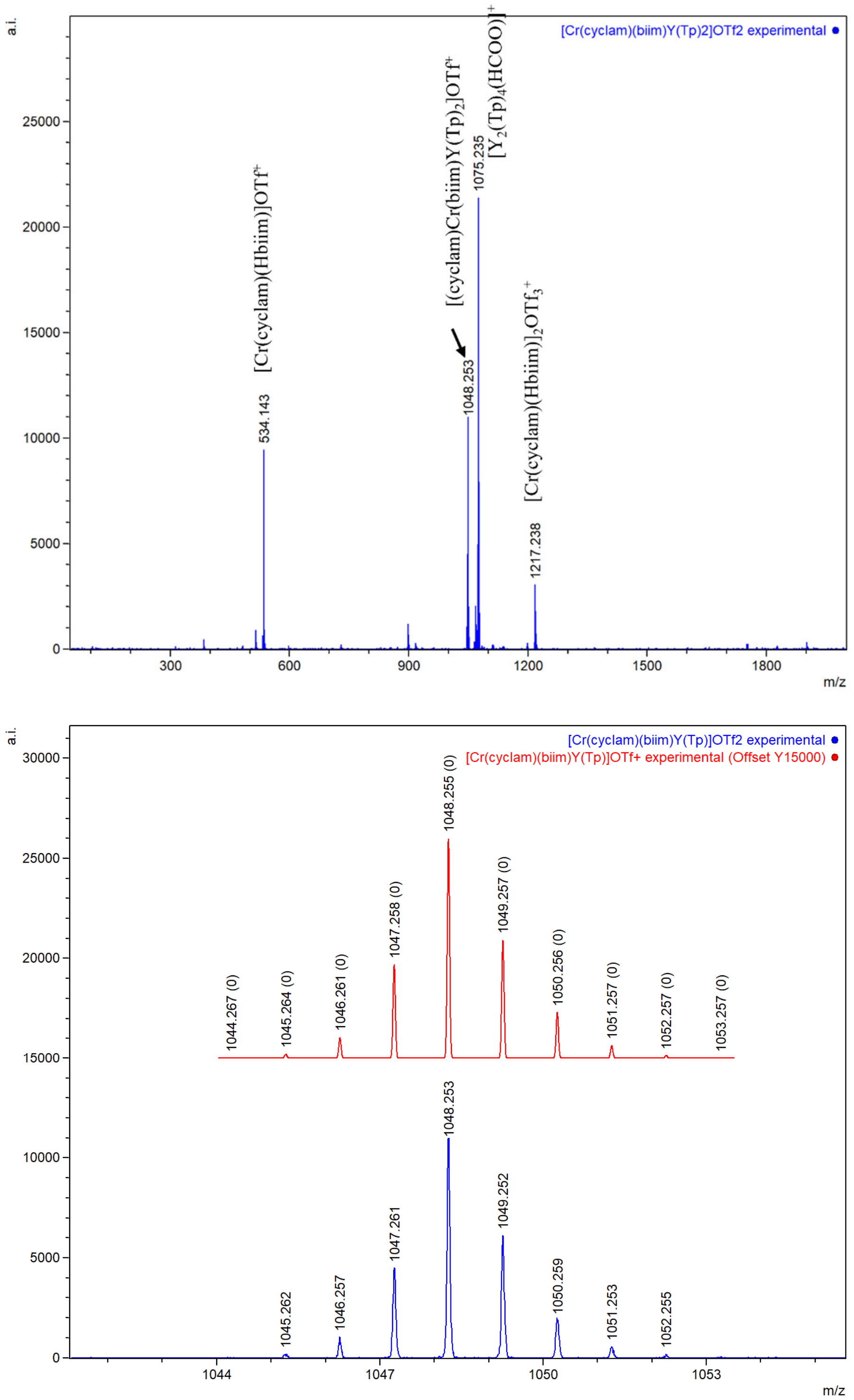

Figure S34. Top: ESI-HRMS full spectrum of a solution of [(cyclam)Cr(biim)Y(Tp)2]OTf2; solvent: CH_3CN; c = 5·10^{-3}M. Bottom: Zoom on the experimental HRMS spectrum (blue) and calculated signal of [(cyclam)Cr(biim)Y(Tp)2]OTf$^+$ (red).

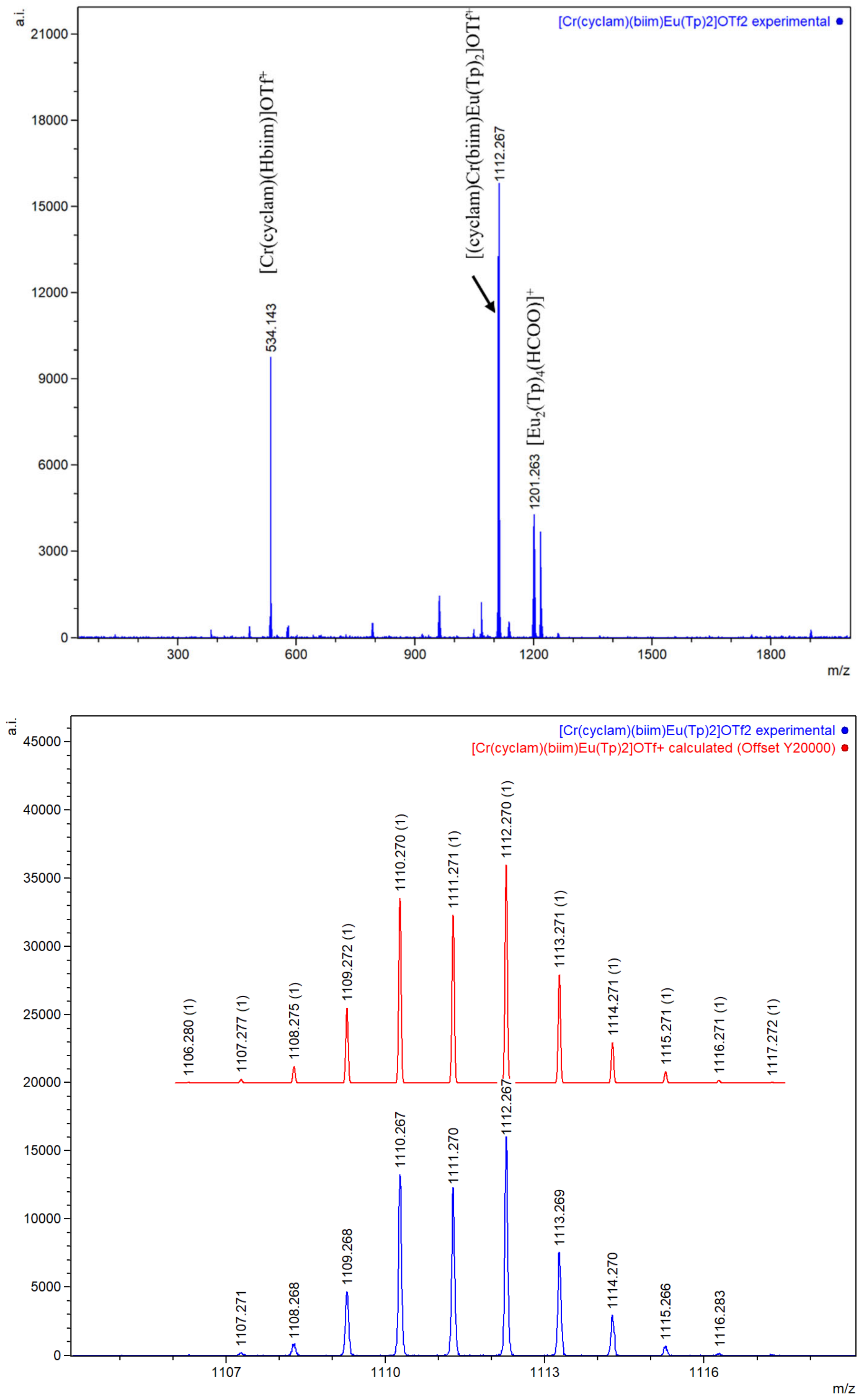

Figure S35. Top: ESI-HRMS full spectrum of a solution of [(cyclam)Cr(biim)Eu(Tp)$_2$]OTf$_2$; solvent: CH_3CN; c = $5\cdot10^{-3}$M. Bottom: Zoom on the experimental HRMS spectrum (blue) and calculated signal of [(cyclam)Cr(biim)Eu(Tp)$_2$]OTf$^+$ (red).

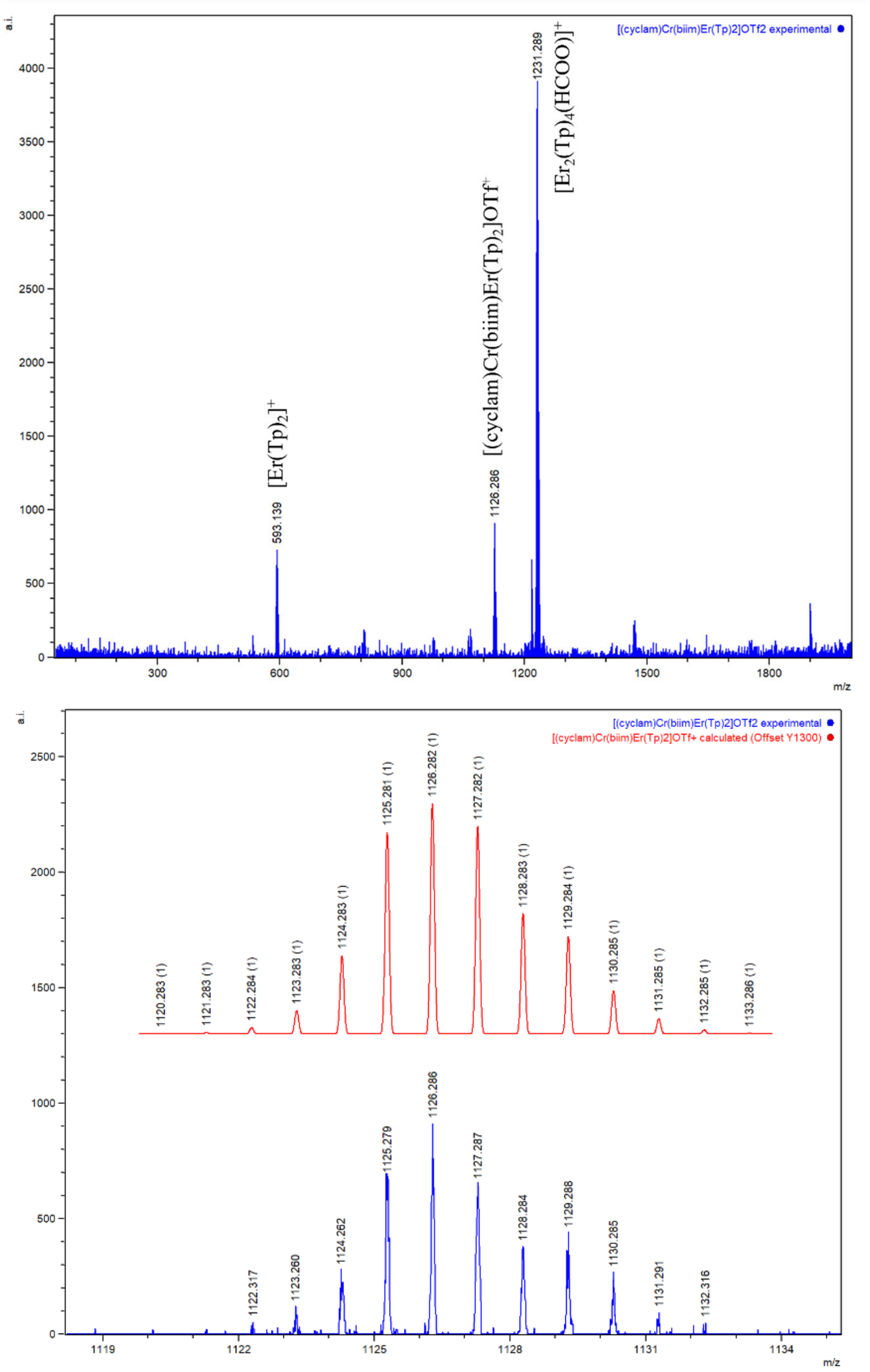

Figure S36. Top: ESI-HRMS full spectrum of a solution of [(cyclam)Cr(biim)Er(Tp)$_2$]OTf$_2$; solvent: CH_3CN; c = 5·10^{-3}M. Bottom: Zoom on the experimental HRMS spectrum (blue) and calculated signal of [(cyclam)Cr(biim)Er(Tp)$_2$]OTf$^+$ (red). The presence of the peak for [Ln$_2$(Tp)$_4$(HCOO)]$^+$ in the MS is due to the presence of small quantities (0.1%) of formic acid added in the solvent used in HRMS to help to ionize organic molecules in solution. The presence of this peak does not reflect the real presence of this dimer in solution outside of the MS conditions

Supplementary File S4. Characterization of $[(Me_2biim)Ln(Tp)_2]^+$ (Ln = Y, Er) in solution.

The ^{1}H-NMR spectrum of $[(Me_2biim)Y(Tp)_2]OTf$ was recorded in CD_3CN (Figure S37). While the signals of H^1, H^3 and H^4 are not affected by the complexation of Me2biim, the signals of H^2, H^5, H^6 and H^7 appear shifted and broadened after complexation. This testifies that Me2biim coordinates to $[Y(Tp)_2]^+$.

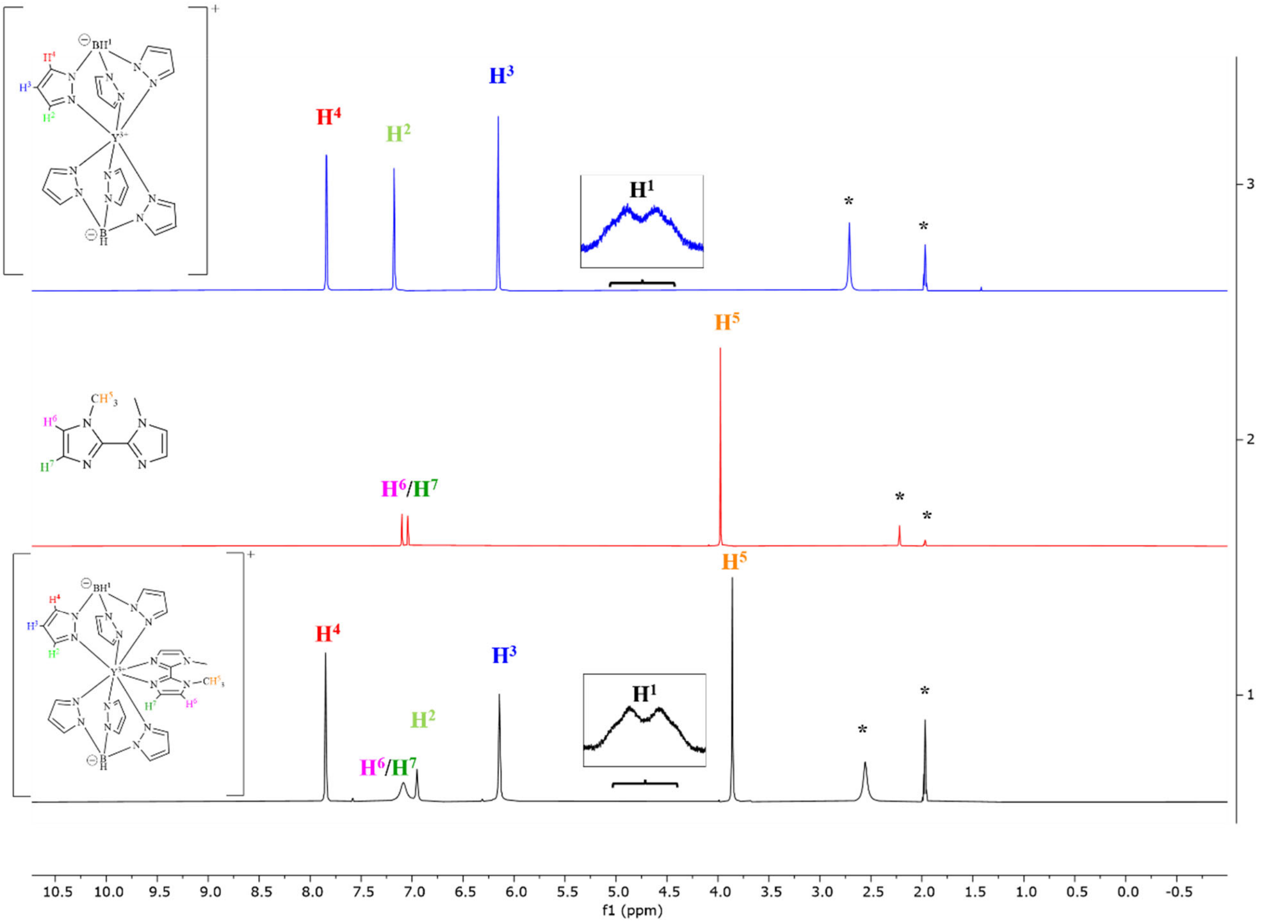

Figure S37. Stacked ^{1}H-NMR spectra in CD_3CN of $Y(Tp)_2(OTf)$ (top), Me2biim (middle) and $[(Me_2biim)Y(Tp)_2]OTf$ (bottom). * denotes the solvents signals of CHD_2CN and H_2O.

In addition, the HRMS spectra of solutions of [(Me_2biim)Ln($Tp)_2$]OTf (Ln = Y, Figure S38 and Ln = Er, Figure S39) display the peaks of [(Me_2biim)Ln($Tp)_2$]$^+$ confirming the formation of the target complexes in solution.

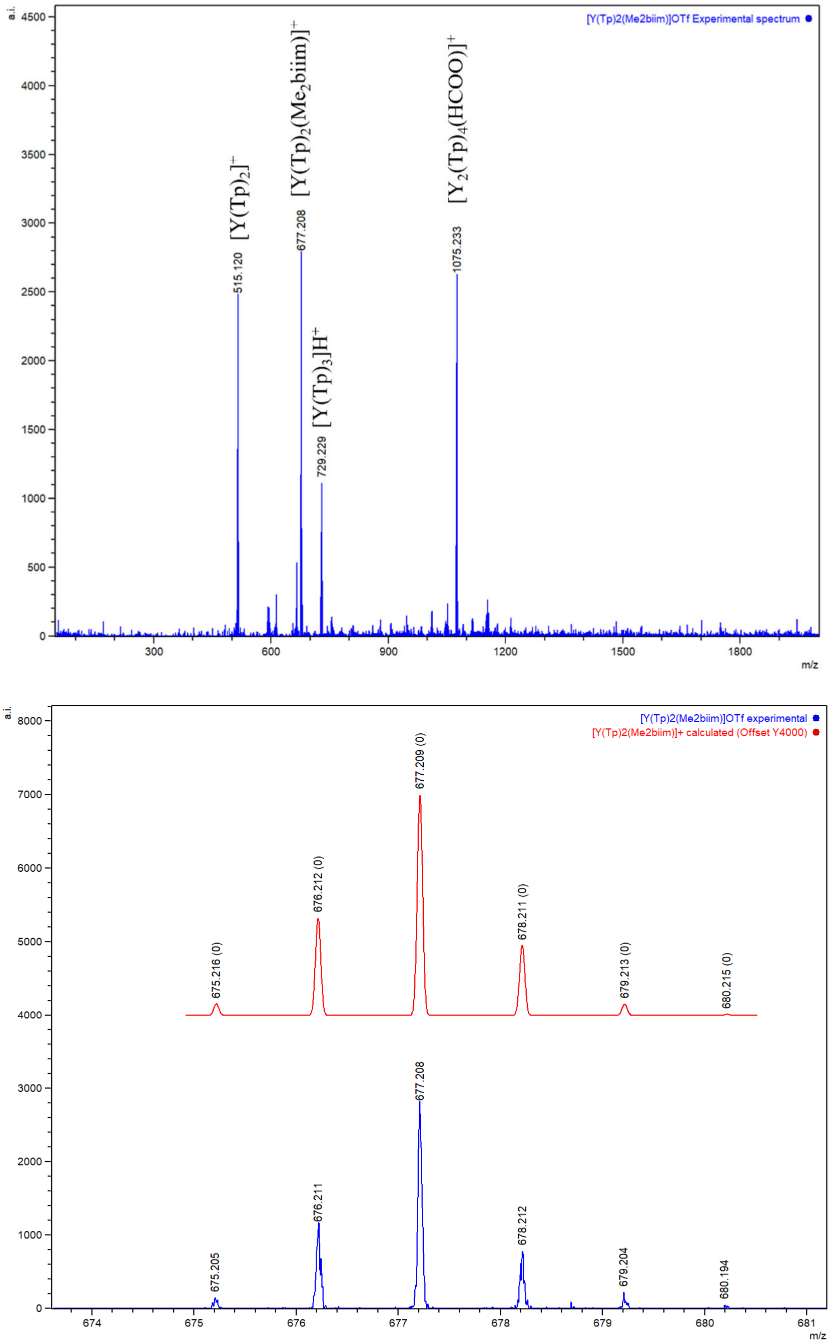

Figure S38. Top: ESI-HRMS full spectrum of a solution of [(Me_2biim)Y($Tp)_2$]OTf; solvent : CH_3CN; c = $5 \cdot 10^{-3}$M. Bottom: Zoom on the experimental HRMS spectrum (blue) and calculated signal of [(Me_2biim)Y($Tp)_2$]$^+$ (red). The presence of the peak for [$Ln_2(Tp)_4$(HCOO)]$^+$ in the MS is due to the presence of small quantities (0.1%) of formic acid added in the solvent used in HRMS to help to ionize organic molecules in solution. The presence of this peak does not reflect the real presence of this dimer in solution outside of the MS conditions

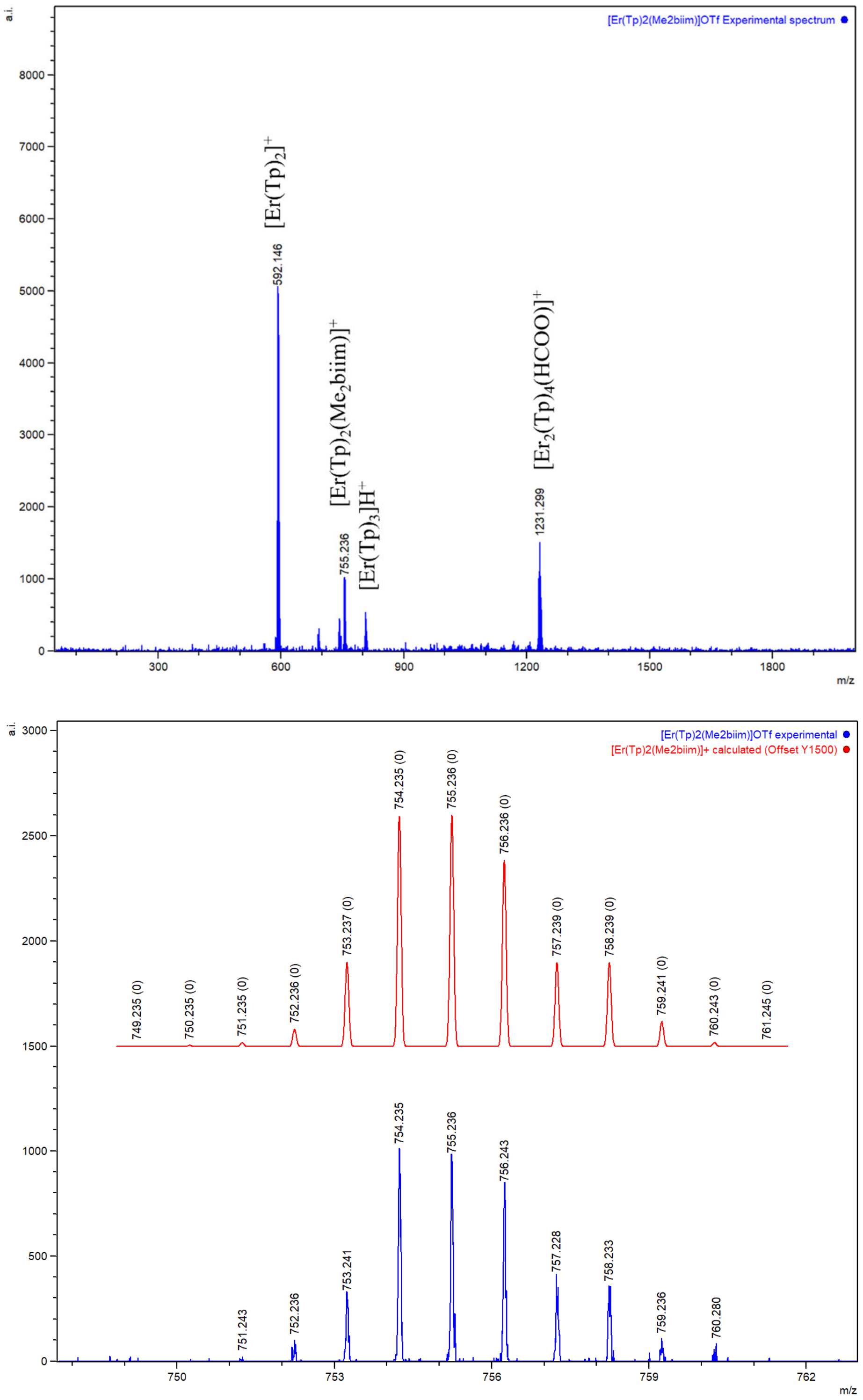

Figure S39. Top: ESI-HRMS full spectrum of a solution of $[Er(Tp)_2(Me_2biim)]OTf$; solvent : CH_3CN; c = $5 \cdot 10^{-3}$M. Bottom: Zoom on the experimental HRMS spectrum (blue) and calculated signal of $[Er(Tp)_2(Me_2biim)]^+$ (red). The presence of the peak for $[Ln_2(Tp)_4(HCOO)]^+$ in the MS is due to the presence of small quantities (0.1%) of formic acid added in the solvent used in HRMS to help to ionize organic molecules in solution. The presence of this peak does not reflect the real presence of this dimer in solution outside of the MS conditions

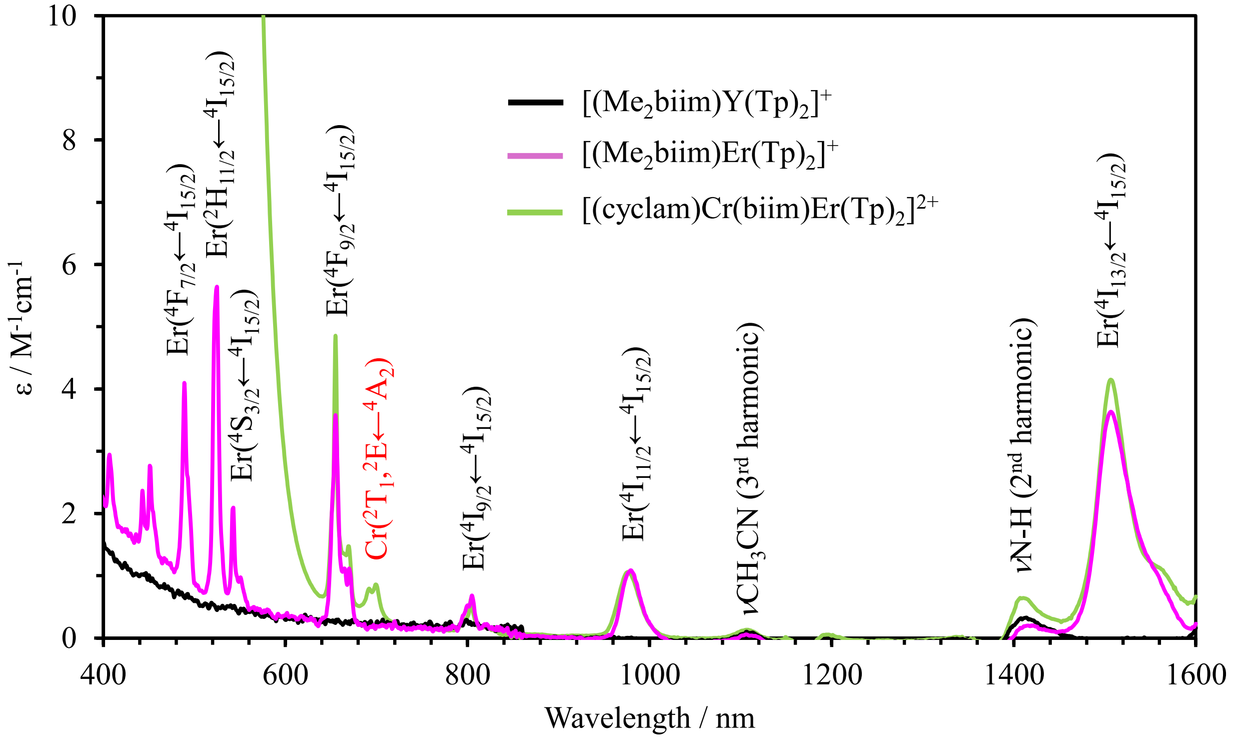

Figure S40. Absorption spectra of $[(Me_2biim)Y(Tp)_2]OTf$ (black trace), $[(Me_2biim)Er(Tp)_2]OTf$ (magenta trace) and $[(cyclam)Cr(biim)Er(Tp)_2]OTf_2$ (green trace) in acetonitrile, zoomed on the NIR part of the spectra, highlighting the Er(III) and Cr(III)-centered transitions.

Table S17. Radiative rate constants (k_{rad})^a in s^{-1} for the transitions centered on Cr(III) and Er(III) of the complexes studied in $[(cyclam)Cr(H_n biim)]^{(n+1)+}$, $[(cyclam)Cr(biim)Ln(Tp)_2]^{2+}$ (abbreviated as $[CrLn]^{2+}$) and $[(Me_2biim)Er(Tp)_2]^{+}$ (abbreviated as $[Er(Tp)_2]^{+}$).

Complex	$[(cyclam)Cr(H_2biim)]^{3+}$	$[(cyclam)Cr(Hbiim)]^{2+}$	$[CrY]^{2+}$	$[CrEu]^{2+}$	$[CrEr]^{2+}$	$[Er(Tp)_2]^{+}$
$^2E/^2T_1\leftarrow{}^4A_2$	137	135	175	153	247*	-
$^4I_{13/2}\leftarrow{}^4I_{15/2}$	-	-	-	-	318	320
$^4I_{11/2}\leftarrow{}^4I_{15/2}$	-	-	-	-	274	276
$^4I_{9/2}\leftarrow{}^4I_{15/2}$	-	-	-	-	151	196
$^4F_{9/2}\leftarrow{}^4I_{15/2}$	-	-	-	-	2019	1950
$^4S_{3/2}\leftarrow{}^4I_{15/2}$	-	-	-	-	994*	2666
$^2H_{11/2}\leftarrow{}^4I_{15/2}$	-	-	-	-	4343*	4141
$^4F_{7/2}\leftarrow{}^4I_{15/2}$					2256*	4736

^{a} The uncertainties on krad is typically ±5%. b krad value is only an estimation because to strong overlap of the transition with another absorption band, rendering difficult the accurate measure of. $\int \varepsilon(\tilde{\nu})\, d\tilde{\nu}$.

Supplementary File S5. Synthesis of Me4cyclam and its Cr(III) complexes.

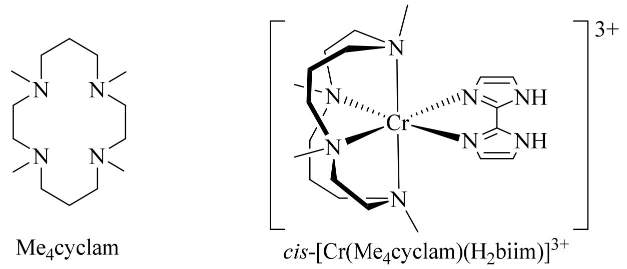

Figure S41. Chemical structure of the ligand 1,4,8,11-tetraaza-1,4,8,11-tetramethylcyclotetradecane (Me4cyclam) and the target Cr(III) complex *cis*-[Cr(Me4cyclam)(H2biim)]$^{3+}$.

The ligand Me4cyclam was obtained by methylating cyclam using formaldehyde and formic acid, following literature procedure [A5-1].

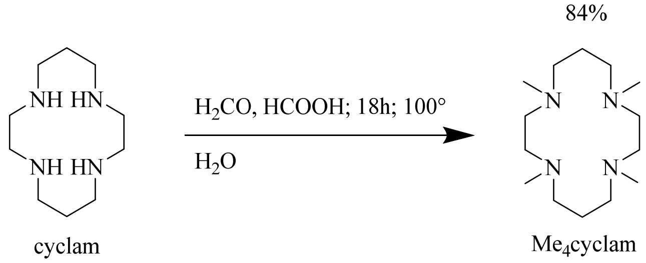

Figure S42. One step synthesis of Me4cyclam from cyclam, formaldehyde and formic acid, according to literature procedure [A5-1].

The next step consisted of reacting Me4cyclam with a Cr(III) salt to obtain the heteroleptic complex *cis*-[Cr(Me4cyclam)X2]X. However, any attempts using either $CrCl_3{\cdot}6H_2O$, anhydrous $CrCl_3$ or $CrBr_3{\cdot}6H_2O$ resulted in no reaction, or only protonation of the ligand. The only way to obtain the target product *cis*-[Cr(Me4cyclam)Cl2]Cl used $CrCl_3(THF)_3$ in strictly anhydrous conditions. However, as soon as the green [Cr(Me4cyclam)Cl2]Cl solid was filtered from the anhydrous solution, the solid hydrolyzed in less than a minute by absorbing moisture from the air, to form a black sticky paste consisting of the protonated ligand plus hydroxylated chromium salts visible using mass spectrometry.

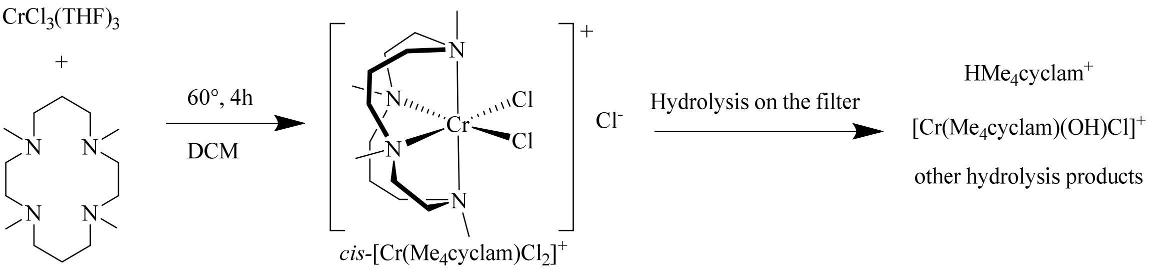

Figure S43. Failed attempt to isolate [Cr(Me4cyclam)Cl2]Cl, which hydrolyzed upon contact with humid atmosphere during filtration.

In addition, any attempts to react Me_4cyclam with a Cr(II) salt followed by oxidation also failed to produce the desired product. It was clear from these results that Me_4cyclam is not as good ligand as cyclam, and it would not resist the reaction with HOTf despite all the efforts implemented to avoid moisture. This might be caused by the steric hindrance of the tertiary amines of Me_4cyclam.

References

A5-1 Murphy, M. a; Malachowski, M. R. Composition, Synthesis and Therapeutic Applications of Polyamines. US2003013772A1, January 16, 2003. https://patents.google.com/patent/US20030013772A1/en

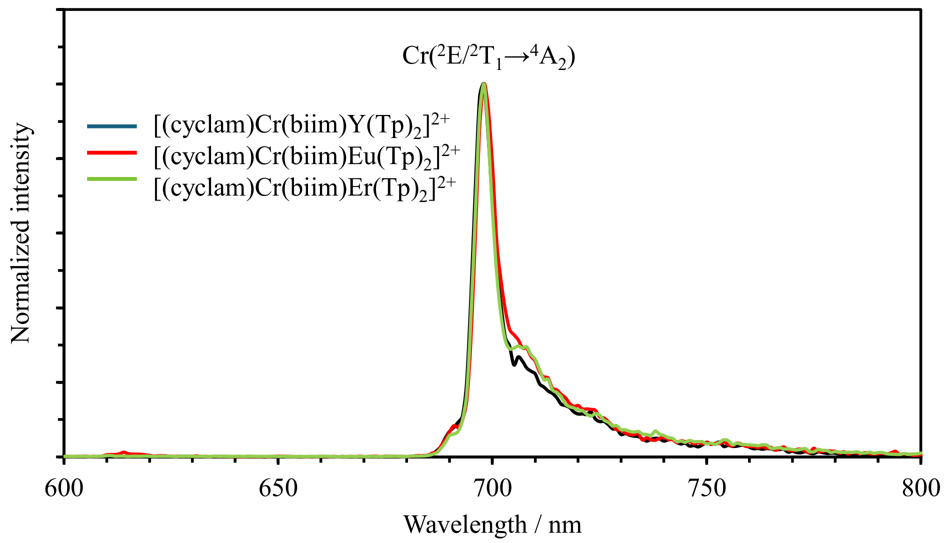

Figure S44. Emission spectra of $[(cyclam)Cr(biim)Ln(Tp)_2]^{2+}$(Ln = Y, Eu, Er) in the red region, upon excitation at 300 nm (350 nm for Ln = Er), *c* = 10^{-4} M (10^{-3} M for Ln = Er) in a mixture of CH_3CN/C_2H_5CN 6:4, 77 K.

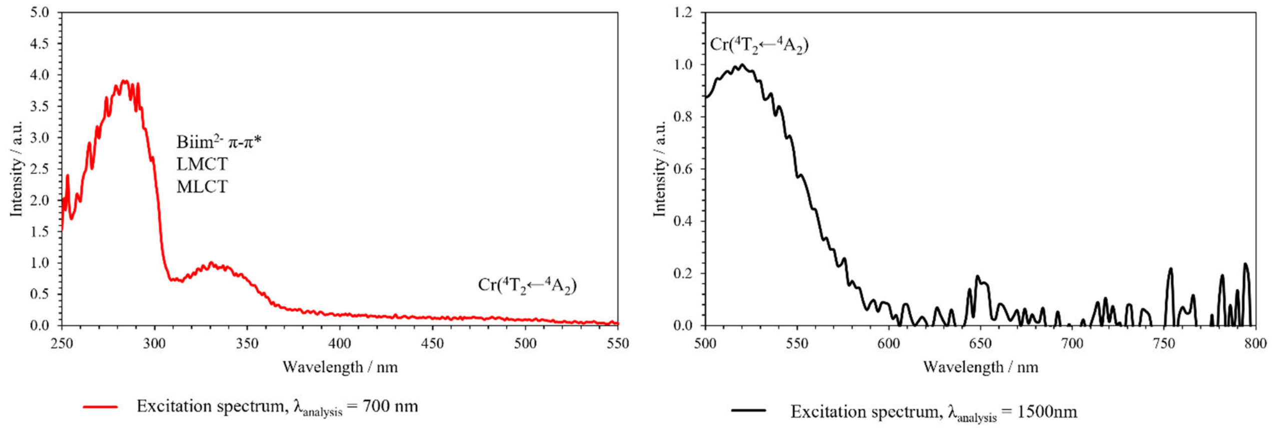

Figure S45. Excitation spectra of $[(cyclam)Cr(biim)Er(Tp)_2]OTf_2$ in CH_3CN at room temperature monitoring $Cr(^2E,^2T_1 \rightarrow ^4A_2)$ at λ_{em} = 700 nm (left, *c* = 10^{-5} M) and $Er(^4I_{13/2} \rightarrow ^4I_{15/2})$ at λ_{em} = 1500 nm (right *c* = 10^{-2} M (right). The second spectrum is cut below 500 nm due to high concentration of the solution (10^{-2} M), which causes primary inner filter effects that block the excitation light where the absorption is too high.

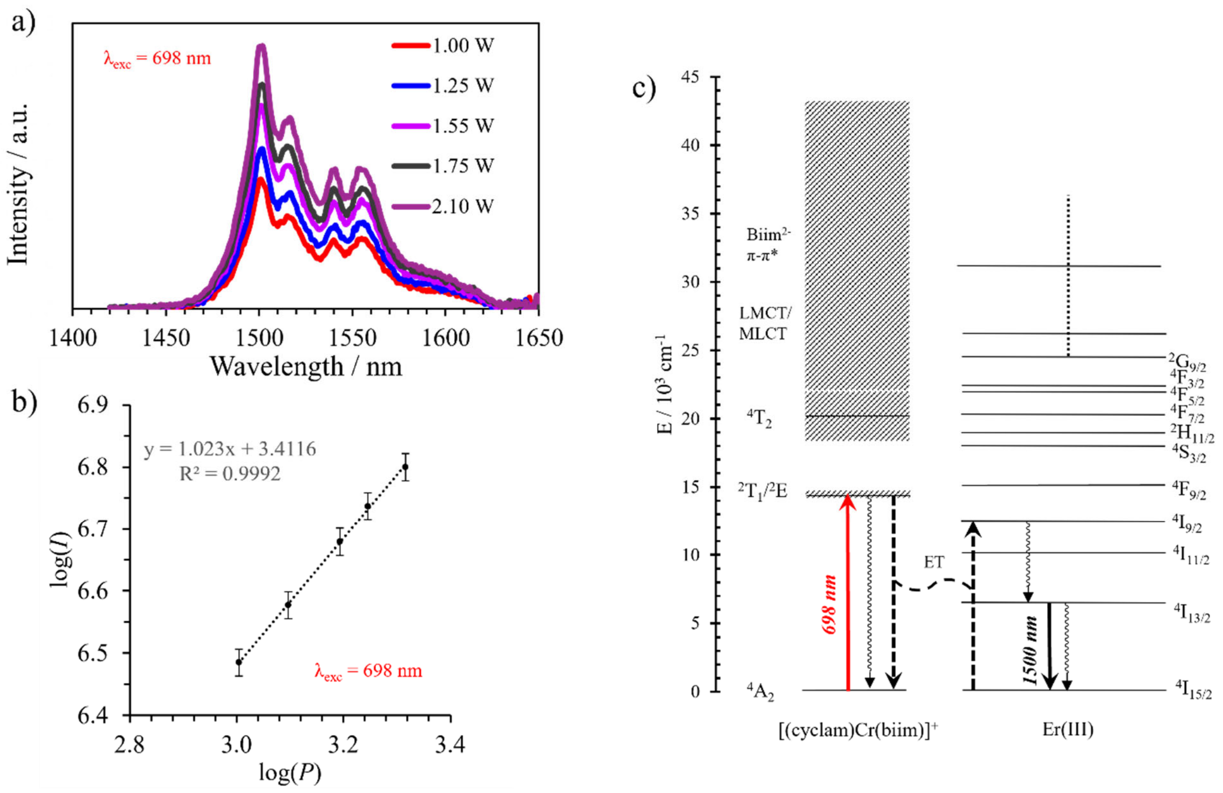

Figure S46. a) Er($^4I_{13/2}\rightarrow{}^4I_{15/2}$) emission spectra recorded at different excitation power for[(cyclam)Cr(biim)Er(Tp)$_2$]OTf$_2$ using a laser excitation at λ_{exc} = 698 nm. b) Logarithm of the integral of the emission intensity log(*I*) of the emission band of Er($^4I_{13/2}\rightarrow{}^4I_{15/2}$) as a function of the logarithm of the excitation power log(*P*) (error bars account for 5% error in the intensity). c) Jablonski diagram showing the energy levels of the Cr(III) complex and the Er(III) in [(cyclam)Cr(biim)Er(Tp)$_2$]$^{2+}$, radiative (straight arrows) and non-radiative transitions (wavy arrows) upon excitation a 698 nm laser. ET = energy transfer.

Supplementary File S6. Kinetic modeling of the excited state dynamics of complex [(cyclam)Cr(biim)Er(Tp)$_2$]$^{2+}$.

The emission lifetimes of the Cr(III)-based transitions have been measured for all complexes and are reported in Table 2.

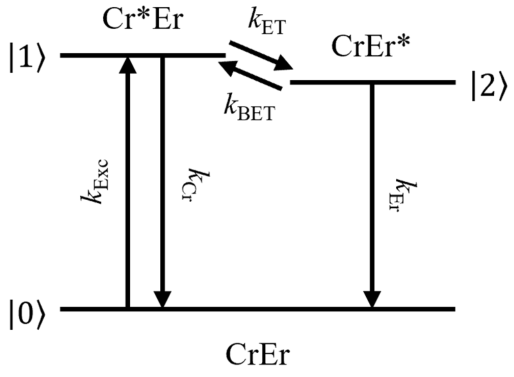

Figure S47. Simplified model showing the excited state dynamics of complex [(cyclam)Cr(biim)Er(Tp)$_2$]$^{2+}$. $|0\rangle$ represents the ground state, $|1\rangle$ represents the state where Cr(III) is in its excited state 2E, and $|2\rangle$ represents the state where Er(III) is its excited state. k_{Cr} is the total relaxation rate constant of the excited Cr(III) to the ground state (k_{rad} + $k_{non\text{-}rad}$), kEr is the total relaxation rate constant of the excited Er(III), k_{ET} is the energy transfer rate constant form the excited Cr(III) to the Er(III) and k_{BET} is the back-energy transfer rate constant form the excited Er(III) to the Cr(III).

The dynamic relative population $N^{|i\rangle}(t)$ of each state i of any molecular system S$_n$A$_m$ containing a discrete number of activators (A) and sensitizers (S) can be modeled with a set of linear differential equations written in the matrix form[A6-1],[A6-2]

$$\left[\frac{dN^{|i\rangle}}{dt}\right] = M \times \left[N^{|i\rangle}\right] \tag{A6-1}$$

With the energy diagram described in Figure S47, equation A6-1 can be written

$$\begin{bmatrix} \frac{dN^{|0\rangle}(t)}{dt} \\ \frac{dN^{|1\rangle}(t)}{dt} \\ \frac{dN^{|2\rangle}(t)}{dt} \end{bmatrix} = \begin{bmatrix} -k_{exc} & k_{Cr} & k_{Er} \\ k_{exc} & -k_{ET} - k_{Cr} & k_{BET} \\ 0 & k_{ET} & -k_{Er} - k_{BET} \end{bmatrix} \times \begin{bmatrix} N^{|0\rangle}(t) \\ N^{|1\rangle}(t) \\ N^{|2\rangle}(t) \end{bmatrix} \tag{A6-2}$$

Just after the short excitation pulse, t is fixed to zero and kexc becomes zero. We therefore consider that all the molecules that have been excited lie in the $|1\rangle$ state and initial conditions become

$$\begin{bmatrix} N^{|0\rangle}(0) \\ N^{|1\rangle}(0) \\ N^{|2\rangle}(0) \end{bmatrix} = \begin{bmatrix} 0 \\ 1 \\ 0 \end{bmatrix} \tag{A6-2}$$

The solution of the differential equation A6-2 is given by the *Lagrange-Sylvester* formula [A6-1]:

$$\begin{bmatrix} N^{|0\rangle}(t) \\ N^{|1\rangle}(t) \\ N^{|2\rangle}(t) \end{bmatrix} = \sum_{i=1}^{3} e^{\lambda_i t} \mathrm{P}_i \times \begin{bmatrix} N^{|0\rangle}(0) \\ N^{|1\rangle}(0) \\ N^{|2\rangle}(0) \end{bmatrix} = \sum_{i=1}^{3} e^{\lambda_i t} \mathrm{P}_i \times \begin{bmatrix} 0 \\ 1 \\ 0 \end{bmatrix} \quad \text{(A6-3)}$$

where Pi are the projection operator given by:

$$\mathrm{P}_i = \frac{\prod_{\substack{j=1 \\ j\neq i}}^{3} \left\{ \begin{pmatrix} 0 & k_{\mathrm{Cr}} & k_{\mathrm{Er}} \\ 0 & -k_{\mathrm{ET}} - k_{\mathrm{Cr}} & k_{\mathrm{BET}} \\ 0 & k_{\mathrm{ET}} & -k_{\mathrm{Er}} - k_{BET} \end{pmatrix} - \lambda_j \begin{pmatrix} 1 & 0 & 0 \\ 0 & 1 & 0 \\ 0 & 0 & 1 \end{pmatrix} \right\}}{\prod_{\substack{j=1 \\ j\neq i}}^{3} (\lambda_i - \lambda_j)} \quad \text{(A6-4)}$$

and λ_i are the eigenvalues of the kinetic matrix M.

One can show that the eigenvalues of the matrix M are the following:

$$\lambda_1 = 0 \quad \text{(A6-5a)}$$

$$\lambda_{2,3} = \frac{(k_{\mathrm{ET}} + k_{\mathrm{Cr}} + k_{\mathrm{Er}} + k_{\mathrm{BET}}) \pm \sqrt{(k_{\mathrm{ET}} + k_{\mathrm{Cr}} + k_{\mathrm{Er}} + k_{\mathrm{BET}})^2 - 4(k_{\mathrm{ET}} k_{\mathrm{Er}} + k_{\mathrm{Cr}} k_{\mathrm{Er}} + k_{\mathrm{Cr}} k_{\mathrm{BET}})}}{2} \quad \text{(A6-5b)}$$

Introduction of equation A6-5 into A6-4 provides the projection operators Pi required for modeling the population of Cr*Er excited level, i.e. $N^{|1\rangle}(t)$, which follows a biexponential decay:

$$N^{|1\rangle}(t) = A_1 e^{-\left(\frac{t}{\tau_1}\right)} + A_2 e^{-\left(\frac{t}{\tau_2}\right)} \quad \text{(A6-6)}$$

$$\text{with } \tau_1 = -\frac{1}{\lambda_2} \; ; \; \tau_2 = -\frac{1}{\lambda_3} \quad \text{(A6-7)}$$

A1 and A2 are deduced from equation A6-3.

k_{Cr} is known thanks to the emission lifetime measurement of the Cr-Y compound, which was measured to be $4.0 \cdot 10^6$ s^{-1}. The three other constants k_{Er}, k_{ET}, and k_{BET} are unknown. To estimate their value, the relative population $N^{|1\rangle}(t)$ computed from equation A6-3 was fitted to the experimental emission decay curve by optimizing k_{Er}, k_{ET}, and k_{BET} using non-linear least square techniques (Figure S46). The following constants were found:

Table S18. Relaxation and energy transfer rate constants obtained by fitting $N^{|1\rangle}(t)$ with the experimental emission decay curve (see Figure S46).

k_{Cr} / s^{-1}	k_{Er} / s^{-1}	k_{ET} / s^{-1}	k_{BET} / s^{-1}
$4.0 \cdot 10^6$	$1.2 \cdot 10^6$	$4.3 \cdot 10^6$	$2.5 \cdot 10^6$

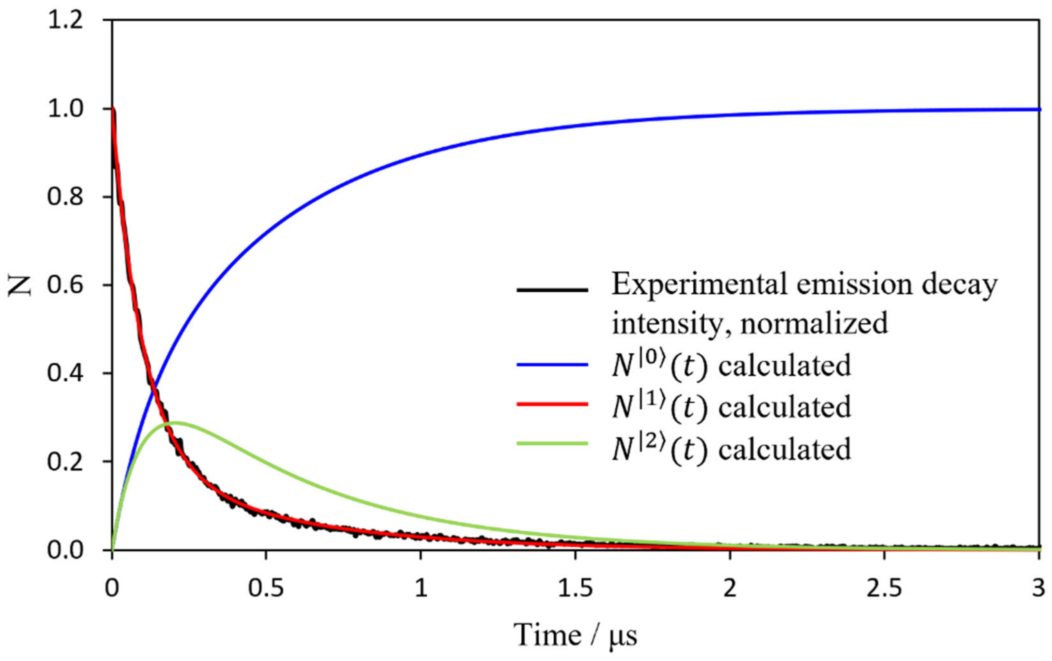

Figure S48. Population densities $N^{|i\rangle}(t)$ calculated from the rate constants in Table S18 using equation A6-3, compared to the normalized experimental emission decay curve of [(cyclam)Cr(biim)Er(Tp)2]2+ at λem = 700 nm (λexc = 480 nm, c = 10-2 M in CH3CN, 293 K). $N^{|1\rangle}(t)$ follows a biexponential decay with A1=0.792, A2=0.208, τ1=101 ns, τ2=512 ns.

References

A6-1 Starzak, M. E. Mathematical Methods in Chemistry and Physics, Plenum press New York, pp 289-357 (1989).

A6-2 Suffren, Y.; Zare, D.; Eliseeva, S. V.; Guénée, L.; Nozary, H.; Lathion, T.; Aboshyan-Sorgho, L.; Petoud, S.; Hauser, A.; Piguet, C. Near-Infrared to Visible Light-Upconversion in Molecules: from Dream to Reality. *J. Phys. Chem. C* **2013**, *117*, 26957-26963.

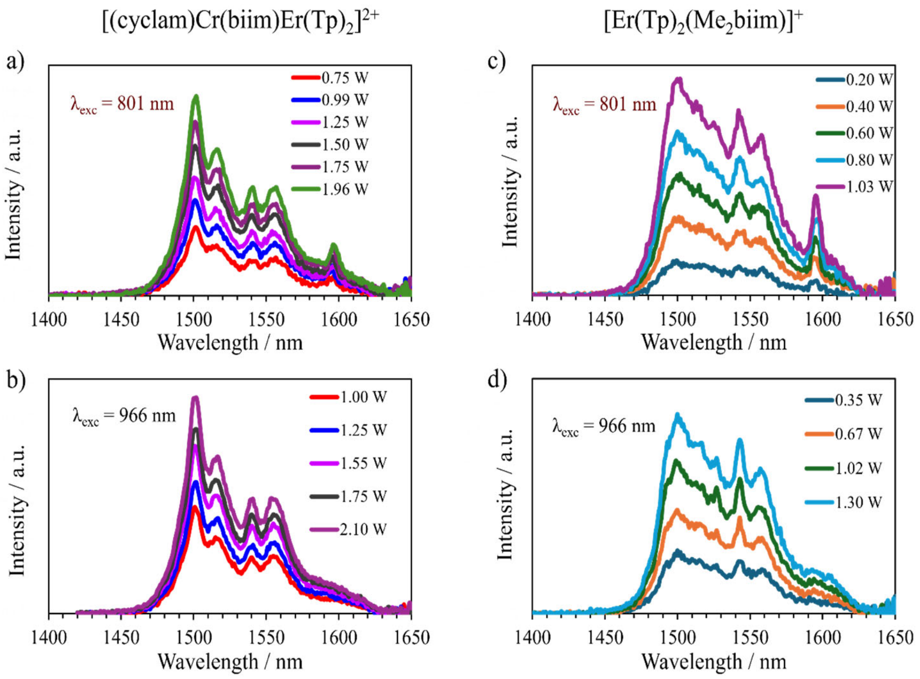

Figure S49. Emission spectra in the infrared at different excitation power of [(cyclam)Cr(biim)Er(Tp)2]OTf2 with laser excitation at a) λ_{exc} = 801 nm, b) λ_{exc} = 966 nm and [Er(Tp)2(Me2biim)]OTf with laser excitation at c) λ_{exc} = 801 nm and d) λ_{exc} = 966 nm ($c = 10^{-2}$ M in CH_3CN at room temperature).

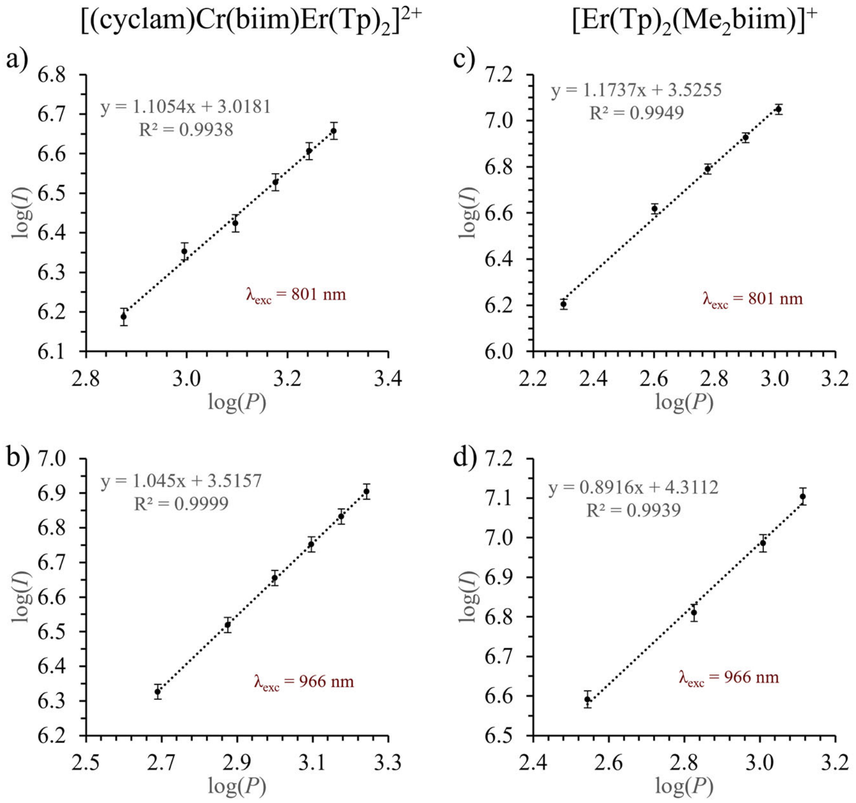

Figure S50. Logarithm of the integral of the emission intensity log(I) of the emission band of the $Er(^4I_{13/2}\rightarrow{}^4I_{15/2})$ emission at 1550 nm as a function of the logarithm of the excitation power log(P) for [(cyclam)Cr(biim)Er(Tp)2]OTf2 with laser excitation at a) λ_{exc} = 801 nm, b) λ_{exc} = 966 nm and

[Er(Tp)2(Me2biim)]OTf with laser excitation at c) λ_{exc} = 801 nm and d) λ_{exc} = 966 nm. (c = 10^{-2} M in CH_3CN at room temperature. Error bars account for 5% error in the intensity).

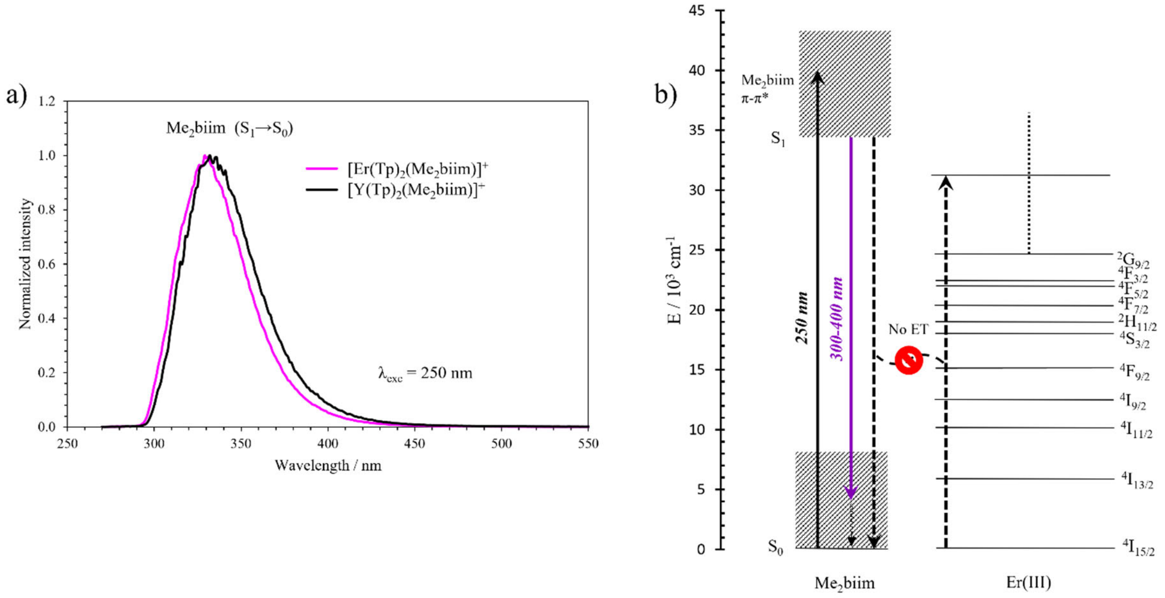

Figure S51. a) Emission spectrum of [Er(Tp)2(Me2biim)]OTf in the UV-Vis region, upon lamp excitation at 250 nm, c = 10^{-2} M in CH_3CN, room temperature. b) Jablonski diagram showing the energy levels of Me2biim and the Er(III) in $[Er(Tp)_2(Me_2biim)]^+$ radiative (straight arrows) and non-radiative transitions (wavy arrows) upon excitation at 250 nm.

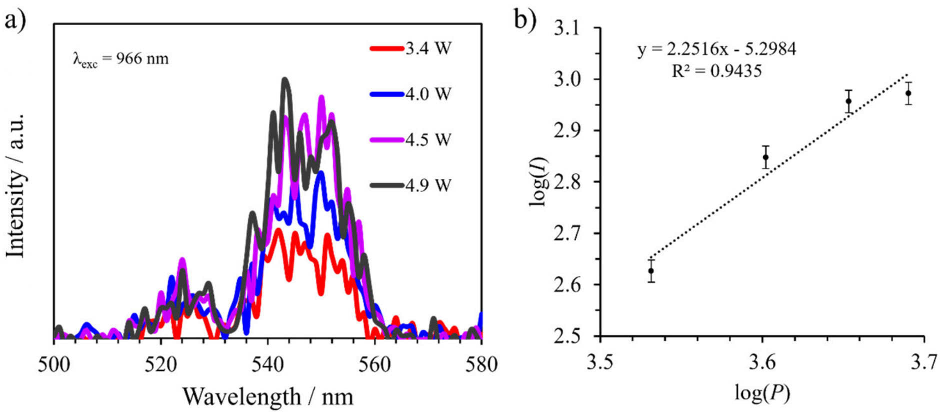

Figure S52. Upconversion spectra in the visible at different excitation power of [(cyclam)Cr(biim)Er(Tp)2]OTf2 with laser excitation at λ_{exc} = 966 nm, at room temperature (c = 10^{-2} M in CH_3CN) b) Logarithm of the integral of the emission intensity log(I) of the upconversion signal as a function of the logarithm of the excitation power log(P) of [(cyclam)Cr(biim)Er(Tp)2]OTf2 with laser excitation at λ_{exc} = 966 nm; error bars account for 5% error in the intensity.

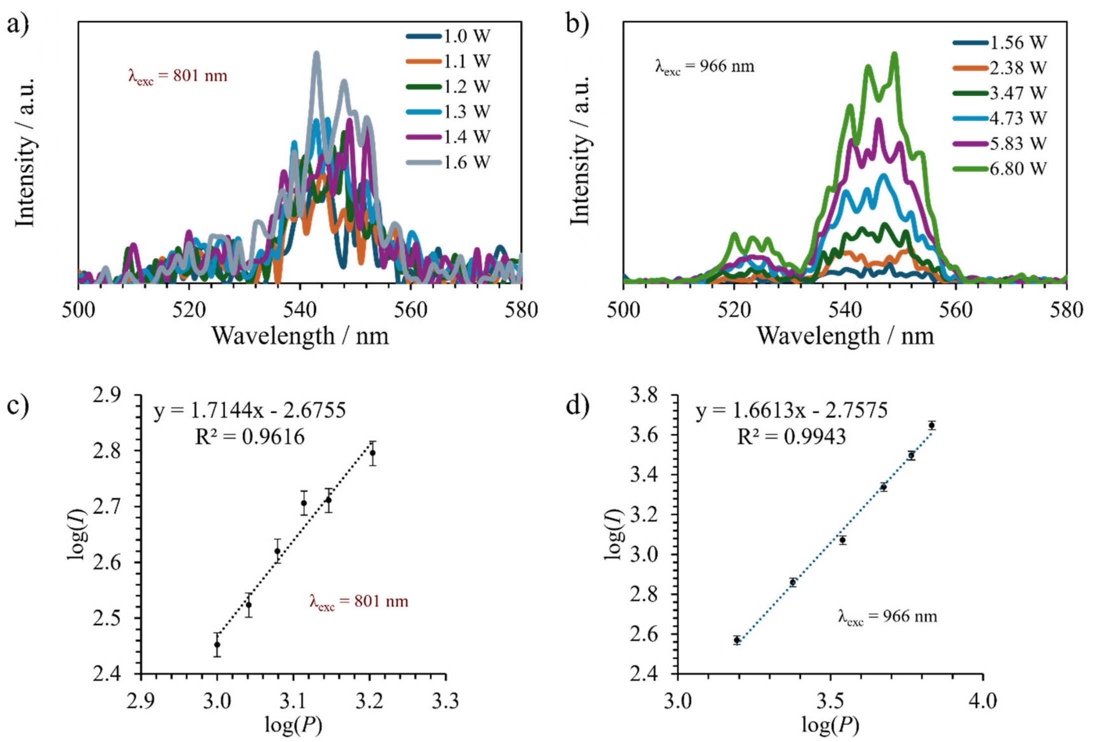

Figure S53. Upconversion spectra in the visible at different excitation power of [(Me$_2$biim)Er(Tp)$_2$]OTf at room temperature (c = 10^{-2} M in CH_3CN) with laser excitation at a) λ_{exc} = 966 nm and b) λ_{exc} = 801 nm. Logarithm of the integral of the emission intensity log(I) of the upconversion signal as a function of the logarithm of the excitation power log(P) of [(Me$_2$biim)Er(Tp)$_2$]OTf with laser excitation at c) λ_{exc} = 966 nm and d) λ_{exc} = 801 nm. Error bars account for 5% error in the intensity.

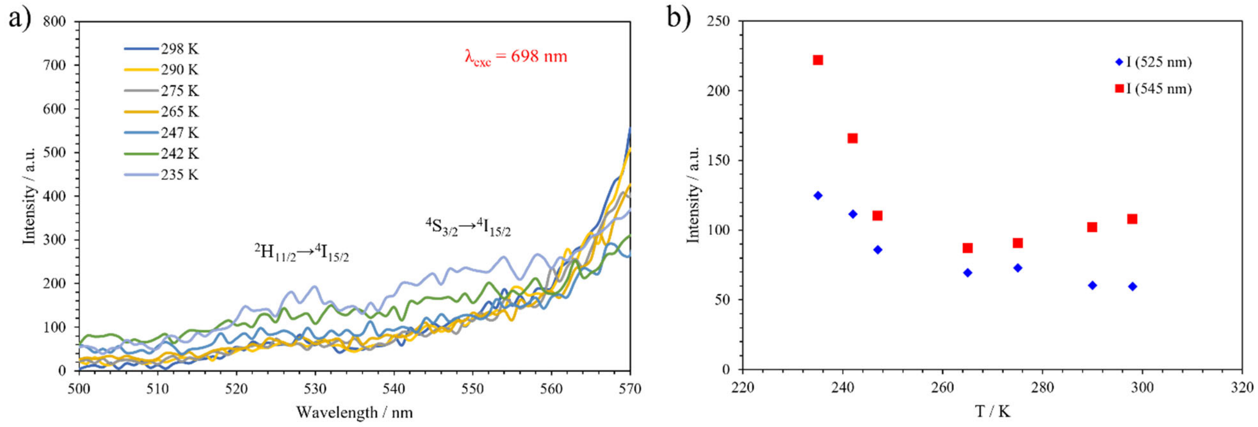

Figure S54. a) Upconversion spectra of [(cyclam)Cr(biim)Er(Tp)$_2$]OTf$_2$ at different temperatures upon excitation with 698 nm laser; P = 2.2 W; c = 10^{-2} M in CH_3CN. b) Emission intensity at 525 nm (blue) and 545 nm (red) as function of the temperature.
